# Developing Evidence to Support Policy: Protocol for the StrAtegic PoLicy EvIdence-Based Evaluation CeNTer (SALIENT)

**DOI:** 10.2196/59830

**Published:** 2024-09-19

**Authors:** Mary Jo Pugh, Jolie N Haun, P Jon White, Gerald Cochran, April F Mohanty, Lisa M McAndrew, Adam J Gordon, Richard E Nelson, Megan E Vanneman, Diana E Naranjo, Rachel C Benzinger, Audrey L Jones, Jacob Kean, Susan L Zickmund, Angela Fagerlin

**Affiliations:** 1 VA Salt Lake City Health Care System Informatics, Decision-Enhancement and Analytic Sciences Center Salt Lake City, UT United States; 2 Division of Epidemiology, Department of Internal Medicine Spencer Fox Eccles School of Medicine The University of Utah Salt Lake City, UT United States; 3 James A. Haley Veterans' Hospital Research and Development Service Tampa, FL United States; 4 War Related Illness and Injury Study Center Veterans Affairs New Jersey Healthcare System East Orange, NJ United States; 5 Department of Population Health Sciences Spencer Fox Eccles School of Medicine The University of Utah Salt Lake City, UT United States; 6 VA Informatics and Computing Infrastructure Salt Lake City, UT United States

**Keywords:** evidence-based policy-making, policy evaluation, knowledge translation, veterans, implementation science

## Abstract

**Background:**

All federal agencies are required to support appropriation requests with evidence and evaluation (US Public Law 115-435; the Evidence Act). The StrAtegic PoLicy EvIdence-Based Evaluation CeNTer (SALIENT) is 1 of 6 centers that help the Department of Veterans Affairs (VA) meet this requirement.

**Objective:**

Working with the existing VA evaluation structure, SALIENT evaluations will contribute to (1) optimize policies and programs for veteran populations; (2) improve outcomes regarding health, equity, cost, and provider well-being; (3) advance the science of dissemination and knowledge translation; and (4) expand the implementation and dissemination science workforce.

**Methods:**

We leverage the Lean Sprint methodology (iterative, incremental, rule-governed approach to clearly defined, and time-boxed work) and 3 cores to develop our evaluation plans collaboratively with operational partners and key stakeholders including veterans, policy experts, and clinicians. The Operations Core will work with evaluation teams to develop timelines, facilitate work, monitor progress, and guide quality improvement within SALIENT. The Methods Core will work with evaluation teams to identify the most appropriate qualitative, quantitative, and mixed methods approaches to address each evaluation, ensure that the analyses are conducted appropriately, and troubleshoot when problems with data acquisition and analysis arise. The Knowledge Translation (KT) Core will target key partners and decision makers using a needs-based market segmentation approach to ensure that needs are incorporated in the dissemination of knowledge. The KT Core will create communications briefs, playbooks, and other materials targeted at these market segments to facilitate implementation of evidence-based practices and maximize the impact of evaluation results.

**Results:**

The SALIENT team has developed a center infrastructure to support high-priority evaluations, often to be responsive to shifting operational needs and priorities. Our team has engaged in our core missions and operations to rapidly evaluate a high-priority areas, develop a comprehensive Lean Sprint systems redesign approach to training, and accelerate rapid knowledge translation.

**Conclusions:**

With an array of interdisciplinary expertise, operational partnerships, and integrated resources, SALIENT has an established and evolving infrastructure to rapidly develop and implement high-impact evaluations. Projects are developed with sustained efficiency approaches that can pivot to new priorities as needed and effectively translate knowledge for key stakeholders and policy makers, while creating a learning health system infrastructure to foster the next generation of evaluation and implementation scientists.

**International Registered Report Identifier (IRRID):**

PRR1-10.2196/59830

## Introduction

### Background and Center’s Overall Goal

The US Public Law 115-435—The Evidence Act—requires the Department of Veterans Affairs (VA) to support appropriation requests with evidence and evaluation [1]. The Veterans Health Administration, Office of Research and Development, Quality Enhancement Research Initiative (QUERI), developed the Evidence-based Policy Evaluation Center Award mechanism to develop an infrastructure of centers that specialize in rigorous, independent evaluations focused on national priorities that are informed by the Foundations for Evidence-Based Policymaking Act (Evidence Act, US PL 115-435) of 2018 (Multimedia Appendix 1).

The current protocol presents the proposed StrAtegic PoLicy EvIdence-Based Evaluation CeNTer (SALIENT), with the goal to conduct evaluations that support the adoption of programs and policies aligned with VA priorities and VA’s goal to be a high-reliability organization and learning health system. SALIENT will generate, disseminate, and implement evidence-based policy recommendations and best practices in accordance with the Evidence Act. To accomplish this, we propose 4 objectives. For objective 1, the SALIENT team will develop and conduct comprehensive evaluations using a wide range of approaches (quantitative, qualitative, mixed methods, policy analysis, economic, clinical informatics, and implementation science) specifically tailored to VA’s strategic mission goals and priority areas. For objective 2, the SALIENT team will develop and deploy knowledge translation resources such as communication briefs, playbooks, and other materials for key stakeholders to accelerate the implementation of evidence-based practices and impact. For objective 3, the SALIENT team will use evaluation methods to develop best practices in policy evaluation and implementation. For objective 4, the SALIENT team will train diverse evaluation scientists to democratize evaluation, implementation, and knowledge translation expertise.

In 2022, to align with 2021-2025 QUERI strategic goals and accelerate rapid knowledge translation in the pursuit of transforming VA into to a learning health system, QUERI launched an initiative to include a funding mechanism for evidence-based policy evaluation centers to support innovation in policy evaluation [2,3]. SALIENT was selected to support the enterprise-wide effort in policy planning and evaluation, due to the team’s capability to foster advancements in VA priority areas.

### SALIENT Areas of Expertise Related to FY23 Evaluation Priorities

Our interdisciplinary team has broad and deep expertise across VA evaluation priorities. Table 1 illustrates our expertise in these priority areas and details resources of our multisite center, including leadership from the Informatics, Decision Enhancement, & Analytics Sciences Center and James A. Haley Veterans’ Hospital, and collaborators from the Center of Innovation for Complex Chronic Healthcare (Hines VA), the Center for Innovation for Veteran-Centered and Value-Driven Care, the Center for Care Delivery and Outcomes Research, and the War Related Illness and Injury Study Center. This expertise uniquely positions us to address 6 national evaluation priorities, identified by QUERI’s multistakeholder process, that are linked to Veterans Health Administration Performance Plan metrics (eg, Strategic Analytics for Improvement and Learning).

**Table 1:** StrAtegic PoLicy EvIdence-Based Evaluation CeNTer investigator expertise in Quality Enhancement Research Initiative 2023 priority areas.

Priority area	SALIENT^a^ investigators
Military environmental exposures	Hunt, McAndrew, Mohanty, Samore, Knight, Smith, and *Pugh*^b^
Integration of care	Gordon, Hunt, Butler, Brooke, Nelson, McAndrew, A Jones, and *Haun*^b^
Improve long-term care, aging in place, home-based services, and so forth	Bouldin, Rupper, Butler, French, and *Pugh*^b^
Strategies to mitigate impact of COVID-19	Samore, M Jones, McAndrew, Hunt, Mohanty, Knight, Smith, and *White*^b^
Assess quality and cost of VA^c^-purchased community care	Vanneman, Smith, and *Haun*^b^
Assess MISSION Act standards of care and impacts on QI^d^ and policy changes	Vanneman and *White*^b^

^a^SALIENT: StrAtegic PoLicy EvIdence-Based Evaluation CeNTer.

^b^Italicized names indicate SALIENT multiple principal investigators leading each priority area.

^c^VA: Department of Veterans Affairs.

^d^QI: Quality improvement.

### Evaluate Strategies and Inform Policy to Mitigate Military Environmental Exposures on Veterans

SALIENT investigators are uniquely positioned to evaluate environmental exposures [4], including airborne hazards [5], which are high priorities of the President and Congress [6]. Our team conducted foundational studies to document the impact of military exposures and improve health care, demonstrating our operational partnerships and “big data” resources. This includes pioneering the use of the electronic health record to examine exposure concerns, that is, chronic multisymptom illness (AFM, LMM, and MHS), and conducting the first hybrid effectiveness implementation trial for exposure concerns: chronic multisymptom illness (LMM) [7-11]. VA’s Health Outcomes Military Exposure program office called on us to lead the use of the electronic health record and individual longitudinal exposure record to connect phenotypes with military exposures (MJP), evaluate Health Outcomes Military Exposure/ War Related Illness and Injury Study Center initiatives and clinical services (AFM and LMM), and lead and support educational efforts (LMM and SCH).

### Optimize Integration of Care for Primary, Mental Health, Specialty, and Urgent Care Services

SALIENT is uniquely positioned to evaluate and optimize care for veterans with complex conditions that require integration of care across providers. Dr Gordon leads the Vulnerable Veteran Innovative Patient-Aligned Care Team Service & Research, Veteran Innovative Patient-Aligned Care Team Initiative, which has developed and implemented integrative care models for vulnerable veterans, including the implementation of medication treatment for opioid use disorder in 36 VA facilities across the nation (SCOUTT). Dr Brooke implemented a multidisciplinary program, the Transitional Pain Service, in Veterans Integrated Services Networks-19 coordinating pain management for patients at risk for opioid use disorder at the time of surgery [12,13]. This includes implementation for primary care providers to identify and refer high-risk patients to Transitional Pain Service. Our team is also a leader in integrating care for exposures into primary care. Dr Hunt is the national director of the postdeployment integrated care initiative, which is the office of primary care initiative to integrate deployment and exposure care into primary care, and Dr McAndrew is conducting multiple evaluations of education and consultation services to support primary care in addressing these concerns.

### Improve Long-Term Care, Aging in Place, Geriatric Care, and Home Care Service

National efforts have focused on rebalancing long-term support services to promote care for veterans in their homes rather than institutional settings. This priority area is aligned with our expertise leading the Data and Policy Core of the Elizabeth Dole Center of Excellence for Veteran and Caregiver Research (Dole CoE). The Dole CoE helps veterans age in place. The Dole CoE team works with operations partners in Geriatric Extended Care to identify health services trajectories associated with quality home care and those predicting institutionalization using Geriatric Extended Care–Data Analysis Center VA Medicare and national survey data. The data resources (Multimedia Appendix 2) and expertise of SALIENT investigators in evaluation and geriatrics [14] are a strong foundation to address this VA priority.

### Evaluate Strategies to Mitigate the Long-Term Impact of COVID-19 Including Reducing Adverse Outcomes Associated With Delayed or Suppressed Care

SALIENT investigators have played an instrumental role in the COVID-19 response for multiple federal agencies and local and state health departments [15,16]. Our contributions to efforts to mitigate the adverse impacts of COVID-19 highlight our expertise in epidemiology and control of infectious diseases. We developed new data resources for case ascertainment, clinical classification, and assessment of outcomes [17-19]. Combining clinical expertise with advanced analytical techniques, we have addressed key questions around short- and long-term management of COVID-19 infection [20]. We lead studies funded by the Food and Drug Administration and the Centers for Disease Control and Prevention on veteran COVID-19–related health inequities. In VA Health Systems Research (HSR), QUERI, and Department of Defense funded studies, we use mixed methods to evaluate problems of vaccine hesitancy and trust, and the impact of COVID-19 on veterans’ health and well-being [21]. Building on our HSR-funded work on colon cancer screening, we evaluated the impact of the pandemic on veteran access to endoscopy [22,23]. Working with our VA operational partners, the SALIENT team proposed the COVID Post-Exposure Evaluation and Symptomatology Center, which addresses critical knowledge gaps concerning the post–acute sequelae of COVID-19 (ie, post–COVID-19 condition) and underscores our focus on advancing COVID-19 research and improving care for veterans living with COVID-19.

### Assess and Improve the Quality and Cost of VA-Purchased Community Care, Including Enhancing Community Care and Digital Care Coordination to Improve Veteran Health

SALIENT is uniquely positioned to evaluate quality and cost of VA-purchased community care across a broad set of domains, including mental health and disparities. Dr Vanneman is a VA HSR Career Development Award recipient and a national expert in measuring access, quality, and cost of VA-purchased community care, with an emphasis on access, patient experience, and behavioral health [24-31]. Dr Vanneman is a multiple principal investigator (MPI) of the Access and Community Care Engagement Network Team COnsortium of REsearch. Dr Haun leads research and operations-based projects on implementation and evaluation of digital care resources to improve VA employee workflows and care coordination for veterans and their informal caregivers. Her work has driven the implementation and redesign of My HealtheVet, Secure Messaging [32], and the Veterans’ Delegation tool, and extended integration of digital resources in primary care [32-38].

### Assess MISSION Act Standards of Care and Impacts on Quality Improvement and Policy Changes

The MISSION Act (Section 1703C of title 38, Section 104) required VA to establish standards for quality for VA and VA-purchased community care health care services [39]. Published studies, including those by Dr Vanneman and collaborators, show similar or better quality of care at the national level for VA-delivered versus VA-purchased community care and are consistent with findings from prior studies that have compared VA and non-VA care (eg, Medicare). As Dr Vanneman is one of the few HSR investigators with extensive expertise in policy and analysis of community care data, SALIENT is well positioned to evaluate MISSION Act standards of care and impacts on quality improvement and policy.

### SALIENT Is an Integral Part of the Existing VA Program Evaluation Structure

**Figure 1:**
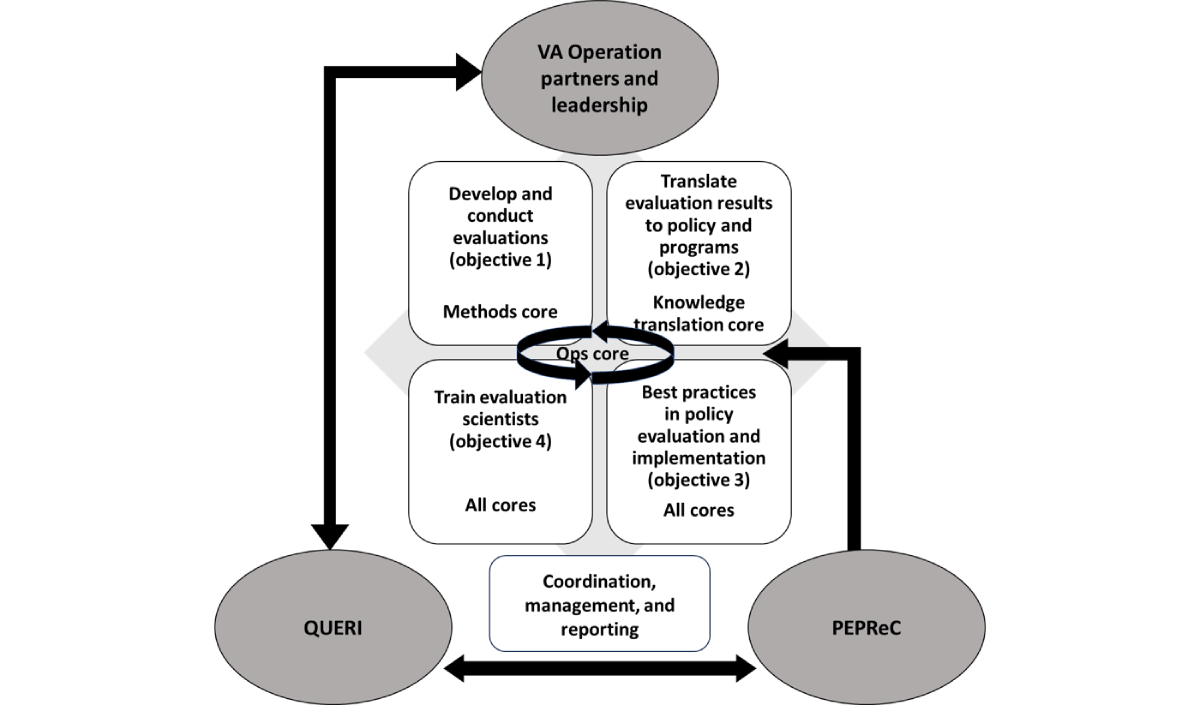
Interrelationships of Veterans Affairs operations and leadership, Quality Enhancement Research Initiative, the Partnered Evidence-based Policy Resource Center, and StrAtegic PoLicy EvIdence-Based Evaluation CeNTer (SALIENT) stakeholders. PEPReC: Partnered Evidence-Based Policy Resource Center; QUERI: Quality Enhancement Research Initiative; VA: Department of Veterans Affairs.

Figure 1 shows the interrelationships of VA operations and leadership, QUERI, the Partnered Evidence-Based Policy Resource Center (PEPReC), and SALIENT stakeholders.

We will work with these partners and other evaluation centers to conduct evaluations that support VA programs and policies. We will continually communicate research findings to other VA evaluators and synthesize learning across SALIENT and other groups to enhance the quality of evaluations conducted by the VA more broadly and develop best practices in policy implementation, impact, and evaluation.

## Methods

### Guiding Frameworks

**Figure 2:**
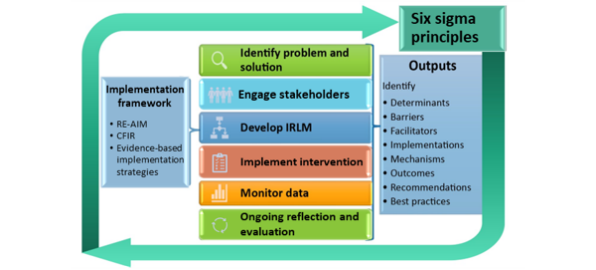
StrAtegic PoLicy EvIdence–Based Evaluation CeNTer (SALIENT) conceptual framework for conducting evaluation. CFIR: Consolidated Framework for Implementation Research; IRLM: Implementation Research Logic Model; RE-AIM: Reach, Effectiveness, Adoption, Implementation, Maintenance.

SALIENT will conduct evaluations that support the adoption of programs and policies aligned with VA priorities and its goal to be a national learning health system. Our evaluation approach is anchored by key tenets of implementation science, quality improvement, and learning health systems (Figure 2).

Following the steps outlined by the QUERI Implementation Roadmap [40], and ACTION (Alignment with multilevel priorities, Commitment from operational partners, Tailoring to the local context, Informing the field, Observing health care changes, and generating New questions or projects) Impact Framework [41], SALIENT researchers will (1) identify problems and solutions, (2) engage stakeholders, (3) develop an implementation research logic model, (4) help implement relevant interventions as needed for a given project, (5) monitor data, and (6) perform ongoing reflection and evaluation. Through this process, we will develop best practices in policy evaluation (Figure 2). The theoretical frameworks guiding our project evaluations include the Consolidated Framework for Implementation Research (CFIR) and the Reach, Effectiveness, Adoption, Implementation, Maintenance (RE-AIM). CFIR organizes factors that influence implementation outcomes across 5 domains—intervention, outer setting, inner setting, individuals, and implementation process [42]. The RE-AIM framework focuses explicitly on issues and steps in the implementation process that may improve or impede desired impact [43,44]. The principles of Lean Six Sigma and the Learning Health Systems Knowledge to Action Framework ensure a continual feedback loop of lessons learned [45].

### Ethical Considerations

This policy center was funded by a QUERI quality improvement center award mechanism and as such, the center and projects are to be conducted as quality improvement nonresearch. In alignment with regulatory guidelines, this center application was reviewed by the VA Salt Lake City Human Research Protection Office and received approval as quality improvement. It is notable that QUERI projects and centers are funded as quality improvement; research-regulated projects are funded by other services within the Veterans Health Administration, Office of Research and Development. Participants’ identities are kept confidential, and data are stored behind VA fire walls. All methods are performed in accordance with relevant guidelines and regulations. Informed consents, compensation for participants, and institutional review board oversight are not applicable for quality improvement projects funded as part of SALIENT.

Consent has been provided by coinvestigators and project leads to use names in text, tables, and figures within this protocol.

### Overarching Processes: SALIENT Core Integration

After PEPReC assigns evaluations [46], the Operations Core (Lead Cochran) will coordinate work using Lean methodologies in addition to managing contracts and external reporting (eg, QUERI, PEPReC, other VA stakeholders, and the Office of Management and Budget). The Methods Core (Leads Vanneman and Zickmund) will ensure that appropriate methodologies are used for each evaluation (objective 1). The Knowledge Translation (KT) Core (Lead Fagerlin) will use state-of-the-art translation of evaluation results into action and policy. KT Core team integration will enhance impact through guidance as evaluations are developed and implemented and products such as executive summaries and playbooks are produced (objective 2). All cores will prioritize the development of best practices in evaluation science (objective 3) and training of diverse implementation scientists (objective 4).

### Objective 1: Develop and Conduct Evaluations

#### Overview

As described in “Lean Sprint and Lean Six Sigma in Evaluation Processes” section, SALIENT begins each evaluation with a Lean Sprint to scope the evaluation topic, articulate evaluation objectives, develop evaluation questions, match methods to the questions, and identify possible pitfalls and potential contingency plans required to ensure high-quality evaluations. The mission of the Methods Core is to support and conduct evaluations by ensuring that appropriate, rigorous methods are used to address all assigned evaluations. SALIENT proposes a Methods Core with extensive expertise to apply qualitative, quantitative, and mixed methods to assigned evaluations. Multimedia Appendix 3 identifies investigators and leads affiliated with each methodological focus.

#### Lean Sprint and Lean Six Sigma in Evaluation Processes

We used Lean Sprint and Lean Six framework process to successfully complete an extensive evaluation of post-9/11 women veterans unemployment in 9 months (Table 2) and will continue this model integrating Lean methods within our implementation framework.

Using our established expertise, SALIENT will use Lean Sprint (iterative, incremental, time-boxed iteration cycle for sourcing, ranking, and testing new ideas) [47] and Lean Six Sigma (hereafter Lean Six) processes to conduct evaluations and inform continuous development for each evaluation and the center as a whole. Lean Six includes 5 operational stages (Define, Measure, Analyze, Improve, and Control) to manage the operations of each evaluation. Lean Six processes are management strategies for process improvement that have been applied in industry sectors including health care [48] for improvement of management processes and outcomes [49]. Evidence also shows the value of using Lean Six processes for planning [38] and producing improvements for clinical and translational research center workflow [50]. Because VA continues to train operations and medical center staff in Lean Six principles, this will facilitate alignment with operational partners and subsequent translation and implementation of policies and programs on completion of each evaluation.

On evaluation assignment, SALIENT MPIs will identify an evaluation team (ET) lead based on requirements of the evaluation and expertise to complete the evaluation. The ET lead will conduct a Pre-Sprint meeting with MPIs, core leads, operational partners, and other stakeholders to identify Lean Sprint needs and objectives, identify the ET investigators and staff, and assemble other key stakeholders and participants for the Lean Sprint team based on the evaluation content, appropriate methods, policy implications, and relationship to the phase of the QUERI Implementation Roadmap.

**Table 2:** Timeline of completed evaluation of women veterans’ unemployment.

Lean Six method	Evaluation task	Months											
		1	2	3	4	5	6	7	8	9	10	11	12
Define	Lean sprint	✓											
Measure	Data collection		✓	✓	✓	✓	✓	✓					
Analyze	Analysis					✓	✓	✓	✓				
Improve	After-action evaluation									✓	✓		
Control	After-action integration											✓	✓

#### We Will Use Lean Sprint to Develop the Evaluation Plan (Define)

**Figure 3:**
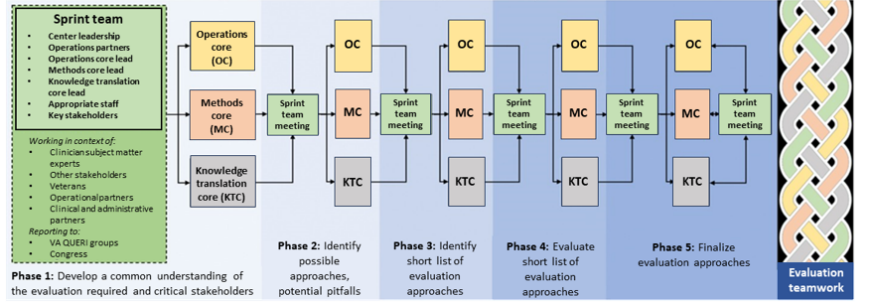
Use of Lean Sprint and Lean Six methodology to develop implementation evaluation plans. KTC: Knowledge Translation Core; MC: Methods Core; OC: Operations Core; QUERI: Quality Enhancement Research Initiative; VA: Department of Veterans Affairs.

The Operations Core will facilitate the Lean Sprint, with engagement from MPIs, ET, and core leads. The Lean Sprint team will meet weekly for ~5 weeks, with individual core teams meeting between to inform decision-making (Figure 3). The goals of each phase are as follows. In phase 1, the team will develop a common understanding of the evaluation required and evaluation questions to be addressed and identify possible approaches for each question, potential pitfalls, and other critical stakeholders and team members for the evaluation. In phase 2, the team will evaluate options (pros, cons, and pitfalls) of approaches and identify additional team members. In phase 3, the team will create a short list of evaluation approaches. In phase 4, the team will evaluate options on the short list. In phase 5, the team will finalize the evaluation plan (Figure 3).

#### Complete Evaluation Plan Using Lean Six Management Model

The cross-core collaborations in the 5 operational stages of Lean evaluation operations [50,51] are depicted by the braid on the right side of Figure 3. This symbolizes the close interaction among cores in biweekly ET meetings throughout the evaluation as they measure (conduct analysis), analyze (synthesize results for reports), improve (after action evaluation) and control (develop improved processes for future evaluations) stages. Throughout evaluations, the Operations Core will facilitate communication with and between the Methods and KT Cores including milestones and timelines to monitor performance of evaluations. The Operations Core will provide feedback to the ET and core leads on timelines, needs, and actionable performance monitoring to ensure that ETs meet contractual and stakeholder expectations. Evaluation facilitators will be assigned to an ET as it is formed and provide substantive support and guide research analysts assigned to each evaluation. Dr Cochran (Operations Core Lead with Greenbelt certification in Lean Six Sigma) will maintain communication using short huddles (1-2 times per week or as needed) with SALIENT leadership.

### Objective 2: Develop and Deploy Knowledge Translation Resources

#### Overview

The mission of the KT Core is to translate findings produced by SALIENT into useful and informative formats for stakeholders and policy makers and to inform best practices in policy evaluation and implementation. Led by Drs Fagerlin and MPI Haun, the KT Core will identify items essential to any knowledge translation process: (1) Stakeholders—priorities, capabilities, strengths, weaknesses, and incentives; (2) Actions—potential policy changes and operational changes; and (3) Outcomes—help VA achieve better health outcomes for veterans. SALIENT investigators have expertise in knowledge translation at the patient, provider, and system levels; development of plain language patient and clinical educational tools; and training postdoctoral fellows. The coleads of the KT Core will leverage Dr Haun’s experience integrating key stakeholders in the evaluation process and Drs Haun’s and Fagerlin’s experience in communication of results. Together they will support stakeholder engagement in projects and lead knowledge dissemination.

#### KT Approach

**Figure 4:**
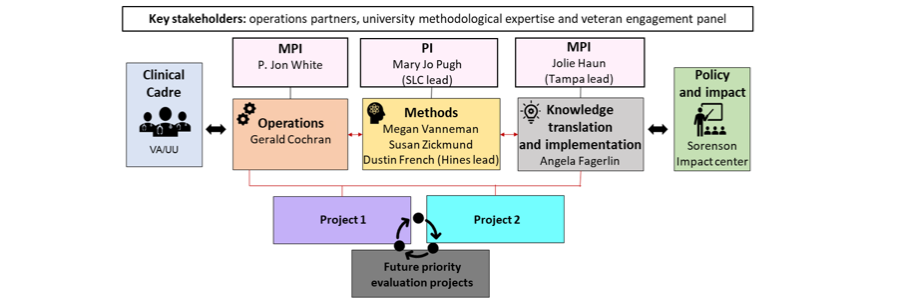
Structure of StrAtegic PoLicy EvIdence-Based Evaluation CeNTer (SALIENT) team. Projects and leads were subject to change upon the start of SALIENT funding. MPI: multiple principal investigator; PI: principal investigator; UU: The University of Utah; VA: Department of Veterans Affairs.

The KT Core will be involved in all phases of the evaluation (Figures 3 and 4).

As the evaluation plan is developed, the KT Core will begin development of key components of a playbook based on the QUERI Implementation Roadmap and ACTION Impact Framework [40,41]. For each evaluation, playbook development will carry out and adapt 4 steps to determine stakeholders, actions, and outcomes; create dissemination tools; disseminate findings; and evaluate dissemination outcomes. Multimedia Appendix 4 gives an example of a playbook overview developed for another QUERI project (MPIs: Haun and Pugh).

#### Determine Key Stakeholders, Actions, and Outcomes

The KT Core will provide an initial list of key stakeholders who would implement or be impacted by evaluation findings (Pre-Sprint) by (1) systematic review of VA operational partners and VA leadership, (2) Veteran Engagement Panel review, and (3) discussion with relevant VA and non-VA experts. This list will be finalized during the Sprint. We will create a table defining the priorities, capabilities, strengths, weaknesses, and incentives of each stakeholder group (eg, VA leaders, clinicians, veterans) and the role of each stakeholder for the specific evaluation. We will work with stakeholders to create feedback channels, report progress regularly, adjust strategy, and make data accessible.

#### Create Dissemination Tools

Throughout evaluations, the KT Core will collaborate with project teams to create dissemination tools tailored to each stakeholder and their priorities, capabilities, strengths, weaknesses, and incentives with feedback from Veteran Engagement Panels (Veterans Administration Salt Lake City and others), evaluation Clinical Cadre, and Declarative. Declarative will help identify communications strategies across stakeholder groups, business case analyses, and implementation plans. For each stakeholder, we will develop a formal briefing document, infographics, a “talking points memo,” and a list of suggested resources and methods to use findings to make changes to VA priorities, regulations, processes, budgets, and (as appropriate) national law. The briefing document will describe the problem, the outcome of the evaluation including possible impact of outcomes on a federal level (as possible) (using “plain language” and figures to make outcomes simple to digest), stories of veterans affected, case studies, and suggested changes to VA due to outcomes (regulations, processes, and budgets). We will create tailored infographics [52] and design each dissemination tool considering (1) key information for each stakeholder, (2) key points to be emphasized that will illustrate outcomes and benefits of implementing strategies, and (3) how each tool should be tailored for stakeholders [53].

#### Dissemination of Findings

The KT Core will facilitate communication for 2 types of stakeholders. General operations will inform VA leadership, VA operational partners, QUERI, PEPReC, and other QUERI programs about SALIENT expertise, evaluations, and staff development and enrichment. Content-specific partners include those described in the section titled “Determine Key Stakeholders, Actions, and Outcomes.” The KT Core will respond to knowledge translation inquiries from operations partners, policy makers, and the broader research community. Specific outputs and evaluation of specific approaches for each type of stakeholder may include a SALIENT web page, a VA-approved YouTube channel, monthly SALIENT leadership teleconferences with stakeholders, distribution of tailored dissemination tools, peer-reviewed publications, and conference presentations. Topics covered will include SALIENT team expertise and evaluations, training on priority topics, and alignment with QUERI and VA priorities.

#### Evaluation of Dissemination

In the evaluation sustainment phase, we will evaluate whether our results had an impact on veterans’ health and well-being and the VA (eg, impact on regulations, processes, budgets, and laws). Annually, we will examine relevant VA processes, regulations, laws, and budgets to determine whether they reflect findings from our evaluation and whether our findings and dissemination tools are described. As part of the playbook, each evaluation will receive a scorecard that will include a short list of high-priority policy or programmatic goals (3-5) to implement from the evaluation, operations partners, and QUERI Central Office. Scorecard goals, and additional impacts, will be aligned with the QUERI Action Impact Framework domains [41]. The playbooks and scorecards will be planned in the initial Lean Sprint. As an evaluation progresses and data are gathered and analyzed, analogous to the QUERI Implementation Framework [40], evidence-supported strategies, resources, interventions, and benchmarks that support solutions will evolve.

### Objective 3: Develop Best Practices in Policy Evaluation and Implementation

We propose a multilayered theoretical approach, using rigorous mixed methods that relies on implementation science frameworks, Lean Six processes and a strategic process across SALIENT project conceptualization, data collection, analysis, implementation strategy development, reporting, synthesis, and dissemination over time. RE-AIM, CFIR, evidence-based implementation strategies, and the Implementation Research Logic Model process will inform our conceptual lens across projects to standardize our approach to policy evaluation and implementation over time.

Consistent with Lean Six [48] the Operations Core is charged with continuous and sustained improvement of center operations to ensure that performance is meeting stakeholders’ expectations and the SALIENT team improves work processes and optimizes efficiencies during and after each evaluation. The Operations Core will facilitate the feedback loop with stakeholders, cores, and ETs using periodic reflections [54], throughout evaluations, allowing rapid feedback from stakeholders on aspects of design and execution that will inform the direction of the project, modifications, and development of a learning health system (Analyze and Improve phases). We will facilitate a continuous feedback loop to avoid misalignment in goals and expectations, allow rapid and strategic course corrections throughout the evaluation, and ensure that the end user can optimize use of evaluation results.

To develop best practices in policy evaluation and implementation across projects over time, we will develop a SALIENT Policy Evaluation Catalog of projects to document project factors (ie, policy type, stakeholders, constructs, implementation strategies, communication and dissemination strategies, and mechanisms). We also propose to collaborate with other centers to collect these data on their evaluations to inform large-scale synthesis of policy evaluation and implementation best practices. Data from our inventory can be used by SALIENT, other centers, and the coordinating center for cross-project observations and learning, which is a broader opportunity for overall QUERI development. To accomplish this, the Operations Core will conduct after-action evaluations including operations partners (see left side of Figure 3), investigators, and end users of the evaluation to discuss successes or concerns observed during the evaluation documented by detailed field notes and recordings. A quantitative postevaluation assessment (Multimedia Appendix 5) will capture operations successes and barriers.

Using the SALIENT Evaluation Policy Catalog data we will brainstorm alternatives, overcome barriers experienced, and inform future evaluation development and processes. These data will be used to create Lean Six tools, such as Value Stream Maps (schematics used to identify activities that have value to stakeholders as well as waste, delays, and inefficacies), to identify strengths and weaknesses of SALIENT activities. Examples of wastes, delays, or inefficiencies could include team members from one core unnecessarily waiting for completion of entire components of evaluations (batches) instead of working continually as smaller components are completed (single-piece workflow); a highly inefficient approach to collaborative work processes. These schematics and proposed priorities will be discussed with center and core leads. The group will come to consensus on priorities and their rank order. Sustaining improvements are ensured by this type of regular review and use of audit checklists for continued monitoring. These analyses will synergize findings and processes across cores and evaluations to develop best practices in policy evaluation and implementation.

### Objective 4: Train Diverse Evaluation and Implementation Scientists

#### Overview

We aim to recruit, train, and retain highly skilled investigators in evaluation and implementation science. We will recruit “SALIENT Scholars” to participate in the Advancing Diversity in Implementation Leadership (ADIL) program in addition to graduate students and postdoctoral fellows who can grow our ADIL program. We will leverage our suite of advanced fellowships to provide a cohort and peer support for ADIL participants. We will also leverage The University of Utah (UU) Vice President’s Clinical and Translational Research Scholars Program for junior investigators, career development, and leadership training (eg, weekly seminars and a 3-day career development retreat) led by the director of career and leadership training at UU and early career coaching designed specifically for underrepresented scientists. These targeted programs will assist ADIL participants in identifying additional career and near-peer mentors who are also scientists who identify as underrepresented in science. Our team includes such junior investigators (Naranjo).

As we do in other fellowships, we will use a Matrix Mentoring Model [55] where each fellow has a content mentor, a senior scientific mentor, and a KT mentor. Mentees will meet with MPIs and core leads to establish mentoring teams and complete career development plans (CDPs). Mentees will meet twice monthly with their scientific mentors and twice per year with mentor teams to review productivity, plan for the next 6 months, and track progress toward program and career goals. Mentees will write and submit annual progress reports to the MPIs, who will review them with the mentees. Mentees will also follow a personalized CDP while working on evaluations guided by their mentoring team. The mentoring program will parallel that of the Combatting Antimicrobial Resistance through Rapid Implementation of Available Guidelines & Evidence QUERI, which includes didactic training, group learning, leadership development, external engagement (eg, Center for Evaluation and Implementation Resources, for consultation, instruction, and guidance related to their project, participation in the Implementation Research Group, a national learning collaborative), dissemination support, and tracking of performance considering person goals and CDPs. Dr Rubin, lead of the Combatting Antimicrobial Resistance through Rapid Implementation of Available Guidelines & Evidence QUERI mentoring core and the UU Vice President’s Clinical and Translational program, and Dr Knight (co–primary investigators of Informatics, Decision Enhancement, & Analytics Sciences HSR fellowship) and Dr Fagerlin (PI of UU TL1 pre- and postdoctoral training programs) will integrate SALIENT Scholars (and SALIENT mentors) into their existing training programs. In addition to these fellowships, SALIENT will include training in Lean Six methods using existing VA training mechanisms and training with Operations and KT Core faculty. Training will also be offered to other fellows and trainees (eg, graduate students, faculty) from partnering programs and sites.

#### Center Management Plan

**Figure 5:**
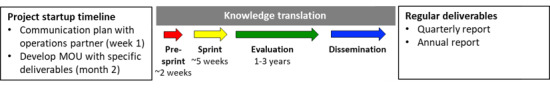
Evaluation timeline and deliverables. MOU: memorandum of understanding.

Upon proposal resubmission to address reviewer comments (Multimedia Appendices 6 and 7) and funding acceptance, the MPIs, site leads, core leads, and investigators attended a dual digital and in-person hybrid kickoff meeting in December 2022. At this kickoff, we attended to all preliminary start-up issues and collaborated to initiate projects and core efforts. The kickoff provided the setting for connecting all 3 sites, setting the standard and logistics, to continue their effective multisite communication. After the kickoff meeting, we initiated team’s meetings and shared project folders and other relevant logistics for communication and collaboration. Second, the entire SALIENT team attends quarterly meetings with operational and veteran stakeholders on projects to address evaluation activities, benchmarks, project issues, and ultimately subsequent implementation efforts. This ongoing communication facilitates changes or operations-prompted amendments to the projects, as sometimes occurs. In this case, the MPIs consult with operations and reconceptualize project activities and deliverables as needed. Third, we conduct digital biweekly meetings with the MPIs and project teams during which each team will report on progress, roadblocks, and findings. Fourth, we conduct digital project-specific weekly meetings, attended by the MPIs, leads, coinvestigators, and project managers to address day-to-day activities. Fifth, the MPIs and site and project leads formulate email updates to send to all SALIENT members—updates will include project progress, training opportunities, and other content relevant to other ongoing meetings. These updates are summarized in quarterly reports to operational partners. Sixth, team members communicate as needed via email, phone, and videoconference as done during the development of this proposal. Given the long-standing collaboration among the MPIs and site leads, and our current digital collaboration practices, we do not anticipate any concerns. SALIENT sites will use 6 primary means for center management and communication (Figure 5).

## Results

Funding and start-up activities for SALIENT began in October 2022. Current SALIENT evaluation projects are underway, and funding is expected to continue through September 2027. Anticipated project evaluation results will be disseminated as projects end on their projected timelines. Reporting will be performed at funding, operational, and congressional levels as appropriate. SALIENT KT Core efforts will support rapid dissemination to key partners and organizations.

## Discussion

### Principal Considerations

The goal of this evaluation center is to support the successful identification and implementation of VA high-priority operational evaluation projects that are aligned with VA population and system needs. This protocol illustrates a multilevel theoretical and Lean Sprint methodology to conduct rapid rigorous operations-based policy evaluation in a learning health system. To our knowledge, this protocol is unique in that it informs a center-wide Lean Sprint methodology to conducting quality improvement to evaluate priority policies. Our impact will be to accelerate the generation, dissemination, and implementation of evidence-based policy recommendations pursuant to the Evidence Act, develop best practices in policy evaluation and implementation, and train diverse evaluation and implementation scientists.

### Strengths and Limitations

This center protocol contributes to the field in four distinct ways by (1) using a Lean Sprint methodology (iterative, incremental, rule-governed approach to clearly defined, and time-boxed work) to develop our evaluation plans collaboratively with operational partners and key stakeholders including veterans, policy experts, and clinicians; (2) using a multicore approach (operations, methods, and knowledge translation) to conduct evaluations and rapidly disseminate findings to inform knowledge translation; (3) developing a center infrastructure to train diverse evaluation scientists to democratize evaluation, implementation, and knowledge translation expertise; and (4) identifying best practices in policy evaluation and implementation to inform learning health systems.

Potential weaknesses of the center should also be considered. First, given geographic diversity, communication breakdowns across sites are a risk; however, a comprehensive center management plan and digital communication methods ensure continuity in communication and center-site interactions. Second, given the size of the center and rapid nature of projects, there is a risk of diffuse efforts or mismanagement of resources; however, the multicore approach of the center ensures infrastructural, administrative, and resource support from an administrative, operational, and methodological perspective. Third, with shifting topical and funding priorities, continuity in resources and staffing could pose challenges across projects over time; however, SALIENT leadership is engaged with QUERI, other QUERI Evidence Act evaluation centers, and operational leadership to anticipate and respond to the changing climate to allow for symbiotic reciprocity. Furthermore, SALIENT’s Lean Sprint systems redesign approach maximizes resources and sustained efficiency of projects. Fourth, often operations-based projects require data sets and resources that may not be readily available, particularly on the rapid timeline of high-priority projects. SALIENT subject matter experts have a highly developed network of resources and collaborations within and beyond VA to ensure currency and continuity across data resources.

### Conclusions

In summary, the SALIENT environment has exceptional evaluation and training resources; has established infrastructure with a proven track record of rapid evaluation and dissemination of results; and strong, lasting operational partnerships and collaborations. The SALIENT team is poised to rapidly conduct state-of-the-science evaluations to support the broad VA response to the Evidence Act and high-priority legislative requirements related to environmental hazards and airborne exposures. SALIENT evaluations will contribute to (1) optimized policies and programs for veterans; (2) improved outcomes regarding health, equity, cost, and provider well-being; (3) advances in the science of policy evaluation and knowledge translation by synthesizing learning across SALIENT and other center evaluations; and (4) expansion of the implementation and dissemination science workforce.

## Appendix

Multimedia Appendix 1 Department of Veterans Affairs funding opportunity announcement request for applications. VA: Department of Veterans Affairs, VA-ORD: Office of Research and Development; QUERI: Quality Enhancement Research Initiative; RFA: Request for Applications.

Multimedia Appendix 2 Data resources compiled by StrAtegic PoLicy EvIdence-Based Evaluation CeNTer (SALIENT) investigators to be used in future evaluations and scientific investigation.

Multimedia Appendix 3 StrAtegic PoLicy EvIdence-Based Evaluation CeNTer (SALIENT) team methodological expertise with selected references.

Multimedia Appendix 4 Playbook overview developed for another Quality Enhancement Research Initiative project (multiple principal investigators: Haun and Pugh).

Multimedia Appendix 5 Quantitative post-project team evaluation template.

Multimedia Appendix 6 Request for applications reviewer comments.

Multimedia Appendix 7 Request for applications resubmission responses to reviewer comments.

## Abbreviations

ACTION: Alignment with multilevel priorities, Commitment from operational partners, Tailoring to the local context, Informing the field, Observing health care changes, and generating New questions and projects
ADIL: Advancing Diversity in Implementation Leadership Program
CDP: career development plan
CFIR: Consolidated Framework for Implementation Research
ET: evaluation team
HSR: Health Systems Research
KT: Knowledge Translation
MPI: multiple principal investigator
PEPReC: Partnered Evidence-Based Policy Resource Center
QUERI: Quality Enhancement Research Initiative
RE-AIM: Reach, Effectiveness, Adoption, Implementation, Maintenance
SALIENT: StrAtegic PoLicy EvIdence-Based Evaluation CeNTer
UU: The University of Utah
VA: Department of Veterans Affairs

## Data Availability

Data for center projects are available from the authors upon reasonable request. Requests can be made to the corresponding author for referral to appropriate SALIENT project lead.

**Disclaimer**

The corresponding author, as a US government employee, has the right to grant on behalf of all authors and does grant on behalf of all authors a nonexclusive worldwide license to the publishers and its licensees in perpetuity in all forms, formats, and media (whether known now or created in the future) to (1) publish, reproduce, distribute, display, and store the contribution, (2) translate the contribution into other languages; create adaptations and reprints; include within collections and create summaries, extracts, and abstracts of the contribution, (3) create any other derivative work(s) based on the contribution, (4) exploit all subsidiary rights in the contribution, (5) include electronic links from the contribution to third-party material wherever it may be located, and (6) licensee any third party to do any or all of the above.
