# Supplementary material for: Developing Evidence to Support Policy: Protocol for the StrAtegic PoLicy EvIdence-Based Evaluation CeNTer (SALIENT)
Source: JMIR Res Protoc. 2024 Sep 19;13:e59830. doi: 10.2196/59830 (PMC11450355; doi:10.2196/59830)
Supplement: Multimedia Appendix 1 [file resprot_v13i1e59830_app1.pdf]

Part I - Overview Information

Department of Veterans Affairs

Funding Opportunity Announcement/Request for Applications (FOA/RFA)
HX-22-026

Title
QUERI Evidence-based Policy Evaluation Center Award

Participating Service
Quality Enhancement Research Initiative (QUERI)
Veterans Health Administration, Office of Research and Development (VA-ORD)

Announcement Type
New

Catalog of Federal Domestic Assistance Number
64.054

Competition Identification Number
HX-22-026

Note about COVID-19:
With the emergence of national priorities, such as the COVID-19 pandemic, applicants should ensure that their implementation and evaluation strategies use virtual care, electronic, and other non-face-to-face encounters wherever possible. For example, many evidence-based practices (EBPs) and implementation strategies can be adapted for delivery over telephone, video, or other virtual formats. Data collection should include options for electronic mail and phone-based interactions as well as electronic health record and e-health technologies that reduce respondent burden, where appropriate.

Summary of Important Updates: Important items and changes are highlighted in yellow throughout the FOA/RFA.

Applications submitted in response to this FOA/RFA must be submitted using the VA-ORD Application Guide SF424 Research and Related (R&R) forms and forms available on the VA-ORD Intranet site at http://vaww.research.va.gov/funding/electronic-submission.cfm.

NOTE: The instructions in this FOA/RFA may differ from, and supersede, the general instructions contained in the VA-SF424 AG.

See fatal errors section for the list of errors that will result in an administratively withdrawn application.

Key Dates*
RFA Release/Posted Date: April 1 (Summer) or October 1 (Winter)
Intent to Submit Receipt Date(s): April 1 to May 13 (Summer) or October 1 to November 14 (Winter)
Application Deadlines, Submission, Peer Review, and Start Dates: See Table 5.

**If the date falls on a weekend or Federal holiday, the deadline is the next business day.*

Note: Dates are subject to change.

Table of Contents

Part I Overview Information

Part II Full Text of Announcement

Section I. Funding Opportunity Description
1. Executive Summary
2. Research Objectives

Section II. Award Information
1. Mechanism of Support
2. Application Types Allowed
3. Multiple Awards and Submissions
4. Funds Available
5. Location of Research Space
6. Duplicate Submissions

Section III. Eligibility Information
1. Eligible Institutions
2. Eligible Individuals

Section IV. Application and Submission Information
1. Intent to Submit
2. Request Application Information
3. Content and Form of Application Submission
4. Submission Timelines and Processing Information
   A. Deadline, Review, and Award Dates
   B. Application Processing
5. Intergovernmental Review
6. Funding Restrictions
7. Other Submission Requirements

Section V. Application Review Information
1. Criteria
2. Review and Selection Process
   A. Additional Review Considerations
   B. Sharing Research Data
   C. Sharing Research Resources

Section VI. Award Administration Information
1. Award Notices
2. Administrative and National Policy Requirements

Section VII. Agency Contacts
1. Scientific/Research Contact
2. Financial Management Contact

Part II - Full Text of Announcement

Section I. Funding Opportunity Description

1. Executive Summary

This Funding Opportunity Announcement (FOA)/Request for Applications (RFA) will use the non-U.S. Department of Health & Human Services (HHS) Research Collaboration (I50) award mechanism.

Purpose. The Veterans Health Administration (VHA) Office of Research and Development (ORD) announces an opportunity for Department of Veterans Affairs (VA) medical facilities to compete for funding through the Quality Enhancement Research Initiative (QUERI) to support Centers that conduct rigorous, independent evaluations focused on national priorities that are informed by the Foundations for Evidence-based Policymaking Act (Evidence Act., US PL 115-435) of 2018.

Veteran-Centered Mission. The mission of the VA Quality Enhancement Research Initiative (QUERI) is to improve Veteran health by accelerating the adoption of evidence-based practices (EBPs) in routine care settings. For more than 20 years, QUERI has led a national network of centers devoted to identifying best practices, implementing them in clinical settings, and rigorously evaluating the results of these efforts to inform VA policy and practice. Since 2015, QUERI has led national efforts in VA to conduct program and policy evaluations to support legislative mandates, including the Veterans Access, Choice, and Accountability Act of 2014 (Veterans Choice Act, US PL 113-146), the VA Maintaining Internal Systems and Strengthening Integrated Outside Networks (MISSION) Act, and most recently, the Foundations for Evidence-based Policymaking Act of 2018 (Evidence Act, US Public Law 115-435), which requires that agency budgets be supported by evidence and evaluation (Hahn et al, 2019). QUERI is also responsive to Executive Branch priorities, such as the White House Memorandum on Restoring Scientific Integrity, notably by ensuring successful implementation of programs and policies supported by evidence through evaluation and learning.

QUERI's strategic methodology supporting evidence-based policy goals is threefold:

- Implement and sustain EBPs and promising innovations across various health settings, especially in settings with demonstrated gaps in quality or outcomes.
- Conduct rigorous evaluations in partnership with national and regional health system leaders to optimize programs and policies affecting Veterans across different settings.
- Promote the application of implementation, evaluation and quality improvement science to ensure that national programs and policies are informed by and benefit from real-world practice.

QUERI's 2021-2025 Strategic Plan and accompanying strategic methodology are aligned with the Office of Research and Development (ORD)'s priority of increasing the substantial real-world impact of research by accelerating the implementation and sustainment of evidence-based practices into clinical and population-based settings. These goals are also aligned with emerging VHA priorities related to the promotion of a learning organization through knowledge translation and the VA Strategic Plan for FY2022-2028, which highlights the need for VA to deliver timely, accessible, high-quality care and services to meet the unique needs of Veterans and all eligible beneficiaries. QUERI strategic goals are responsive to recent VA and VHA executive and legislative priorities that fulfill the requirements of the Evidence Act, and VHA's ability to address the Government Accountability Office's (GAO) high-risk list determinations, as referenced by the Office of Management and Budget's (OMB) FY23 budget planning guidance. Since 2016, QUERI has developed 200+ products to support implementation of effective practices, conducted over 45 national evaluations, and trained over 26,000 VA employees in EBPs that have benefited hundreds of thousands of Veterans, as cited by a recent Government Accountability Office report on translation of research into practice.

Evidence-based Policy Evaluation. As underscored by the White House Memorandum on Restoring Scientific Integrity and the Evidence Act, evidence-based policymaking involves making decisions that are based on or informed by rigorously established objective evidence and is paramount for ensuring programs and policies are effective for those who need them the most. OMB in Memorandum M-21-27, defines

evaluation broadly to include an "assessment using systematic data collection and analysis of one or more programs, policies, and organizations intended to assess their effectiveness and efficiency."

QUERI national evaluations consider questions related to implementation and sustainment as well as effectiveness across different populations and settings to inform strategies and ensure effective programs and policies are sustainable at the clinic level. Currently, QUERI supports evaluations of specific national policies and programs (e.g., Whole Health, EHR Modernization, High-reliability Organization implementation, suicide prevention, opioid/pain treatment, long-term care) through its Partnered Evaluation Initiative (PEI) mechanism and enterprise-wide VA initiatives through the Partnered Evidence-based Policy Resource Center (PEPReC).

OMB is increasing its enforcement of the Evidence Act, notably through required annual evaluation plans that are derived from VA-wide strategic plans and inform budget decisions. QUERI is reviewing VHA national program office legislative and budget requests for evidence and need for evaluation. In addition, QUERI has increasingly responded to national VA evaluation requests arising from legislation and executive branch priorities, especially related to vulnerable, underserved, and at-risk Veteran populations. As a result, there is increased demand for national evaluations to inform VA and VHA programs and policies.

Evidence Act and QUERI Evaluation Process. The Foundations for Evidence-Based Policymaking Act of 2018 (also known as the Evidence Act, PL 115-435) updated the 2010 Government Performance and Results Act and the 1996 Clinger-Cohen Act and requires all Cabinet-level agencies, including VA, to ensure budgets are tied to, supported by, and justified by evidence and evaluation. Since 2019, per the direction of the VHA Under Secretary for Health and VA Office of Enterprise Integration, QUERI has superintended efforts in VA and VHA to help the agency fulfill core Evidence Act requirements. As interpreted by the Office of Management and Budget (OMB), which is responsible for promulgating formal guidance and overseeing the implementation of the law, Evidence Act core requirements include a quadrennial Learning Agenda tied to the agency's strategy plan, quadrennial capacity assessments of evidence-building and evaluation activities, and annual evaluation plans, which are made public yearly. The Capacity Assessment is used to assess the ability and bandwidth of programs and offices to perform evaluation activities. The Learning Agenda provides an overview of specific policy questions tied to the VA FY2022 – FY2028 Strategic Plan, and the Annual Evaluation Plan details evaluation activities that address the policy questions posed in the Learning Agenda. The FY22 Annual Evaluation Plan and FY23 Annual Evaluation Plan are available online. Informally, as it is not required by law, a strength of evidence checklist, developed by PEPReC, is used to assess how budget and legislative proposals are justified by evidence. Details on how VHA has implemented the Evidence Act are provided in a policy brief produced by PEPReC.

At the direction of the VHA Under Secretary for Health and VA Office of Enterprise Integration, QUERI applies its Evidence-based Policymaking Lifecycle to help VA and VHA fulfill Evidence Act goals. Endorsed by the VHA Governance Board and operationalized through the Evidence-based Policy Subcommittee of the VHA Strategic Directions Committee, the Lifecycle seeks to enhance VA and VHA's capacity as a Learning Health System by planning, implementing, and communicating evidence and evaluation to inform programs and policies for Veterans (Figure 1). Annually, VA (through QUERI) issues a call for significant health care priorities that address VHA and VA policy goals towards improving Veteran outcomes. Specifically, QUERI identifies Evidence Act and other national program and policy priorities that it can address through its strategic methodology based on multiple sources of input from interested parties (e.g., VA and VHA national and VISN leadership, Congress, Veterans Service Organizations, scientific community, and others). QUERI may also identify emerging national priorities based on recent legislation, executive orders, or other Congressional mandates. Applications responding to priorities then undergo standardized peer review processes to ensure rigor and integrity of findings. Results from QUERI-funded evaluations are expected to be published in scientific peer-reviewed journals and communicated to interested parties within VA and other organizations. In addition to QUERI Evidence-based Policy Evaluation Center evaluations, other evaluation and implementation initiatives identified for potential QUERI support may undergo additional review for inclusion as Evidence Act evaluation plans by OMB and are subject to additional VA criteria for significance, established by the VA-wide Foundations for Evidence-based Policy Workgroup (FEBPWG). A more detailed overview of QUERI's priority setting process and activities supporting VA's implementation of the Evidence Act is provided here.

Figure 1. Lifecycle to Identify and Implement National VA Priorities

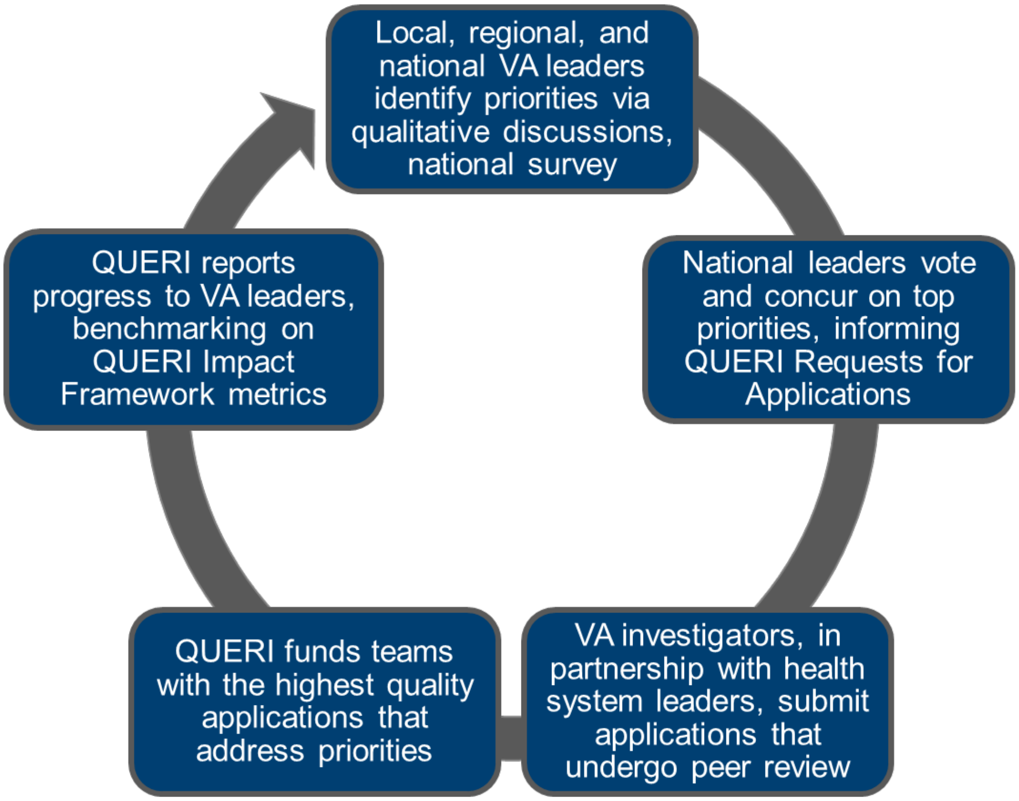

During FY21-22, VA leadership identified evaluation priorities for QUERI Evidence-based Policy Evaluation Centers.

Recently-funded QUERI Evidence-based Policy Evaluation Centers cover the following priorities:

1. Support EHR modernization, including effective quality improvement/change management strategies
2. Assess women's health initiatives (e.g., reproductive health, prosthetics)
3. Enhance the Veteran experience and quality of virtual care options in VA
4. Deploy strategies to address health disparities and Veteran social determinants of health
5. Reduce burnout and improve VA employee and trainee experience and outcomes
6. Evaluate the effectiveness and implementation of programs to eliminate homelessness among Veterans

Current priorities, based on multi-level input from diverse groups, that are not currently covered by existing evaluation centers are listed below. Evidence-based Policy Evaluation Center applications addressing the following priorities will receive priority funding consideration.

1. Improve long term care, aging in place, geriatric care, and home care service options
2. Evaluate strategies to mitigate the long-term impact of COVID-19, including reducing adverse outcomes associated with delayed or suppressed care
3. Assess and improve the quality and cost of community care, including enhancing community care and virtual care coordination to improve Veteran health
4. Assess MISSION Act standards of care and impacts on quality improvement and policy changes
5. Optimize integration of care for primary, mental health, specialty, and urgent care services
6. Improve clinical care efficiency (e.g., administrative, technology, supply chains)
7. Evaluate the impacts of military environmental exposures on Veterans, including clinical, health services, and organizational policies

Example Evaluation that was selected for inclusion in the VA FY2022 Annual Evaluation Plan:
VA Strategic Plan Learning Agenda Question: "How can VA ensure that Veterans have access to timely care in their preferred setting?"

- Evaluation question 1: How do medical scribes affect clinic function and patient satisfaction?
- Evaluation question 2: How effective are the MISSION Act-based underserved scores and subsequent mitigation strategies in addressing underserved facilities?

See FY22 Annual Evaluation Plan and FY23 Annual Evaluation Plan for more information and for details on how evaluation plans are developed.

QUERI Implementation Strategies Key to Evidence-based Policy. One of QUERI's unique contributions to evidence-based policymaking is its establishment, deployment, and evaluation of implementation or quality improvement (QI) strategies, which are highly-specified, theory-based methods designed to support the uptake of effective programs and policies and ensure their effectiveness the clinic level. Implementation strategies are essential to sustain effective programs and policies by ensuring their successful reach, effectiveness, adoption, implementation, and maintenance/sustainment (e.g., RE-AIM Framework) across different settings and Veteran populations. Derived from underlying frameworks, scientific literature, and most recently, the QUERI Implementation Roadmap (Figure 2), implementation strategies are crucial for supporting the VA's efforts to achieve and sustain core features common to Learning Health Systems and High-Reliability Organizations (HROs). Since 2016, QUERI centers have implemented over 55 EBPs nationally using implementation strategies.

Figure 2. Summary of the QUERI Implementation Roadmap

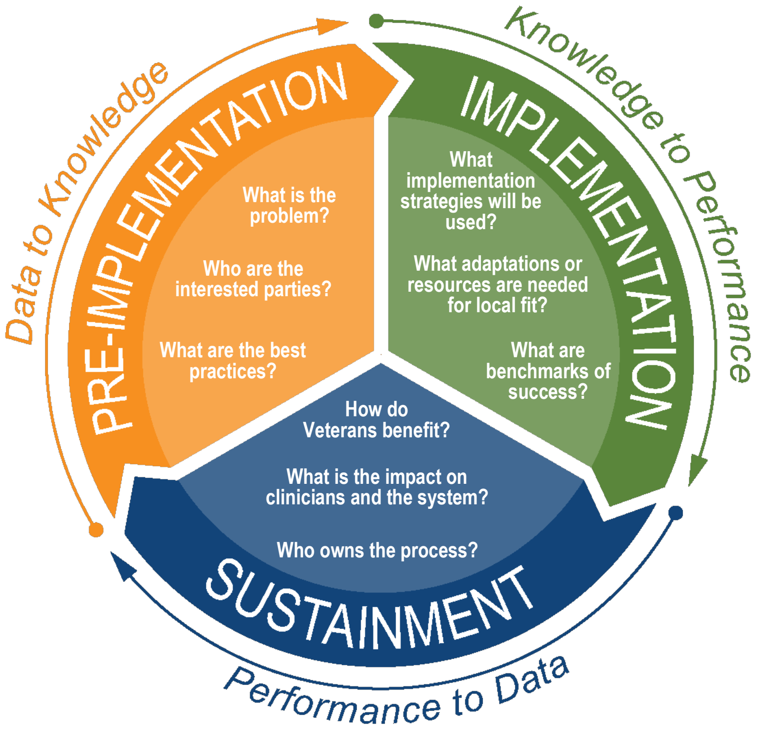

Multi-level Impacts. QUERI initiatives use several measures when assessing impacts of programs and policies at multiple levels (see QUERI ACTION Impact Framework), based on the Quintuple Aim (patient outcomes, health equity, population health, lower costs, provider well-being). Because regional and local operations leaders (e.g., facility or VISN leadership) are accountable for achieving national quality performance standards, QUERI outcomes should include VHA Network Director and Medical Center Director Performance Plan metrics, VHA Electronic Quality Measures), and other national quality measures, where appropriate and relevant to VA national and clinical operations priorities.

2. Objectives

This request for applications (RFA) is intended to support an infrastructure to conduct rigorous evaluations of programs or policies to inform improvements in Veteran care and outcomes. Because QUERI funds a number of evaluations in partnership with individual national program offices or VISNs, the intent of this RFA is to support ongoing capacity to respond to broader evaluation requests that are aligned with national priority goals through the Evidence Act and involve questions that transcend specific program offices.

Two types of evaluation centers are permitted:

A. **Evaluation Centers** conduct evaluations that are related to the Evidence Act Annual Evaluation Plans or are mandated by legislative priorities.

B. **Implementation and Evaluation Coordinating Center** supports the assignment and peer review of short-term evaluations to QUERI centers, training in evaluation, implementation practice, and quality improvement methods, and tracking of impacts of evaluations to meet Evidence Act goals.

Per OMB and for the purposes of this solicitation, Program Evaluation is defined as "assessment using systematic data collection and analysis of one or more programs, policies, and organizations intended to assess their effectiveness and efficiency." OMB recognizes that there are different approaches to evaluation, including Formative Evaluation, Impact Evaluation, and Process/Implementation Evaluation. Evaluations may address questions related to the effectiveness and implementation or institution of a program or policy, contextual and moderating/mediating factors that influence the success of a program or policy, potential variation in effectiveness/implementation across different groups or settings, and strategies that support sustainment of programs or policies over time. In all, these different dimensions of evaluation can provide critical information to inform decisions about current and future programming, policies, and organizational operations by addressing not only "does the program work?" but also "what makes it work?" and "what will it take to sustain the program across different populations and settings?"

Rigorous program and policy evaluations can produce more effective strategies to prevent waste and improve rollout, and ultimately produce greater return on the resources invested in implementing new programs. Pragmatic randomized trial designs, including cluster-randomized designs by which one can assess the effectiveness of a program or policy during and after implementation in comparison to a control group (usual care), are strongly encouraged. If randomization is not feasible, other designs, such as stepped wedge, are encouraged, and a comparison group must be available where appropriate. Key examples of randomized program evaluations in VA include: the Randomized Evaluation of the Caring Letters Campaign and Examining the effectiveness of an adaptive implementation intervention to improve uptake of the VA suicide risk identification strategy: a sequential multiple assignment randomized trial. Evaluations should include state-of-the-art mixed-methods data ascertainment incorporating perspectives and impacts across different interested groups, especially those who can help inform and optimize the implementation effort.

Applicants are encouraged to utilize the QUERI Implementation Roadmap, and consult the following HSR&D/QUERI resources for:

- Aligning evaluations with current national VA and Congressional priorities, including the Foundations for Evidence-based Policymaking Act: Partnered Evidence-based Policy Resource Center (PEPReC; peprec@va.gov).
- Conducting economic analyses and evaluation appropriate for the selected strategies and training on data science/organization: HSR&D's Health Economics Resource Center (HERC; herc@va.gov)
- VA data resources and considerations regarding Cerner implementation: HSR&D's VA Information Resource Center (VIReC; virec@va.gov)

A. Evaluation Center

Evidence Act Centered Activities. Evaluation Centers are expected to support the planning and directly conduct at least two evaluations per year. Evaluation topics are identified by VHA leadership annually (Figure 1) and are eligible for inclusion in VHA's Annual Evaluation Plans and Learning Agendas, as required by OMB. Evaluation Plans are expected to last 1-3 years depending on scope, and evaluations that extend beyond one year may include additional evaluation objectives or questions in subsequent years.

Evaluation Plan topics are selected and operationalized based on the QUERI Lifecycle described in Figure 1 and in Braganza et al (2022). Evaluation topics are initiated in several ways. Members of the VHA Evidence-based Policymaking Subcommittee, VHA/VA leadership, and QUERI Central Office leadership

may propose evaluation topics for review and approval by the entire Subcommittee. In some cases, Evaluation Plan topics will be based on emerging priorities identified from legislation, Executive Branch mandates or Executive Orders, the VA FY2022 – FY2028 Strategic Plan, and/or government-wide strategic plans or cross-agency priority goals. Topics approved for QUERI Evidence-based Policy Evaluation Center support must meet criteria specified in the VA FY2023 Evaluation Plan: existing lines of inquiry; mission focus on Veterans; care and services for at-risk, marginalized, underserved, and vulnerable Veterans; alignment with learning agenda and evaluation plans; and alignment with administration's existing prioritization process. Additional criteria include: timeliness of the evaluation topic, degree of evaluation independence, whether the topic requires multiple agency or Program Office input, and portfolio balance. Topics are then assigned by PEPReC to the Evidence-based Policy Evaluation Centers based on the Center areas of expertise and capacity. Centers will develop detailed Evaluation Plans, which will undergo peer review by PEPReC prior to approval by the Subcommittee and QUERI Central Office. Where appropriate, PEPReC may take on select evaluations focused on economics and policy.

Evaluation Centers will work closely with VA operations partners and policymakers, as their evaluations will focus heavily on VA policies and programs. Given the focus on VA/VHA fulfillment of Evidence Act evaluation requirements, Centers will be accountable to unique reporting and scheduling expectations, given the inclusion of its evaluations in VHA's response to OMB. Centers will liaise often with VA and VHA leadership and QUERI/PEPReC staff. Investigator teams with established relationships with operations partners are encouraged to apply.

Structure and Roles. Evaluation centers should include at least three cores and are expected to operate as an integrated, whole – coordinating across the cores and sharing resources, learnings, outcomes – in order to add value beyond the conduct of individual projects/activities (i.e., the whole is greater than the sum of the parts). Multi-site applications are encouraged but must demonstrate capability of operating as a single cohesive center, including work across the cores. Centers are strongly encouraged to incorporate innovative components (e.g., state-of-the-art evaluation, training, and knowledge translation methods, novel concepts or approaches) that have the potential to challenge and help shift current paradigms in evidence-based policymaking and drive impacts in how evaluation, QI, and implementation are done in VA.

Evaluation Centers will be independently operated and will coordinate and meet regularly with QUERI leadership, PEPReC staff, and VA and VHA leadership. A diverse evaluation team – in terms of skills, experiences, and backgrounds – is highly encouraged. Centers must include at least one investigator with quantitative data analysis expertise and one with qualitative/mixed-methods expertise. Applications from Centers with expertise in economic and budget impact analyses, quantitative analyses of large-scale datasets (e.g., large VA and non-VA data sources), policy analyses, implementation science, systems engineering, and clinical informatics (e.g., artificial intelligence and machine learning) are highly encouraged. It is strongly encouraged that Centers include investigators with interest and track records in diversity and inclusion.

Below are examples of potential Center staff roles:

- Senior investigator, i.e., Director(s): The senior investigators will oversee all core activities, evaluation activities, and associated personnel. They will provide leadership and project-specific guidance and will liaise between VA and VHA leadership, QUERI leadership, PEPReC, and Center staff.
- Investigators: Investigators will oversee day-to-day operations of the core activities and evaluations. They will oversee the policy and data analysis staff and offer guidance on evaluation progress, direction, and Evidence Act deliverables before submission.
- Policy analyst: The policy analyst will produce the annual Evidence Act deliverables, evaluation reports, external deliverables, and where applicable, implementation plans. This will require research, collaboration with other program offices and considerable internal teamwork.
- Data analyst(s): The data analysts will provide programming support for individual evaluations conducted by the Center. They will be on call for a variety of modeling needs and will also provide data analysis support to the field upon request.

- Program administrator: The program administrator will provide administrative support for all core activities and evaluation activities. The program administrator will be responsible for scheduling meetings, liaising between other offices, and finalizing any reports.
- Fellow(s): Fellows (Trainees) will partner with an investigator to design and execute a short-term evaluation plan, an extension of existing Evidence Act evaluations. They will produce actionable evidence while receiving training in evaluation best practices and management of implementation of program and policy evaluations.

The Evaluation Centers must include the following Cores:

1. The **Operations Core** is responsible for the overall administration of the Center. This Core designs and coordinates specific documentation requested on an annual basis by QUERI Central Office and other VA operations partners, including required Evaluation Plans (for the Evidence Act or other legislation), Memoranda of Understanding (MOUs), and contracts (e.g., with affiliated academic institutions), where appropriate. The Operations Core is responsible for internal coordination of the evaluation teams for each evaluation plan. The Operations Core will regularly engage with PEPReC and VA Central Office leadership to draft a brief description of annual activities for a given evaluation topic, specify quarterly milestones, and produce reports of progress towards each milestone as well as convene working groups involving relevant operations partners for specific evaluation plans, where appropriate.

2. The **Methods Core** is responsible for preparing and executing evaluation plans, including study design and quantitative and qualitative analyses. This Core will be responsible for designing and supporting the methods used across the evaluations of program or policy effectiveness as well as implementation effectiveness, fidelity, reach, etc., and clinical and policy impacts. This Core should support program or policy evaluations that involve a comparison group, e.g., use of randomization or cluster randomization, or that employ rigorous econometric methods and/or clinical pragmatics trial designs. The Core is also responsible for large-scale analytic database design and construction to answer questions on multi-level impacts of programs or policies, and outcome measures, including quality, cost, equity, provider and consumer experience, and value.

3. The **Knowledge Translation Core** is responsible for managing, disseminating, and communicating evaluation results to QUERI Central Office, PEPReC, and VA operations partners and leadership. This Core should develop a knowledge translation playbook (dissemination plan) for each proposed evaluation plan that is based on the QUERI Implementation Roadmap and ACTION Impact Framework that includes communication strategies across different interested groups, business case analyses, and implementation plans in addition to traditional scientific publications and conferences. The Core should respond to inquiries from operations partners, policymakers, and the research community and provide mentoring support for early career investigators with articulated career development goals in evidence-based policy, including successful applicants from the QUERI Advancing Diversity in Implementation Leadership (ADIL) initiative.

Accountability and Review. Due to the Center's unique reporting and scheduling requirements related to the Evidence Act, QUERI Central Office will review Center work regularly with annual funding contingent on satisfactory review and completion of planned milestones. Centers will be expected to provide updates on evaluation plan progress and will be reviewed on a quarterly basis, with an emphasis on milestone achievement and deliverable production.

The long-term goal is that the Centers will add new evaluations each year. Addition of new evaluations and approval of funds to support those evaluations will be at the discretion of QUERI Central Office and will be dependent on VA/VHA evaluation needs, requests from operations partners, and the Center's past track record of milestone completion.

B. Implementation and Evaluation Coordinating Center

The Implementation and Evaluation Coordinating Center coordinates and supports time-sensitive evaluation needs across diverse interested groups, including VA investigators, Program Offices, VISNs, facilities, and providers. Between QUERI leadership, PEPReC, and the Coordinating Center, time sensitive evaluations will be assigned to the field (see Figure 3). In coordination with PEPReC, this Center fulfills a core requirement of the Evidence Act, namely the implementation of training in evaluation and implementation methods. In addition to providing training and consultation in evaluation, QI, and implementation practice methods, this Center coordinates key QUERI rapid response mechanisms, implementation support, and mentoring/training opportunities.

The Coordinating Center must include the following Cores:

1. The **Operations Core** oversees the administration of the Coordinating Center and provides rapid consultations and guidance to diverse groups. This Core identifies emerging time-sensitive evaluation requests from operations partners for assignment to QUERI Program Rapid Response Teams or to other ORD capacity-building initiatives (e.g., HSR&D Researchers and Evaluators in Residents, Service-directed Research), in coordination with other HSR&D/QUERI resource centers. The Operations Core coordinates processes to support QI, evaluation, and implementation practice methods among VA clinical operations, based on the QUERI Implementation Roadmap and ACTION Impact Framework.

2. The **Partnership Core** will be responsible for developing partnerships across VA and supporting operations needs related to VA's fulfillment of the Evidence Act and other federal mandates and time-sensitive priorities. The Core should have capacity to provide brief, implementation planning, evaluation, and training consultation (e.g., review implementation plans for high priorities, matchmake operations (e.g., Program Office, VISN) evaluation needs to QUERI resources and potential investigator teams) to VA and VHA leadership. In addition, the Core will lead national trainings in evaluation methods for VA operations leaders, Program Offices, and other entities to support their responsiveness to the Evidence Act and related evidence-based policy mandates, in coordination with other HSR&D/QUERI resource centers.

3. The **Learning Core** will coordinate and assess capacity across QUERI learning hubs and Program Mentoring Cores, as well as other VA learning opportunities (e.g., Evaluation Boot Camp) to ensure optimal capacity. This core will organize learning collaboratives and field-based meetings, develop materials, and provide guidance on implementation, evaluation, and QI training opportunities and products for different audiences in VA, including managers, leaders, clinicians, and researchers.

Figure 3. Evaluation Center Coordination

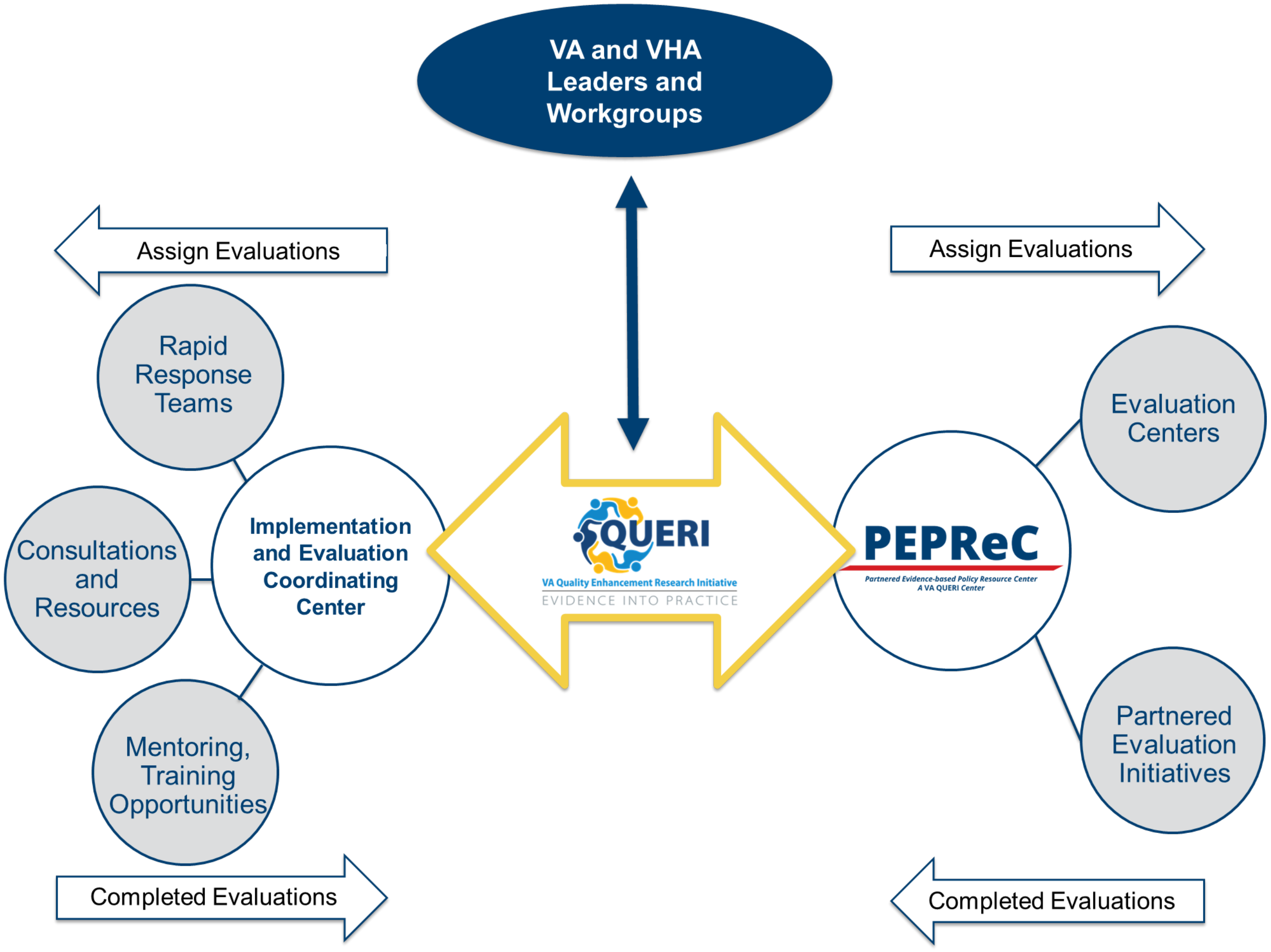

Section II. Award Information

NOTE: Proposals electronically submitted to QUERI through Grants.gov will be peer-reviewed by an ad hoc expert panel, to provide the Director of QUERI with evaluations of the quality of the research proposed and make recommendations on scientific merit, budgets, and funding durations. The final funding decisions by QUERI will include consideration of the overall value of the study to QUERI's investment in improving Veteran care.

1. Mechanism of Support

This FOA/RFA will use the non-HHS Research Collaboration Award (I50) mechanism for VA research.

Before funds are released, all applicable regulatory and compliance approvals must be obtained.

2. Application Types Allowed

Refer to the VA -SF424 AG for guidance on how to fill out the VA-SF424 Cover Form for each application type.

Note: Resubmitted applications should be marked as "Resubmission" in Box 8 of the SF424 (R&R) Form. Renewal applications should be marked as "Renewal" for only the initial submission; the next successive submission must be marked as "Resubmission".

New: Proposals that have not been previously reviewed or funded under this FOA/RFA will be accepted as "new" in response to this FOA/RFA.

Resubmissions: Submission of up to two revised applications (resubmissions) is allowed if the initial submission is not selected for funding.

Renewals: Not applicable.

3. Multiple Awards and Submissions

Applicants may submit more than one application to HSR&D per review cycle in response to the same FOA/RFA or to multiple FOA/RFAs. Applicants may receive funding for more than one HSR&D project. Please be sure to submit your application to the correct VA-ORD Service. Application packages are not interchangeable between R&D Services, nor between FOA/RFAs within a specific service.

4. Funds Available and Waivers

Awards are expected to be funded through VA special purpose funds and MUST be spent within the specified fiscal year. Up to four Evaluation Centers and one Coordinating Center are expected to be funded. The budget provided by QUERI may not exceed the amount described in Table 1. All funding is contingent on available funds and adjustments to budgets may be imposed after an award is initiated.

Table 1. QUERI Funding, Duration, and Co-Funding Requirement

Type of Award	Total QUERI Funding Available	Duration	Co-Funding Requirement
A. Evaluation Center	Up to $820,000/year	5 years	None
B. Implementation and Evaluation Coordinating Center	Up to $820,000/year	5 years	None

Rare exceptions may be granted to the budget cap prior to proposal submission for compelling circumstances. Total project budgets that exceed the budget cap indicated in Table 4 **will not be accepted for review unless a waiver is obtained.** Exceptions may be requested in the form of a waiver submitted to vhacoscirev@va.gov. Standard due dates apply; see Table 5.

Budget Waivers

If a budget cap waiver is granted, a copy of the waiver approval letter from HSR&D must be included in the Letters of Support section of the *VA-ORD SF424* (under Other Project Information, Other Attachments). **A waiver does not guarantee that a project will be funded at the level requested.**

NOTE: In cases where budget waiver requests have been approved for prior submissions to an application, the approval documentation should be included in the Letters of Support section of the new application (resubmission). Prior budget waiver request approvals may be used only if **all** the criteria below are met:

1. The proposal is being submitted in response to the same FOA/RFA.
2. There are no gaps between review cycles (For example, if the initial application was submitted in the winter cycle, the resubmission must be in the summer, not the following winter).
3. The scope of the project remains the same.
4. The total budget remains the same.

If any of the criteria above is not met, a new budget waiver request must be submitted for approval and the approval documentation from QUERI must be included in the **Letters of Support** section of the current application (or resubmission).

If additional budget is being requested, the applicant must include a detailed justification letter with the following components:

1. A cover sheet listing the following information in the order specified:
    a. VAMC name and address
    b. PD/PI's name and degree(s)
    c. PD/PI's title and VA appointment (in 8ths)
    d. Title of PD/PI's proposal (for ongoing programs)
    e. Name, title, and signature of the Associate Chief of Staff for Research & Development
    f. Name, title, and signature of the Medical Center Director

2. A narrative (1-page limit) describing the following:
    a. Explain why the project requires special funding consideration based on the topic, the nature of the study, unusual resource requirements or other factors.
    b. Describe how the proposed project could be completed or modified if the request to exceed the budget limit is denied.
    c. For resubmissions, describe whether an increase in funding is being requested in response to reviewer comments, and if so, please cite the specific comments.

3. Provide a budget for the proposed project.
    a. Include total costs and specify major elements of the personnel, equipment, consultants, supplies and all other expense categories.
    b. Justify each category.
    c. For the equipment category, the justification must include a discussion of why the equipment is needed and why existing equipment cannot be used. Describe the equipment used or to be used.

Off-Site Waivers

Guidelines for submitting an application for an off-site waiver are described in the Program Guide 1200.16: Offsite Research. Standard due dates apply; see Table 5**.** A copy of the approval letter for the off-site waiver must be included in the Letters of Support section of the *VA-ORD SF424* (under Other Project Information, Other Attachments). Although the use of VA-leased space does not require an off-site waiver, VA-ORD must approve a plan for local VA oversight of the research activities performed in the leased space.

Eligibility Waivers

To meet the special needs of VA, exceptions to the eligibility requirements are considered on a case-by-case basis. The facility Director, on behalf of the prospective investigator, must submit such requests in writing to the Director of QUERI, with the endorsement of the facility Associate Chief of Staff (ACOS) for R&D and the facility Chief of Staff. Requests for a waiver of the 5/8ths eligibility criterion must be made 30 days in advance of the submission deadline (Table 5). The approval letter of an eligibility waiver from the Director of QUERI must be included with the Medical Center Director's Letter of Support as an attachment.

Waivers for non-Veteran:

HSR&D **WILL NOT** require non-Veteran enrollment waiver requests prior to funding decisions being made. If your project is selected for funding and you will be enrolling non-Veterans, you will be asked to submit a non-Veteran enrollment waiver during the prefunding process.

5. Location of Research Space

It is expected that the PD/PI and VA co-investigators will perform all funded research in VA space or VA leased space. If any portion of the proposed work will be carried out in laboratory space assigned to (i.e., controlled by) a PD/PI or VA co-investigator/collaborator at any other location(s), a waiver to perform the research offsite

must be obtained for that investigator **prior to submitting the proposal** (refer to Program Guide 1200.16). The use of an off-site core facility or an offsite non-VA collaborator's laboratory does **not** require an off-site waiver, except when the VA investigator is the director of the core facility.

Guidelines for submitting an application for an off-site waiver are described in the Program Guide 1200.16. Standard due dates apply; please see Table 5. A copy of the approval letter for the off-site waiver must be included in the "Letters of Support" section of the "Other Attachments" (Item 12) in the "Other Project Information" form of the SF424 (R&R) Application.

Although the use of VA leased space does not require an off-site waiver, VA-ORD must approve a plan for local VA oversight of the research activities performed in the leased space (refer to Program Guide 1200.16).

6. Duplicate Submissions

An application that is submitted to this FOA/RFA may not be submitted concurrently to any other Funding organization or other component of VA-ORD (i.e., Biomedical Laboratory Research and Development (BLR&D) Service, Clinical Science Research and Development (CSR&D) Service, or Rehabilitation Research and Development (RR&D) Service).

Section III. Eligibility Information

1. Eligible Institutions

All VA medical centers with an active research program are eligible. Each VA medical center must be registered as an applicant organization in Grants.gov and eRA Commons before any proposals can be submitted.

2. Eligible Individuals

The Merit Review Award Program is an intramural program to fund research conducted by VA-salaried investigators at VA medical centers or VA-approved sites. A PD/PI shall hold an MD, PhD, or equivalent doctoral degree in a medical, biological, or behavioral science field.

To be eligible to submit a Merit Review proposal to HSR&D, the PD/PI of the project must have a VA paid appointment of at least 25 hours per week (5/8ths) **at the time the Merit Review Award is funded. Contract clinicians cannot be VA employees (i.e., have a direct, VA-paid appointment) and therefore may not seek funding from ORD, even if the terms of the contract permit or include research activities.**

The VA employment status, including a 5/8ths appointment of each PD/PI must be indicated in the letter of support of the Medical Center Director in the application. If a clinician PD/PI does not have a current, 5/8ths VA paid appointment then the letter of support from the Medical Center Director must include a commitment to offer the PD/PI a 5/8ths (or greater) appointment at the VAMC if the application is approved for funding.

In addition, you (as the PD/PI) must be current with all requirements related to intellectual property (VA invention documents and certifications), submission of annual and midyear progress reports, clinical trials registration, and clinical trials results reporting for existing and previous awards.

To meet the special needs of VA, exceptions to the requirements for eligibility are considered on a case-by-case basis. The facility Director, on behalf of the prospective investigator, must submit such requests in writing to the Director, QUERI, with the endorsement of the facility ACOS for R&D and the facility Chief of Staff. Requests for a waiver of the 5/8ths eligibly criterion must be made 30 days in advance of the submission deadline (Table 5). The approval letter of an eligibility waiver from the Director, QUERI must be included with the Medical Center Director's Letter of Support as an attachment.

Non-VA investigators who have an MD/PhD equivalent are eligible to serve in the role of Co-investigator, **but they cannot be listed as such on the budget forms**. The Co-investigator role may be described in

the proposal narrative and in the written budget justification. **On the budget forms they should be reflected as a consultant or as having an Intergovernmental Personnel Act (IPA) assignment, if appropriate**. If they are providing research services to the VA through a contract, the cost of the contract should be included on the budget forms under all other expenses. Collaborators from outside of the U.S. may only serve as unpaid consultants.

A Site PI must meet the same qualifications as a Study PI; this includes a minimum of a 5/8th VA appointment or waiver of the 5/8th appointment eligibility requirement, an MD/PhD or equivalent; and be registered in ePromise at their current site.

See Program Guide 1200.15: Eligibility for VA Research Support for additional guidance.

VA-ORD will not accept or review an application from an applicant who has an overdue report (e.g., annual progress reports or Research Performance Progress Reports/RPPRs), final reports, clinical trials registration, and results reporting (i.e., ART/clinicaltrials.gov) for existing and previous awards.

Multiple PDs/PIs. One corresponding PD/PI and up to 2 MPIs are allowed. It is the responsibility of the Contact PD/PI to communicate all information to project staff. The decision of whether to submit an application with a single PD/PI or multiple PD/PIs is the responsibility of the "contact" PD/PI identified in Box 14 of the SF424 (R&R) Cover Form and applicant VA medical center and should be determined by the goals of the project. The "contact PD/PI" must be affiliated in the eRA Commons with the VA medical center, be the primary lead on the proposed work, and be the contact for all communications about the proposed work. Only individuals assigned the PD/PI role in the Budget Form and the Key Personnel Form are considered as PD/PIs. All PD/PIs must meet the eligibility requirements described above. The justification for inclusion of more than one PD/PI must be included in a Multiple PD/PI Leadership Plan and may be considered by reviewers as part of their evaluation of the application. Co-PD/PI role is no longer recognized by eRA or VA-ORD. Identification of multiple PDs/PIs may not be used to exceed budget caps. See also the HSR&D Multiple Principal Investigator (MPI) Eligibility Policy at http://www.hsrd.research.va.gov/funding/multi-pi-policy.pdf.

Section IV. Application and Submission Information

Several registration processes must be completed by the local R&D Service before submission of an electronic application (see Section 1.5 of the VA-SF424 AG). Applications must be submitted to Grants.gov by the local research signing official (SO). Applicants are highly encouraged to start the submission process well in advance of the submission deadline to ensure it passes the validations performed at Grants.gov and the eRA.

1. Intent to Submit

QUERI requires Intent to Submit (ITS) notification through the ART website.

The ITS is required for this funding opportunity. The ITS is a process separate from the requirements for Grants.gov submission. The ITS is a key step in the proposal submission process and assists investigators by ensuring that their research is appropriate to the goals of QUERI and VA. ITS is an electronic submission which can be accessed at http://art.puget-sound.med.va.gov/. **The ITS deadline may be found in Table 5.** The ITS process is separate from the requirements for Grants.gov submissions.

Note: Letters of support are **not** required for the ITS.

Completion of the Involved Personnel and Collaborators Spreadsheet information in ART. **This is a fatal error if not completed.**

A list of **ALL named** personnel and collaborators must be updated in your ITS between November 15, 2021 and December 14, 2021 (2-business days after the Grants.gov submission deadline).

ALL personnel and collaborators who are named in the application, including but not limited to: PD/PI(s), co-investigators, personnel with any role in the study, IPAs, consultants, mentors, collaborators, advisory panel members, letter writers, active partners (Program offices) must be included. If someone is only named in the bibliography or biosketch, they do not need to be included.

If the information is not added to the ITS in ART, this will be considered a fatal error and your application may not be reviewed.

NOTE: A new ITS must be submitted each cycle. **Applications submitted to Grants.gov without a completed ITS will not be accepted or reviewed.**

NOTE: The ITS title and the full proposal title must match. Title change requests must be submitted to vhacoscirev@va.gov by the deadline found in Table 5.

2. Request Application Information

Use either **Grants.gov Workspace Process** or **eRA Commons Application Submission System & Interface for Submission Tracking (ASSIST)** to prepare and submit an application in response to this FOA/RFA.

Training resources for the **Grants.gov Workspace Process** are available at https://www.grants.gov/web/grants/applicants/workspace-overview/workspace-process.html. Additionally, there are several videos available at https://www.youtube.com/user/GrantsGovUS.

eRA Commons ASSIST training resources (a recorded presentation, user guides and some other helpful resources) are available at https://era.nih.gov/era_training/assist.cfm (note that VA applications are Single-Project). A NIH/VA-ORD Webinar recording on the use of ASSIST can be viewed at: http://vaww.research.va.gov/funding/electronic-submission.cfm at the bottom under Helpful Links for eRA Commons or http://vaww.research.va.gov/funding/default.cfm under Application & Submission Process.

3. Content and Form of Application Submission

Prepare all applications responding to this FOA/RFA using the SF424 (R&R) application forms in accordance with the VA Application Guide SF424 (R&R) found at http://vaww.research.va.gov/funding/electronic-submission.cfm. A summary of the main components required for this application is shown below in Table 2. Table 3 contains instructions for SF424 Research and Related Forms specific to this FOA/RFA. **The instructions in Table 3 may differ from – in which case they supersede – the general instructions contained in the VA-SF424 Application Guide.** Unless otherwise noted, all instructions contained in the VA-SF424 Application Guide must be followed. Failure to follow instructions may cause delays in submission or withdrawal of proposals from review.

Use of hyperlinks: All applications must be self-contained (i.e., without the use of URLs/hyperlinks), within specified page limits. The use of URLs/hyperlinks is prohibited except in the Biographical Sketch and Bibliography & References Cited attachments. Any submission with URLs placed anywhere else except in the Biographical Sketch, and Bibliography & References Cited attachments will be withdrawn from review. Waivers are prohibited for URLs/hyperlinks. **NOTE: URLs/hyperlinks within official documents that cannot be altered, such as letterhead (i.e., Letters of Support attachment) or published articles/manuscripts (i.e., Appendix attachments) will be accepted.**

The inclusion of links to videos within an application is not acceptable and will cause the application to be withdrawn from review.

QUERI will only accept videos for demonstration of devices, products under development or interventions aimed at providers or patients that cannot be sufficiently depicted in text or screenshots. **The video cannot be included in the application in any attachment**; this will cause the application to be withdrawn from review. PIs must contact the SPM at least 3 weeks prior to the application deadline for approval to submit supplemental video material. If the SPM approves, the SPM approval email must be included as a PDF

attachment to the application (Item 12, Other Attachments, 8b. Letters of Support). The video must be embedded in a .pdf file with a maximum file size of 25 MB, not to exceed 2 minutes, and submitted directly to the SPM prior to the application deadline. **A missing SPM approval email attachment will cause the application to be subject to withdrawal from review.**

Information Regarding Attachments for Item 12: The file names for Attachments 1 – 9 are **mandatory** and may not be changed. Altered file names will cause an **error** to be generated. Only the descriptor in the file names for Appendices 10, 11, 12… may be changed. Altering any other part of the file name may result in parts of your application being excluded from the final electronic image that the reviewers receive or for the attachments to appear in the wrong order.

A set of templates, with mandatory file names for each attachment, is available on the VA-ORD intranet site (http://vaww.research.va.gov/funding/electronic-submission.cfm). Information for each attachment in Item 12 must be saved in a single PDF file and attached.

Table 2. Summary of Required Forms and Attachments for this FOA/RFA

Forms, Attachments, and Templates with Size Limits as Applicable	Required When?	VA-SF424 Instructions
SF424 (R&R) Form	Always	Section 3.2
Project/Performance Site Locations Form	Always	Section 3.3
SF424 (R&R) Other Project Information Form:		Section 3.4
Project Summary/Abstract (40 lines of text)	Always	
Project Narrative (10 lines of text)	Always	
Bibliography & References Cited (4-page limit)	Always	
Facilities & Other Resources	Always	
Equipment	Always	
Other Attachments (Item 12):		
1. Introduction to Revised Application (3-page limit)*	Resubmission	
2. Specific Aims (1 page limit)* List the specific aims in a numbered list, with a description of the component of the study relevant to each Aim.	Always	
2a. Research Plan*	Always	
2b. VA Career Plan	Never Submit	
2c. Mentoring Plan	Never Submit	
3. Progress Report	Never Submit	
4. Human Subjects*	If Applicable	
5. Vertebrate Animals	Never Submit	
6. Multiple PD/PI Leadership Plan*	If Applicable	
7. Consortium/Contractual Arrangements*	If Applicable	
8. Director's Letter*	Always	
8a. R&D Committee Letter	Never Submit	
8b. Letters of Support*	Always	
9. Data Management and Access Plan*	Always	
10. Financial Disclosure*	Always	
Appendices:*		
11. List of Abbreviations*	Always	
12. Executive Summary*	Always	
SF424 (R&R) Senior/Key Person Profile(s)*	Always	Section 3.5
SF424 (R&R) Budget*	Always	Section 3.7

* These sections have special instructions for this FOA/RFA that are in addition to or supersede instructions in the VA SF424. See Tables 8 and 9 below.

The instructions below consist of QUERI-specific instructions for completing the SF424 Form and Other Attachments in Section 3.4 of the SF424. All SF424 instructions must be followed, but you will find HSR&D-specific clarifications and instructions below.

Table 3. QUERI-Specific Instructions for SF424 (R&R) Forms and Attachments

**Other Attachments**	**1. Introduction to Revised Application (for Resubmission only)**
**SEE SF424, SECTION 3.4, ITEM 12, FOR ADDITIONAL INSTRUCTIONS.**<br><br>**PAGE LIMIT: 3**<br><br>The substantial scientific changes must be marked in the text of the application by bracketing, indenting, or change of typography. A vertical bar drawn in the margin may be used as long as changes in text are also indicated by bracketing, indenting or change of typography. Do not underline or shade the changes. Deleted sections should be described but not marked as deletions. If the changes are so extensive that essentially all text would be marked, explain this in the Introduction.	
**Other Attachments**	**2. Specific Aims**
**SEE SF424, SECTION 3.4, ITEM 12, FOR ADDITIONAL INSTRUCTIONS.**<br><br>**PAGE LIMIT: 1**<br><br>Provide a concise summary of the Center's overall goal, specific objectives, methods used in the Cores, experience with evaluations in partnership with VA operations leaders, and potential impact of the proposed Center.	
**Other Attachments**	**2a. Research Plan**
**SEE SF424, SECTION 3.4, ITEM 12, FOR ADDITIONAL INSTRUCTIONS.**<br><br>Acceptance by QUERI to review a revised application automatically supersedes previous submissions and the revised application becomes the document of record.<br><br>**A. For Evaluation Center proposals, the Research Plan must include the following sections:**<br><br>**Page Limit: 14**<br><br>**Background:** Describe the overall goals of the Center and identify evaluation priority areas listed in this RFA that the Center will focus on, and in particular, the significance of the issues addressed and how investigators and staff have particular expertise and experience in conducting evaluations in those areas. Describe each Core, highlighting the experience of the investigator and staff team in conducting rigorous evaluations, management of multiple, time-sensitive evaluation projects, and knowledge translation of evaluation findings (e.g., based on the QUERI ACTION Impact Framework). Applicants are encouraged to include a conceptual framework to illustrate how each Core relates to the overall goals of the Center and for the VA.<br><br>**Methods:** Give a general description of the experience and expertise of the Center staff, including but not limited to quantitative and qualitative analyses, data analytics, policy, organizational, operations, economic analyses, implementation science (including evaluation of implementation strategies), QI, and/or knowledge translation methods.<br><br>The methods section should include a summary of two proposed evaluation plans to be conducted in the first year based on the priority topics listed in the RFA and the goals of the proposed Center. It is highly	

encouraged that the proposed evaluations address topics that are national in scope, involve questions that transcend a single program office, and include questions that are not already covered by existing QUERI evaluations. See the National Network of Centers tab on the QUERI website and the QUERI brochure for a list of currently funded QUERI evaluations.

The evaluation plan should specify the objectives, brief background of the problem the evaluation is to address, study design, study population and sampling strategy, data sources, measures, and how data will be collected to support evaluation efforts. These evaluation plans may follow the format provided starting on page 7 in the FY2022 VA Evaluation Plans.

Justify the designs and data sources based on feasibility, timeliness and validity of data, and costs, and provide a timeline of evaluation activities by quarter for each evaluation plan.

Describe principal outcomes, including quality, cost, health equity, and patient and provider experience.

Measures should be described in detail and identify specific covariates of interest and how they will be initially analyzed.

Explain any potential obstacles and solutions [e.g., timing of data collection, timing and unit of randomization, survey feasibility, subject burden, selection effects] that may arise.

Note: If conducting a randomized program evaluation including cluster-randomized trials, investigators are strongly encouraged to register their study protocols with ClinicalTrials.gov or ISRCTN and publish their trial protocol.

Partnerships (including Contracts), Center Team, and Relevant Experience: Describe key participants (co-investigators and organizations) and experience with partnerships with VA national program offices, VISNs, or other partners and in working with VA national evaluation efforts. Describe the role and tasks of each member of the investigative team and how their work will be coordinated. Describe local resources available at your facility that will lend to your team's ability to be successful. Describe experience and capacity to contract with experts external to VA for evaluation and implementation methods, such as through universities or other institutions.

Management Plan and Timeline: Describe, in detail, regular deliverables and their timelines. The exact details will be negotiated based on the evaluation needs of VA and QUERI leadership, and include timing of regular meetings, securing of data use agreements where applicable, and creation of a memo of understanding outlining specific deliverables within the first three months of project award.

B. For Implementation and Evaluation Coordinating Center proposals, the Research Plan must include the following sections:

Page Limit: 14

Background: Describe the overall goals of the Center and identify consultation and training that the Center will focus on, and in particular, the significance of the gaps in knowledge and need for knowledge translation in VA and how investigators and staff have particular expertise and experience in conducting evaluation, training, coordination, and consultation. Describe each Core, highlighting the experience of the investigator and staff team in conducting rigorous evaluations, management of multiple, time-sensitive evaluation projects, training in implementation and evaluation science, and consultation across multiple interested parties (e.g., Veterans, providers, operations partners, VA and VHA national and VISN leadership, Veterans Service Organizations, scientific community) based on the QUERI Implementation Roadmap and ACTION Impact Framework. Applicants are encouraged to include a conceptual framework to illustrate how each Core relates to the overall goals of the Center and for the VA.

Methods: Give a general description of the experience and expertise of the Center staff, including but not limited to evaluation (quantitative and qualitative analyses, data analytics, policy, organizational, operations, and/or and economic analyses), training and use of implementation and QI science (including evaluation of implementation strategies), and consultation and knowledge translation methods.

The methods section should describe the review process for rapid-response evaluation requests (e.g., QUERI Program Rapid Response Teams).

This section should describe the activities to be undertaken by each Core.

For the Learning Core, describe the objectives and coordination approach across training opportunities in QUERI and elsewhere and an evaluation of training goals and impacts. Describe in-house training opportunities within the Coordinating Center, including materials, competencies, mentorship, consultation, and plans to evaluate impact of the training and consultation process.

Partnerships (including Contracts), Center Team, and Relevant Experience: Describe key participants (co-investigators and organizations) and experience with partnerships with VA national program offices, VISNs, or other partners and in working with VA national training, consultation, rapid response review, and evaluation efforts. Describe the role and tasks of each member of the investigative team and how their work will be coordinated. Describe local resources available at your facility that will lend to your team's ability to be successful. Describe experience and capacity to contract with experts external to VA for evaluation and implementation methods, such as through universities or other institutions.

Management Plan and Timeline: Describe, in detail, regular deliverables and their timelines. The exact details will be negotiated based on the evaluation, training, rapid response review, and consultation needs of VA and QUERI, and include timing of regular meetings, securing of data use agreements where applicable, and creation of a memo of understanding outlining specific deliverables within the first three months of project award.

**Other Attachments**	**4. Human Subjects**

SEE SF424, SECTION 3.4, ITEM 12, FOR ADDITIONAL INSTRUCTIONS.

NOTE: This RFA will support projects that are considered as non-research quality improvement, per Program Guide 1200.21, because they involve extensive input from VA operational partners, are more evaluative in nature, and are not designed to contribute to generalizable knowledge.

If the proposed work is considered non-research, check the box marked "No" for Question 1 on the Other Project Information.

Non-research letters are **not** required and will **not** be accepted. **All proposals MUST include, as part of this section, a description of how the work is considered non-research, based on Program Guide 1200.21.**

**Other Attachments**	**6. Multiple PD/PI Leadership Plan**

SEE SF424, SECTION 3.4, ITEM 12, FOR ADDITIONAL INSTRUCTIONS.

A leadership plan is required if more than one individual is assigned the role of PD/PI in the Senior/Key Person Profile(s).

Multiple PDs/PIs on a project share the authority and responsibility for leading and directing the project, intellectually and logistically. Each PD/PI is responsible and accountable to VA for the proper scientific, fiscal, and ethical conduct of the project, including the submission of all required reports.

The rationale for choosing a multiple PD/PI approach must be clearly described in the plan. The governance and organizational structure of the leadership team and the research project must be described, including the communication plan, process for making decisions on scientific direction, and procedures for resolving conflicts. The roles, knowledge, skills, and experience, and administrative, technical, and scientific responsibilities for the project or program, must be clearly described for each PD/PI, in addition to the distribution of resources to specific components of the project or to individual PDs/PIs. These components will be factored into the assessment of the overall scientific merit of the application. See http://www.hsrd.research.va.gov/funding/multi-pi-policy.pdf.

Other Attachments	7. Consortium/Contractual Agreements

SEE SF424, SECTION 3.4, ITEM 12, FOR ADDITIONAL INSTRUCTIONS.

If applicable: This attachment should only be used to describe existing consortium or contractual agreements that are relevant to the proposed research; new agreements to perform a portion of the proposed research will not be considered binding to VA.

Do not include IPAs here.

This attachment should not be used to describe or to justify the required sub-award budgets for multi-site projects.

Explain the programmatic, fiscal, and administrative arrangements that exist between the applicant VA Medical Center and any consortium or contractual organization(s).

New consortium on contractual agreements will not be considered binding to VA contractually.

Other Attachments	Director's Letter

SEE SF424, SECTION 3.4, ITEM 12, FOR ADDITIONAL INSTRUCTIONS.

All proposals must include an attachment containing a signed (e-signature accepted) and dated (within the last year) copy of a letter of support from the Director of the Medical Center documenting that sufficient resources (i.e., space, equipment, time, appointment, etc.) are available to the investigator.

The Letter of Support from the Medical Center Director must indicate the VA employment status, including 5/8^{ths} appointment of each PD/PI. If a clinician PD/PI does not have a current, 5/8ths VA paid appointment then the letter of support from the Medical Center Director must include a commitment to offer the PD/PI a 5/8ths (or greater) appointment at the VAMC if the application is approved for funding.

Note: New requirements outlined in the SF424 require that the Directors letter include language supporting protected time for clinician researchers (see *VA-ORD SF424*).

Other Attachments	8b. Letters of Support

SEE SF424, SECTION 3.4, ITEM 12, FOR ADDITIONAL INSTRUCTIONS.

Please DO NOT send separate original hard copies or email PDF copies of Letters of Support to the Director of QUERI. For these letters to be considered as a part of the proposal, they must be included as part of the Letters of Support attachment (Item 12, Other Attachments, 8b. Letters of Support). Letters should be submitted on the official letterhead of the individual's supporting institution.

All memoranda should be addressed to the Director, QUERI, and must include the Corresponding PI's name, project title, VA facility, signature, and date.

For Resubmission applications: A Letter of Support needs to be resubmitted if the original letter is not dated within the last year, i.e., it can't be more than one year since the letter of support was first submitted.

Letters of support from Program Offices and VISNs are <u>not</u> required and will <u>not</u> be accepted. Do not include letters of support or non-research letters from Program Offices or VISNs in your application.

QUERI does not require letters of support from MPIs or co-investigators. Letters from consultants and Medical Center Directors are required. The consultant letters must indicate concurrence of the consultant with their specific role or contribution as described in the application, their willingness to fulfill the duties described in the application, and their rate/charge for consulting services, if applicable.

Waivers: This section must include approval letters for all waivers (budget cap, off-site, eligibility, inclusion of videos).

**Other Attachments**	**9. Data Management and Access Plan**

A Data Management and Access Plan (DMAP) **is not required** for this QUERI award. However, you **must** upload an attachment with the file name 09_VA_DMAP.pdf and the content "Not Applicable," or you will receive a system error message.

**Other Attachments**	**10. Financial Disclosure**

SEE SF424, SECTION 3.4, ITEM 12, FOR ADDITIONAL INSTRUCTIONS.

See the VA-ORD Application Guide SF424 (R&R), Attachments for Item 12, for guidance on this document.

A sample financial disclosure document can be found at: http://vaww.research.va.gov/funding/electronic-submission.cfm.

You **must** upload the attachment with the file name 10_VA_Financial_Disclosure.pdf, or you will receive a system error message.

**Other Attachments**	**11., 12., 13... Appendices**

SEE SF424, SECTION 3.4, ITEM 12, FOR ADDITIONAL INSTRUCTIONS.

<u>List of Abbreviations:</u>

An alphabetized list of abbreviations used in the application should be attached as the first Appendix document.

The filename should be the following: **11_VA_Appendix_1_Abbreviations.pdf**

<u>Executive Summary:</u>

An executive summary is REQUIRED for all QUERI applications. Please include a 1-2 page executive summary for your proposed work. The executive summary should be brief – bullet points are encouraged.

Key Components of executive summaries:

- Title of project
- Problem in VA your proposal is addressing
- Aims
- Brief description of project(s), implementation/evaluation strategies, and linkage to RFA priorities and VHA Performance Plan metrics

○ Anticipated Impacts: Provide a few sentences or bullet points describing VA national practice or policy changes resulting from the evaluations.<br>○ Visual (e.g., infographic, chart, graphical approach/methods)<br>○ Center expertise based on RFA priorities<br>○ Corresponding PI and MPIs (if applicable)<br>○ Key operations partners<br><br>The filename should be the following: **12_VA_Appendix_2_ ExecutiveSummary.pdf**<br><br>**<u>Other:</u>**<br><br>Do not include Informed Consent forms as an appendix, even if already approved by the IRB.<br><br>• **Note: Published manuscripts and/or abstracts that have a free, publicly available online journal should not be included in the appendix material.** The full reference should be included in the Bibliography & References Cited section, and/or the Biographical Sketch section, as appropriate.<br>• **No videos of any type will be accepted**, whether linked to in a URL or embedded in the PDF.<br>• All materials must be submitted electronically in PDF format.	
**SF424 (R&R) Senior/Key Person Profile Form**	**Senior/Key Person Profile(s)**
See *VA-ORD SF424, Section 3.5,* for additional instructions.<br><br>A Senior/Key Person Profile form is required for all involved personnel and collaborators, to include the following:<br>• **Senior/Key Personnel:** All individuals who contribute in a substantive, measurable way to the scientific development or execution of the project, whether or not salaries are requested; this includes PD/PI(s) and Co-I's.<br><br>**Important notes:**<br>• If Centralized Transcription Service Program (CTSP) services are proposed, add Dr. Susan Zickmund as an "Other Professional," by typing "CTSP'" under the Other Project Role category.<br>○ Biosketch or Other Support documents are not required; however, applicants must upload an attachment to both the Biosketch and Other Support fields with the words "Not Required," or they will receive a system error message.<br>If Dr. Susan Zickmund is a collaborator or Co-I on the project (as opposed to CTSP services), please follow the general instructions.	

The instructions below consist of QUERI-specific instructions for completing the Summary Budget Worksheet and R&R Budget Form in Part I, Section 3.7 of the SF424. All SF424 instructions must be followed, but you will find QUERI-specific clarifications and instructions below.

Summary Budget Worksheet
The Summary Budget Worksheet template can be accessed at:
http://vaww.research.va.gov/funding/docs/SummaryBudgetWorksheetTemplate.xlsx.

R&R Budget Form
Please see the VA-SF424 AG, Section 3.7 for instructions on filling out the Summary Budget Worksheet and R&R Budget Form.

The budget table should ONLY REPORT budget items to be covered by QUERI funding.

In the budget justification, clearly delineate on separate pages what items (e.g., personnel, travel, all other) to be covered under QUERI funding and what will be covered by the operations partner. **If applicable, at the top of the justification, briefly describe the amount of operations partner funding (per year and total) and/or other in-kind resources and how this funding and/or resources will be used.**

Table 4. QUERI-Specific Clarifications and Instructions for Budget Items A-K

**Personnel - (SF424, Section 3.7.1, Sections A)**	
• Salary for VA Employees	Salary increases (cost of living adjustments - maximum of 2% per year) are permitted for all current VA salaried personnel (including the contact PD/PI) and may be budgeted in out years (Year 2; Year 3; Year 4). Cost of living adjustments may not exceed the total project budget cap. Salaries are to include actual fringe benefits for all current VA salaried personnel and no more than 30% fringe benefits for all "to be determined" positions.<br><br>QUERI will pay salary only for the actual time the PI or other VA paid study personnel spend on the project. One of the major differences between how BLR&D and HSR&D operates is that BLR&D will pay "up to the entire" VA Salary of a PI on a project, regardless of his/her effort on the project.
• Physicians	Salary support is not authorized for any physician (VA or other salaried) on the QUERI portion of the award.
• Nurses or Licensed Medical Professionals	Salary support is not authorized for any Title 38 or hybrid nurse or licensed medical professional on the QUERI portion of the award.
• Clerical support	Clerical support may not be included as study personnel.
• IPAs	Costs for IPAs must **NOT** be listed under Section A or B of the R&R Budget Component. See "All Other Expenses" section of this table for HSR&D-specific IPA instructions. IPAs must be officially recognized by VA as authorized.
• Consultant	Services may be obtained by contract or through a letter of agreement. Consultant fees will be set in accordance with VHA Handbook 5007. Limit of $500 per consultation and $2,500 per annum. Physicians may not be paid as consultants.
**Equipment - (SF424, Section 3.7.2, Section C)**	
• Computers	Computers (and IT expenditures) should not be listed in the budget section.
**Supplies - (SF424, Section 3.7.3)**	
• Books, journals, or reprints	Not authorized. However, payment for reasonable page/publication costs may be included, up to $3,000, which must be requested as part of the original budget submission.
**All Other Expenses - (SF424, Sections 3.7.2 and 3.7.3)**	
• Salary for Personnel on IPAs	IPAs provide for salary and fringe benefit reimbursements; they do not allow for "overhead" costs. IPAs may not be used for physicians or other clinical medical service providers. IPAs may not be used for any individual assigned the PD/PI role.<br><br>As stated by OPM, "Intergovernmental Personnel Act assignees appointed for more than one year are eligible for within-grade increases. They are entitled to cost-of-living allowances and other pay differentials, and are allowed to accumulate and use leave to the same extent as other Federal employees. However, employees appointed to successive temporary appointments of one year or less may not earn a within-grade increase, even if the time under the successive temporary appointments exceeds one year." https://www.opm.gov/policy-data-oversight/hiring-information/intergovernment-personnel-act/#url=Assignment

	It is essential that core funds go to VA employees since this is an intramural program.
• Travel	Provide detailed travel requests, including justification for site visits, meetings, etc.
• Transcription Services	HSR&D is no longer requiring quotes from CTSP for transcription. If you decide to use CTSP services for transcription, please follow the directions below so that funds can be transferred.<br><br>The project team should contact the CTSP (VHASLCCTSP@va.gov) or Dr. Susan Zickmund, PhD (Susan.Zickmund@va.gov) to request a formal proposal, including cost, for the potential use of CTSP services in the study. The CTSP may be able to provide a more cost effective, secure, and efficient mechanism that is designed to meet the project transcription needs without the need for contracting.<br><br>**If the applicant plans to utilize CTSP services:**<br>• A Letter of Support, Biosketch and Other Support documents **are not required** for Dr. Zickmund (see Table 2).<br>• Include CTSP transcription services costing in the R&R Budget Form.<br>○ If Salt Lake City (SLC) is not already a research site, it should be added as an additional site to the budget. Dr. Susan Zickmund should be listed as the Site Investigator; the Site Investigator is responsible for the funds sent to and the work performed at SLC.<br>○ If SLC is already a research site, Susan Zickmund need not be listed as site investigator if one already exists.<br>▪ Dr. Zickmund's Percent Effort: List "N/A" and list her salary as "contributed."<br>▪ "Other Direct Costs" (in the Summary Budget Worksheet): List "CTSP Transcription Services (SLC)," along with associated costs.<br>• Include a brief description of the CTSP transcription services required in the written Budget Justification.<br>• For Dr. Zickmund's Budget Justification use this text if she is ONLY included for CTSP:<br>Zickmund, Susan PhD (Effort: NA, Salary: Contributed): Dr. Zickmund is a Research Scientist at the Veteran Health Administration Salt Lake City and Director of the VA HSR&D-funded Centralized Transcription Services Program (CTSP). She has supervised thousands of hours of qualitative data collection and analysis including transcription. She supervises employees dedicated to producing verbatim transcriptions for research investigator.

4. Submission Timelines and Processing Information

4.A. Deadline, Review, and Award Dates

Deadlines. Avoid delays and misunderstandings by reading and following the instructions carefully. Table 5 contains deadlines for this solicitation. Depending on the investigator's circumstances, requests for off-site waiver or eligibility determination or approval to exceed budget limits may be needed.

Table 5. Deadline, Review and Award Dates

**SUBMISSION CYCLES**<br>**IF THE DEADLINE FALLS ON A WEEKEND OR FEDERAL HOLIDAY, THE DUE DATE IS THE NEXT BUSINESS DAY.*	**PLATFORM**	**SUMMER 2022**
**Intent to Submit Window***	ART	April 1 – May 13, 2022<br>(8pm EST)
**QUERI RFA Informational Cyberseminar**	Registration Link	April 11 at 1pm ET
**Waiver Submission Deadline**<br>***Click here for more waiver information***	HSR&D SciRev Inbox	May 13, 2022<br>(11:59 pm EST)
**First day to submit application***	Grants.gov	May 15, 2022
**Update Involved Personnel Information on ITS**	ART	May 15 – June 14, 2022<br>(11:59 pm EST)
**Deadline to request application title change***	HSR&D SciRev Inbox	June 1, 2022<br>(11:59 pm EST)
**Down-to-the-Wire Submission Deadline**<br>Submitting by this date ensures applicants will have 2 bus. days to correct errors identified by the system(s).<br><br>Applications submitted *after* this date cannot be corrected/changed.	Grants.gov	June 8, 2022
**Last Possible Submission Date**<br>Applications submitted on this date cannot be corrected/changed.<br><br>**Applications cannot be submitted after this date.**	Grants.gov	June 10, 2022<br>(6:00 pm, local time)
**Verification Deadline***<br>Once the system verifies an application, it is considered final, and no other version will be accepted for review.<br><br>Applications are verified on the 2nd business day after they are submitted (if there are no errors or only warnings).	eRA	June 15, 2022
**REVIEW & AWARD CYCLES**		**SUMMER 2022**
**Scientific Merit Review**		August 2022
**Administrative Review**		September 2022
**Earliest Project Start Date**<br>*NOTE:* VA-ORD R&D Services may not always be able to honor the requested start date of an application; therefore, applicants should make no commitments or obligations until confirmation of the start date by the awarding service.		October 1, 2022

4.B. Application Processing

All new or changed/corrected applications must meet 2 separate deadlines:

1. Submission and acceptance in Grants.gov on or before 6:00 pm (local time) of the **Last Possible Submission Date** (submission deadline) in Table 5

AND

2. Verification by eRA Commons on or before the **Verification Deadline** in Table 5

All applications should be proofread carefully prior to submission.

Applications that miss either deadline will not be accepted for review.
NOTE: Applications accepted by eRA Commons with no errors (with or without warnings) are provided a two-business day examination window to check for errors. The application is automatically verified on the third business day if it is not explicitly rejected (withdrawn) by the signing official (SO) during the 2-day application viewing window. However, if an application is submitted AFTER the Down to the Wire Submission deadline, the 2-day examination window CANNOT be used to identify errors and resubmit a changed/corrected application.

Once an application package has been successfully submitted through Grants.gov, any errors have been addressed, and the assembled application has been created in the eRA Commons, the PD/PI and the Authorized Organization Representative/Signing Official (AOR/SO) have 2 business days to view the application image (ONLY if submitted on or before the Down to the Wire Submission deadline).

Once verified, an application is considered final, and no other version will be accepted for review. It is the responsibility of the PD/PI and AOR/SO to check for errors during the 2-day application viewing window.

During the 2-business day examination period, the electronic image of submitted proposals (e-application in eRA Commons) must be reviewed to ensure that there are no transmission errors. PIs are responsible for printing out and reviewing the electronic image of the e-application during the 2-business day period in order to check the submission for format, transmission, or content errors.

Please remember that some warnings may not be applicable or may only need to be addressed after application submission. Reminder: warnings do not stop further application processing. If an application submission results in warnings (but no errors), it will automatically move forward after two business days if no action is taken.

Applications which fail to follow formatting and content requirements or are incomplete will be administratively withdrawn and not reviewed. No exceptions will be made. It is the responsibility of the PI to check that every page is correct and that all elements of the proposal have been included. After an application has been submitted, the application should be checked for problems with font type, font size, margins, characters per inch and lines per inch. It is advised that PIs print out a page of the Research Plan during the 2-business day examination period and MANUALLY check for these types of errors as eRA does not generate an error message for them. However, such errors WILL cause the proposal to be administratively withdrawn.

QUERI will consider the errors listed below as fatal. Applications submitted with these errors will be administratively withdrawn and will not be reviewed.

- Not including a list of **all** involved personnel and collaborators in ART. This information needs to be submitted in ART by the deadline in Table 5.
- Missing budget page(s) - Applications must include a completed budget page for each fiscal year of study.
- Missing budget or off-site waiver approval letter (if proposed budget exceeds cap specified in RFA or offsite research is proposed).

- Missing documents required for submission.
    - If this is a resubmission, the application must include the “Introduction to Revised Application”.
    - The application must include an Executive Summary in the Appendix.
- Missing or unsigned VA Medical Center Director’s letter of support.
- Exceeding specified page limits.
- Using a version of the biographical sketch other than the one specified in the SF424.
- Any submission with URLs placed anywhere else except the Biographical Sketch, and Bibliography & References Cited will be withdrawn from review.
- Failure to meet specified content or formatting requirements for Text (PDF) attachments in e-application. Research plans should be carefully checked for formatting and PDF conversion errors.

A previously submitted application must be rejected/withdrawn before a changed/corrected application can be submitted.

If an application is accepted by eRA with no errors, do not reject/withdraw an application during the 2-business day examination window unless there is sufficient time to resubmit a changed/corrected application by the submission deadline. The 2-business day examination window CANNOT be used if an application has been submitted on the Last Possible Submission date.

If everything is acceptable, no further action is necessary. The application will automatically move forward for processing after 2 business days and will become verified at 12:01 am on the third business day. Both the AOR/SO and PD/PI will receive e-mail notifications when the application is rejected, or the application automatically moves forward in the process after 2 days.

Once an application becomes verified it is considered final and no changed/corrected application will be accepted for review.

VA-ORD will not penalize the applicant for an eRA Commons or Grants.gov system issue. However, unless there is documentation of a processing error at either Grants.gov or eRA Commons, applications that fail to meet either the submission or verification deadline will not be accepted for review.

Once an application becomes verified, it will be evaluated for completeness by the QUERI program staff. **Applications which fail to meet content and formatting requirements or are incomplete will be administratively withdrawn by QUERI Program Review staff and will not be reviewed.**

No additional or replacement information will be accepted after submission of the proposal, unless requested by the Program Review staff. The only exceptions are official letters of acceptance for publication of manuscripts submitted by the PD/PI. These may be sent by e-mail to the Scientific Merit Review Program Manager (vhacoscirev@va.gov) at any time.

All proposals must include a separate attachment containing a signed copy of the letter of support from the Director of the Medical Center documenting that sufficient resources (i.e., space, equipment, time, appointment, etc.) are available to the investigator. Review of applications submitted to VA-ORD without this attachment will not be accepted for review.

There will be an acknowledgement of receipt of applications from Grants.gov and eRA Commons. The submitting AOR receives the Grants.gov acknowledgments. The AOR and the PD/PI receive eRA Commons acknowledgments.

The eRA system will make every effort to send an email to the PD/PI and AOR/SO summarizing the download and validation results.

NOTE: Since email can be unreliable, it is the responsibility of the applicant and AOR/ Signing Official(s) to periodically check on the application’s status in eRA Commons.

VA-ORD will not accept any application in response to this RFA that is essentially the same as one currently pending initial merit review unless the applicant withdraws the pending application. VA-ORD will not accept any application that is essentially the same as one already reviewed. This does not preclude the submission of an application already reviewed with substantial changes, but such application must include an “Introduction” (3 pages maximum) addressing the previous critiques. Note that such an application is considered a "resubmission" for the SF424 (R&R).

5. Intergovernmental Review
Not Applicable.

6. Funding Restrictions
Not Applicable.

7. Other Submission Requirements
ePromise
The investigator profile (Page 18) in ePromise must be completed (including the Commons ID) for all PDs/PIs.

Section V. Application Review Information

---

1. Criteria
Only the review criteria described below will be considered in the review process.

2. Review and Selection Process
Overview
Applications submitted in response to this FOA/RFA will be reviewed through a two-tier system.

The first level of review will be performed by QUERI’s Scientific Merit Review Board (SMRB), sometimes referred to as a “review panel” or “review committee.” The SMRB is a Federal Advisory Committee Act board charged to evaluate the scientific and technical merit of applications. The SMRB is an advisory committee and does not make funding decisions. Information about SMRB membership may be obtained from the HSR&D web site at http://www.hsrd.research.va.gov/.

The second level of review will be performed by QUERI, based not only on considerations of scientific merit, as judged by the SMRB, but also on the relevance and responsiveness of the proposed project to the mission, programs, and priorities of QUERI. Final funding decisions are made at the discretion, and approval, of the Director of QUERI.

Not Discussed/Unscored Applications
The initial scientific peer review of applications may include a time management process in which only those applications deemed by the reviewers to have the highest scientific merit will be discussed and assigned a priority score at the SMRB meeting. The purpose of not discussing some applications is to increase the time available for providing feedback on studies that have the most potential for funding (either in the current review or a subsequent review). This will also help QUERI to better manage scarce resources.

If an application is not discussed, the PI will not be given a priority score and will be advised that a) the proposal was not discussed by the full panel, and b) any resubmission needs to address the key issues raised in the written critiques. ALL applications are reviewed and receive written critiques; however, not all applications need to be discussed. (An application that is not discussed may be very appropriate for resubmission, depending on the comments in the written critiques.)

Scoring
SMRB members are instructed to evaluate applications using the review criteria described below, and to assign a single, global score for each scored application. The score will reflect the scientific merit of the proposed research and its overall impact on advancing science and the health and health care of Veterans. Other FOAs or RFAs may have different and/or additional review criteria. For information on the scoring guidelines, go to http://www.hsrd.research.va.gov/for_researchers/merit_review/ScoringGuidelines.pdf.

All PIs will receive a written **Summary Statement** which includes a cover page, the Program Description/Abstract section from the submitted application, each assigned reviewer's written comments, and a roster of the review meeting participants.

Criteria for Review and Scoring of the Proposal
The following criteria are considered during scientific merit review:

Significance and Impact: Does the application address an important problem or a critical barrier in evidence-based policymaking in VA and elsewhere? How will the Center's work inform programs and policies affecting Veteran care? Are the concepts, approaches, or methodologies novel and will they inform the way VA approaches evidence-based policymaking? How will the Center's work drive impacts in how evaluation, QI, and implementation are done in VA?

Approach: What is the overall quality of the cores and the adequacy of the services provided? Are the overall strategies, methodologies, and analyses well-reasoned and appropriate to accomplish the goals of the Center? Does the Center use state-of-the-art evaluation, training, and knowledge translation methods that challenge and seek to shift current paradigms in evidence-based policymaking within VA? Do the proposed evaluation plans address high-level policy questions that cut across different program offices or services? Are potential problems, alternative strategies, and benchmarks for success presented? Are data sources and methods appropriate for the proposed work, and do outcomes/measures of success incorporate multiple perspectives (e.g., quality, value, end-user experience, equity, provider/workforce impacts)? Are the milestones realistic, significant and measurable? Is there sufficient evidence to determine that the proposed work can be successfully completed?

Integration of Core Activities: Is there evidence of scientific (e.g., evaluation, implementation, QI) and administrative integration of the proposed Center application? Is there evidence of coordination, interrelationships, and synergy among the individual Cores and evaluation plans? Are there clear advantages or "value added" by conducting the proposed work as a Center rather than conducting individual projects?

Center Leadership and Environment: Are the qualifications, experience, and commitment of the Center, Core leads, and key personnel adequate and appropriate to provide the proposed evaluations and other services? Is the investigator team interdisciplinary (i.e., including expertise in different research and evaluation methods) and do the investigators have the appropriate levels of quantitative and qualitative expertise, as well as commitment to diversity and inclusion? Does the VA, academic, and environment of interested groups in which the Center will operate contribute to the probability of success? Do the proposed evaluations benefit from unique features of the setting and partnerships, and is there evidence of institutional support? Is the Multiple PD/PI Leadership plan adequate (if applicable)? Do the MPIs have complementary and integrated expertise; are their leadership approach, governance and organizational structure appropriate for the application? Have they demonstrated an ongoing record of accomplishments in the field of evidence-based policy, implementation, QI, or evaluation science?

Feasibility (Coordinating Center only): Does the Center have a rigorous, coordinated, well-organized plan and proposed execution for the following activities?

- Review, assignment, and coordination of time sensitive evaluation requests
- Implementation of training and consultation in evaluation and implementation methods
- Coordination, management and facilitation of key ORD/QUERI capacity-building initiatives, including QUERI Rapid Response Team mechanisms, HSR&D Researchers and Evaluators in Residence, and mentoring/training opportunities for VA employees

In addition to the above criteria, the following may be considered in the determination of scientific merit and the priority score.

- Importance of the evaluation questions and overall center focus and expertise given VA priorities
- Originality, feasibility, rigor, and independence of the evaluation methods proposed
- Knowledge translation plans, including how results will inform programs, budgets, and policies in VA
- Underlying conceptual foundation, potential impact, innovation, and integration of the cores, including the potential for impact across Cores and focus areas, potential for linkages across VA operations partners and policy expertise, and potential to advance health policy methods for use in VA
- Center experience conducting partnered evaluations and completing evaluation deliverables on a specified timeline
- Potential for Center to foster career development in evidence-based policy and evaluation, implementation, and QI science, and its commitment to diversity and inclusion

Awardees will be required to submit annual and midyear reports describing QUERI Impact Measures, see the QUERI ACTION Impact Framework.

2.A. Additional Review Considerations

Quality Improvement/Protection of Human Subjects from Research Risk: Degree to which proposed work fits under the ORD Program Guide 1200.21 definition of non-research and degree to which proposed work protects human subjects from research risk.

Budget and Period of Support: The appropriateness of the proposed budget and the requested period of support may be assessed by the reviewers. The priority score should not be affected by the evaluation of the budget.

2.B. Sharing Research Data

Not applicable.

2.C. Sharing Research Resources

Not Applicable.

2.D. Disapproved Proposals

A proposal may be disapproved if the SMRB determines that the proposed study is unethical, is unlikely to yield useful information, or is not relevant to VA's mission.

- Proposals that are disapproved are not given a numerical score and may not be resubmitted.
- Studies disapproved for ethical considerations may not be carried out in VA space or with VA resources, even if the project is funded by another agency.

2.E. Appeals

The appeals process is intended to ensure that the scientific review of all proposals is fair and equitable. It is not intended as a means to resolve differences in scientific opinion between the applicant and the reviewers, to adjust funding decisions, or to circumvent the peer review process. (See VHA Handbook 1204.01)

If a PD/PI submits a revised application and an appeal of the previous application is subsequently sustained and funded before the revised application is reviewed, the revised application will be administratively withdrawn. If the revised application receives a fundable score and the appeal is sustained and fundable, only one of the two projects will be funded.

Note: Applicants are encouraged to revise and resubmit their Merit Review, if allowed, or submit a new Merit Review while an appeal is under review.

Section VI. Award Administration Information

1. Award Notices

After the peer review of the application is completed, the PD/PI (only) will be able to access his or her Final Score and Summary Statement (written critique) via the NIH eRA Commons once this information has been released by QUERI Staff. A separate notification of the review meeting outcome will be sent to the medical center director, ACOS/R&D, AO/R&D and if there is an HSR&D Center at the PI's location, to the Center Director.

If an application is not selected for funding it will remain in eRA Commons in a "pending council review" status.

The summary statement can be accessed through eRA Commons.

2. Administrative and National Policy Requirements

Research Integrity. HSR&D is committed to the highest standards for the ethical conduct of research. Maintenance of high ethical standards requires that VA medical centers and investigators applying for, and receiving, this award have appropriate procedures to preclude the occurrence of unethical research practices. All research data must be retained for 5 years after completion of a research project.

The PD/PI and others associated with the research must subscribe to accepted standards of rational experimental research design, accurate data recording, unbiased reporting of data, respect for the intellectual property of other investigators, adherence to established ethical codes, legal standards for the protection of human and animal subjects, and proper management of research funds.

Deliberate falsification or misrepresentation of research data will result in withdrawal of an application, possible suspension, or termination of an award, and potentially, suspension of the investigator's eligibility to submit proposals to HSR&D.

Acknowledging VA Research Support. By accepting this award, the PD/PI agrees to properly acknowledge VA affiliation and support in all public reports and presentations (see VHA Handbook 1200.19).

Failure to acknowledge VA affiliation and support may result in termination of the award.

Intellectual Property Rights. By accepting this award, the PD/PI agrees to comply with VA policies regarding intellectual property disclosure obligations and Federal Government ownership rights resulting from the proposed work (see VHA Handbook 1200.18).

Section VII. Agency Contacts

We encourage scientific/programmatic inquiries concerning this funding opportunity and welcome the opportunity to answer questions from potential applicants.

All questions related to the Merit Review submission (e.g., FOA/RFA, SF424, financial management, etc.) should be directed to the Scientific Merit Review Program staff (vhacoscirev@va.gov). All questions concerning electronic submission (e.g., technical issues with Grants.gov and eRA) should be directed to the eRA mailbox at rd-era@va.gov. Telephone calls and/or emails sent to individual staff may go unanswered if that staff member is out of the office.

1. Scientific/Research Contact

The PD/PI may contact the QUERI Scientific Review Officer (SRO), Melissa Braganza (vacoqueri@va.gov), with questions specifically related to scientific issues raised in the summary statement for a reviewed proposal or the scientific content of a proposal to be submitted. The Associate Chief of Staff for Research and Development (ACOS/R&D) should make all other contacts with HSR&D staff at VA central office (VACO), including questions relating to budget modifications noted in the summary statement**.** Contact information for the SROs for individual Merit Review Panels may be found at HSR&D Scientific Merit Review Board.
