# Supplementary material for: Developing Evidence to Support Policy: Protocol for the StrAtegic PoLicy EvIdence-Based Evaluation CeNTer (SALIENT)
Source: JMIR Res Protoc. 2024 Sep 19;13:e59830. doi: 10.2196/59830 (PMC11450355; doi:10.2196/59830)
Supplement: Multimedia Appendix 2 [file resprot_v13i1e59830_app2.pdf]

~~**Data resources compiled by SALIENT investigators to be used in future evaluations and scientific investigation.**~~

1) **Longitudinal Data for Post-9/11 Era Veterans developed within the Long-term Impact of Military Relevant Brain Injury Consortium: Phenotypes of Persistent Comorbidity in Post-9/11 Era Veterans with mTBI (LIMBIC; hereafter LIMBIC Phenotype study) (I01 RX003443-03)**

Table 1: Data resources for LIMBIC Phenotype Cohort.

**Data Source**	**Purpose**
DoD Trauma Registry and Theatre Data Management Store	Information on deployment related healthcare and injuries
Military Treatment Facility and Tricare inpatient, outpatient, and pharmacy care	Longitudinal information on non-deployment related care
Other Specialized examination results	Audiogram, Tympanogram, Speech Audiometry, DOEHRS-HC, Immunizations, Device Exposure, etc.
VA DoD Identity Repository (VADIR)	Provides information on military characteristics and deployments (e.g., number and duration of deployment[s]) for Post-9/11 conflicts.
VHA inpatient, outpatient and pharmacy data	Longitudinal information on VHA care including purchased care
Vital Signs	Longitudinal DoD and VHA information on height, weight, alcohol screening, pain etc.
Cost data	Longitudinal information on DoD and VHA cost of care
Pulmonary Function Tests	VA and DoD results of pulmonary function tests
Radiology Results	Radiology results for Neuroimaging will be examined for abnormal/normal results and specific abnormalities identified (DoD and VA data)
VA Comprehensive TBI Evaluation	Data compiled from the comprehensive TBI evaluation from Post-9/11 Vs who are enrolled in VA healthcare. Data include self-reported TBI/blast exposures, and are incorporated into the allsources TBI severity algorithm; Neurobehavioral Symptom Inventory scores are also included
Prosthetics	Specific prosthetics used by Veterans
Behavioral Health Measures	Longitudinal behavioral health information in VHA (DoD acquisition in progress)
Text Notes	VHA texts notes available for natural language processing to identify blast exposure (possibly subconcussive events), cognitive decline/memory issues, problems with activities of daily living/instrumental activities of daily living, social support, psychosocial stressors, etc.
COVID-19 Shared Data	Data from the COVID-19 repository identifying those who were tested, tested positive, and received inpatient/outpatient care for COVID-19 paid by VA
VA Vital Status files	The Vital Status files include date of birth and date of death acquired from the VA, DoD and Social Security sources
Other VHA Screening	Screening including but not limited to homelessness and military sexual trauma in health factors data
Veterans Benefits Administration Data	VETSNET provides information on specific service-connected disabilities and benefits received
Joint Department of Defense (DoD) and Veterans Affairs (VA) Suicide Data Repository (SDR)	Derived from the National Death Index plus data to identify death from accidents, self-harm, violence, and disease related conditions

2) **Longitudinal Data for Gulf War Era Veterans developed within Healthcare Utilization Patterns and Associated Costs for Gulf War I Era Veterans ([hereafter Gulf War study) 1I01 HX001682)**

Table 2: Data sources for Gulf War era Veteran Cohort.

Data Source	Purpose
VA DoD Identity Repository (VADIR)	Provides information on military characteristics and deployments (e.g., number and duration of deployment[s]) for Post-9/11 conflicts.
VHA inpatient, outpatient, and pharmacy data	Longitudinal information on VHA care including purchased care
Vital Signs	Longitudinal VHA information on height, weight, alcohol screening, pain etc.
Cost data	Longitudinal information on VHA cost of care
Pulmonary Function Tests	VA results of pulmonary function tests
Radiology Results	Radiology results for Neuroimaging will be examined for abnormal/normal results and specific abnormalities identified (VA data)
VA Vital Status files	The Vital Status files include date of birth and date of death acquired from the VA, DoD and Social Security sources.
Other VHA Screening	Screening including but not limited to homelessness and military sexual trauma in health factors data.
Veterans Benefits Administration Data	VETSNET provides information on specific service-connected disabilities and benefits received.
Joint Department of Defense (DoD) and Veterans Affairs (VA) Suicide Data Repository (SDR)	Derived from the National Death Index plus data to identify death from accidents, self-harm, violence, and disease related conditions.

Additional data sources identified in **Section A above** can be added as needed.

3) **Geriatric Data including Geriatric Extended Care Data Analysis Center (GECDAC) derived variables developed within the Elizabeth Dole Center of Excellence for Veteran and Caregiver Research (Hereafter Dole COE; I50 HX002767)**

Table 3: Data sources for Data and Policy Core Cohort

Data Source	Purpose
GECDAC Core Files	Characterizes patient GEC service utilization as well as patient diagnoses during the fiscal year.
VA Rx Outpatient	Includes dispensing information on adult incontinence related products.
VA Vital Status Mini	The Vital Status files include date of birth and date of death acquired from the VA, DoD and Social Security sources.
Medicare files via VIReC	Included files with service utilization relevant to long-term institutionalization: skilled and non-skilled home care, skilled nursing facilities, and hospice.

4) **Outpatient and Inpatient Behavioral Health Care in VA and VA-Purchased Community Care (SDR 18-318; MPI: Rosen, Vanneman, Wagner)**

Table 4: Data sources for Behavioral Health Care in VA and VA-Purchased Community Care study.

Data Source	Purpose
VA outpatient and inpatient behavioral health data	Longitudinal information on VA behavioral health care including purchased care. These data contain procedure information (CPT and HCPCs) as well as Veteran-level and county-level characteristics.
