# Supplementary material for: Developing Evidence to Support Policy: Protocol for the StrAtegic PoLicy EvIdence-Based Evaluation CeNTer (SALIENT)
Source: JMIR Res Protoc. 2024 Sep 19;13:e59830. doi: 10.2196/59830 (PMC11450355; doi:10.2196/59830)
Supplement: Multimedia Appendix 3 [file resprot_v13i1e59830_app3.pdf]

SALIENT team methodological expertise with selected references

**Quantitative Data Acquisition**[1–9]
Obtaining, cleaning, managing, linking, and creating longitudinal analytical data sets from diverse sources of “big data” and national survey data. **Appendix 1** lists data resources developed by SALIENT investigators.<br>**(Bouldin, Cochran, French, A. Jones, Mohanty, Nelson, Pugh, Samore, Smith, Vanneman)**
**Quantitative Methods**[5–7,7–20]
Objective Measurement and numerical study of data from various sources using computational techniques from disciplines including but not limited to: longitudinal, secondary data analysis, Bayesian analysis, latent class/transition analysis, Markov models, causal inference, hierarchical models, multilevel and stepped-wedge analysis, epidemiology.<br>**(Bouldin, Burningham, Cochran, French, A. Jones, Kean, Kennedy, Mohanty, Nelson, Pugh, Presson, Smith, Vanneman)**
**Economic Methods/Analysis**[21–25]
Designing and conducting health economic evaluations of programs and interventions by assessing healthcare resource utilization alone—through cost analyses and budget impact analyses—or in conjunction with health outcomes—though cost-effectiveness analyses and return on investment assessments.<br>**(French, Maloney, Nelson, Smith, Vanneman, Waitzman, Wilson)**
**Informatics & Network Science**[4,20,21,26–35]
Machine learning/artificial intelligence, simulation models, network analysis, creation of decision support tools and dashboards<br>**(Burningham, Butler, Haun, M. Jones, Kennedy, Pugh, Samore, Valente, Vega Yon, White)**
**Survey and Ecological Momentary Assessment Methods**[19,27,36,37]
Designing, administering, and analyzing surveys and ecological momentary assessment data of Veterans, caregivers, and non- veteran comparison groups for HSR&D, QUERI and DoD funded projects.<br>**(Bouldin, Fagerlin , A. Jones, Kalvesmaki, Knight, Millar, Potter, Pugh, Vanneman, Wetter)**
**Qualitative Data Collection/Analysis**[19,26,36,38–44]
**Collecting** qualitative data (open-ended or semi-structured interviews or written communication, observation, focus groups, document review, ethnography, participatory action research). **Analyses**: e.g., grounded theory, narrative synthesis, phenomenology, rapid templated analysis, rapid ethnography, periodic reflection, Integrating NLP & qualitative analysis; **Mixed Methods***:* Integrating the collection, analyses, & presentation of qualitative and quantitative data using concurrent, explanatory, and exploratory designs.<br>**(Bouldin, Butler, Fagerlin, Haun, Kalvesmaki, Knight, Naranjo, Smith, Vanneman, Zickmund)**
**Implementation & Quality Improvement Science**[26,27,39–41,45–49]
Projects with operational partners include antibiotic stewardship, clinical models for disease-specific treatment (chronic multi-symptom illness/environmental exposure, addiction, traumatic brain injury, neurological conditions), homelessness, & decision-aid tools.<br>**(Butler, Cochran, Gordon, Haun, Kean, Leykum, Lowery, McAndrew, Naranjo, Pugh, Rubin, Schlechter, Smith)**
**Operations Research and Program Evaluation**[9,25–27,38,41,43,44,48,50–56,56]
Work with operational/clinical partners to evaluate clinical programs, institutional and governing policies, and practices. Program evaluation using formative and summative techniques inform operational partners throughout the development, implementation, and scaling of various programs and operations in clinical and non-clinical settings.<br>**(Bouldin, Cochran, Haun, Kalvesmaki, Hunt, Kean, McAndrew, McMahon-Grenz, Pugh, Smith, Vanneman, Zickmund)**
**Knowledge Translation**[50]
Communication and dissemination of findings via user centered design (e.g., playbooks, policy briefs, infographics, toolkits)<br>**(Benzinger, Fagerlin, Haun, Kean, McMahon-Grenz, Rathunde, Smith, White)**
**Policy Analysis**[8,17,19,23,25,42,52]
Policy process evaluation, cost-benefit analyses, policy implementation processes, policy implementation facilitators, policy goal analyses, multi-policy network analyses.<br>**(French, Haun, Kalvesmaki, Leykum, Nelson, Rathunde, Vanneman, White)**
**Clinical Trials**[55,57]
Cluster randomized trials, pragmatic trials, stepped wedge design, hybrid designs<br>**(Cochran, French, Gordon, Haun, McAndrew, Presson)**

Underlined names identify lead within method.

References

1. Mohanty AF, Helmer DA, Muthukutty A, et al. Fibromyalgia syndrome care of Iraq-and Afghanistan-deployed veterans in Veterans Health Administration. Published online 2016.

2. Mohanty AF, McAndrew LM, Helmer D, Samore MH, Gundlapalli AV. Chronic multisymptom illness among Iraq/Afghanistan-deployed US veterans and their healthcare utilization within the veterans health administration. *Journal of general internal medicine*. 2018;33:1419-1422.

3. Pugh MJ, Jaramillo CA, Leung K wei, et al. Increasing prevalence of chronic lung disease in veterans of the wars in Iraq and Afghanistan. *Military medicine*. 2016;181(5):476-481.

4. Pugh MJ, Finley EP, Wang CP, et al. A retrospective cohort study of comorbidity trajectories associated with traumatic brain injury in veterans of the Iraq and Afghanistan wars. *Brain injury*. 2016;30(12):1481-1490.

5. Pugh JA, Wang C, Espinoza SE, et al. Influence of frailty-related diagnoses, high-risk prescribing in elderly adults, and primary care use on readmissions in fewer than 30 days for veterans aged 65 and older. *Journal of the American Geriatrics Society*. 2014;62(2):291-298.

6. Jones BE, Ying J, Nevers MR, et al. Trends in Illness Severity, Hospitalization, and Mortality for Community-Onset Pneumonia at 118 US Veterans Affairs Medical Centers. *Journal of General Internal Medicine*. 2022;37(15):3839-3847.

7. Vanneman ME, Harris AH, Chen C, Adams RS, Williams TV, Larson MJ. Postdeployment behavioral health screens and linkage to the veterans health administration for army reserve component members. *Psychiatric services*. 2017;68(8):803-809.

8. Vanneman ME, Phibbs CS, Dally SK, Trivedi AN, Yoon J. The Impact of Medicaid Enrollment on Veterans Health Administration Enrollees' Behavioral Health Services Use. *Health Services Research*. 2018;53:5238-5259.

9. Vanneman ME, Harris AH, Asch SM, Scott WJ, Murrell SS, Wagner TH. Iraq and Afghanistan veterans' use of Veterans Health Administration and purchased care before and after Veterans Choice Program implementation. *Medical care*. 2017;55(7):S37-S44.

10. Buys MJ, Bayless K, Romesser J, et al. Opioid use among veterans undergoing major joint surgery managed by a multidisciplinary transitional pain service. *Regional Anesthesia & Pain Medicine*. 2020;45(11):847-852.

11. Samore MH, Looney A, Orleans B, et al. Probability-based estimates of severe acute respiratory syndrome coronavirus 2 seroprevalence and detection fraction, Utah, USA. *Emerging Infectious Diseases*. 2021;27(11):2786.

12. Evans ME, Simbartl LA, Kralovic SM, et al. Healthcare-associated infections in Veterans Affairs acute-care and long-term healthcare facilities during the coronavirus disease 2019 (COVID-19) pandemic. *Infection Control & Hospital Epidemiology*. 2023;44(3):420-426.

13. Lynch KE, Viernes B, Gatsby E, et al. Positive predictive value of COVID-19 ICD-10 diagnosis codes across calendar time and clinical setting. *Clinical Epidemiology*. Published online 2021:1011-1018.

14. Peterson KS, Lewis J, Patterson OV, et al. Automated Travel History Extraction From Clinical Notes for Informing the Detection of Emergent Infectious Disease Events: Algorithm Development and Validation. *JMIR public health and surveillance*. 2021;7(3):e26719.

15. Gamage SD, Evans ME, McCauley BP, et al. Hospital coronavirus disease 2019 (COVID-19) public health reporting: Lessons from validation of an automated surveillance tool to facilitate data collection. *Infection Control & Hospital Epidemiology*. 2023;44(5):802-804.

16. Gawron AJ, Kaltenbach T, Dominitz JA. The impact of the coronavirus disease-19 pandemic on access to endoscopy procedures in the VA healthcare system. *Gastroenterology*. 2020;159(4):1216-1220.

17. Harris AH, Beilstein-Wedel EE, Rosen AK, et al. Comparing Complication Rates After Elective Total Knee Arthroplasty Delivered Or Purchased By The VA: Study examines elective total knee arthroplasty delivered by or purchased by the Department of Veterans Affairs. *Health Affairs*. 2021;40(8):1312-1320.

18. Rosen AK, Wagner TH, Pettey WB, et al. Differences in risk scores of Veterans receiving community care purchased by the Veterans Health Administration. *Health services research*. 2018;53:5438-5454.

19. Vanneman ME, Wagner TH, Shwartz M, et al. Veterans' Experiences With Outpatient Care: Comparing The Veterans Affairs System With Community-Based Care: Study compares veterans' experiences in VA-delivered and community-based outpatient care. *Health Affairs*. 2020;39(8):1368-1376.

20. Pugh MJ, Swan AA, Amuan ME, et al. Deployment, suicide, and overdose among comorbidity phenotypes following mild traumatic brain injury: a retrospective cohort study from the Chronic Effects of Neurotrauma Consortium. *PLoS One*. 2019;14(9):e0222674.

21. Haun JN, Alman AC, Melillo C, et al. Using electronic data collection platforms to assess complementary and integrative health patient-reported outcomes: feasibility project. *JMIR Medical Informatics*. 2020;8(6):e15609.

22. Nelson RE, Suo Y, Pettey W, et al. Costs associated with health care services accessed through VA and in the community through Medicare for veterans experiencing homelessness. *Health Services Research*. 2018;53:5352-5374.

23. Nelson RE, Montgomery AE, Suo Y, et al. Temporary Financial Assistance Decreased Health Care Costs For Veterans Experiencing Housing Instability: Study examines temporary financial assistance and health care costs for veterans experiencing housing instability. *Health Affairs*. 2021;40(5):820-828.

24. Nelson RE, Goto M, Samore MH, et al. Expanding an economic evaluation of the Veterans Affairs (VA) methicillin-resistant Staphylococcus aureus (MRSA) prevention initiative to include prevention of infections from other pathogens. *Clinical Infectious Diseases*. 2021;72(Supplement_1):S50-S58.

25. Nelson RE, Hicken B, Vanneman M, Liu CF, Rupper R. The impact of a change in the price of VA health care on utilization of VA and Medicare services. *Medical Care*. 2018;56(7):569-576.

26. Haun JN, Chavez M, Nazi KM, Antinori N. Developing a Health Information Technology Systems Matrix: A Qualitative Participatory Approach. *Journal of Medical Internet Research*. 2016;18(10):e6499. doi:10.2196/jmir.6499

27. Haun JN, Patel NR, Lind JD, Antinori N. Large-scale survey findings inform patients' experiences in using secure messaging to engage in patient-provider communication and self-care management: a quantitative assessment. *Journal of medical Internet research*. 2015;17(12):e5152. doi:10.2196/jmir.5152

28. Rosen AK, Vanneman ME, O'Brien WJ, et al. Comparing cataract surgery complication rates in veterans receiving VA and community care. *Health Services Research*. 2020;55(5):690-700.

29. Goodrich D, Miake-Lye I, Braganza M, Wawrin N, Kilbourne A. The QUERI Roadmap for Implementation and Quality Improvemen. Published online 2020. Accessed October 17, 2023. https://www.ncbi.nlm.nih.gov/books/NBK566223

30. Braganza MZ, Kilbourne AM. The quality enhancement research initiative (QUERI) impact framework: measuring the real-world impact of implementation science. *Journal of General Internal Medicine*. 2021;36:396-403.

31. Kilbourne AM, Glasgow RE, Chambers DA. What can implementation science do for you? Key success stories from the field. *Journal of General Internal Medicine*. 2020;35:783-787.

32. Straus SE, Tetroe J, Graham I. Defining knowledge translation. *Cmaj*. 2009;181(3-4):165-168.

33. Braganza MZ, Pearson E, Avila CJ, Zlowe D, Øvretveit J, Kilbourne AM. Aligning quality improvement efforts and policy goals in a national integrated health system. *Health Services Research*. 2022;57:9-19.

34. Smith JD, Li DH, Rafferty MR. The implementation research logic model: a method for planning, executing, reporting, and synthesizing implementation projects. *Implementation Science*. 2020;15(1):1-12.

35. Pugh MJV, Finley EP, Copeland LA, et al. Complex comorbidity clusters in OEF/OIF veterans: the polytrauma clinical triad and beyond. *Medical care*. Published online 2014:172-181.

36. Kalvesmaki AF, Gonzales E, George RT, Nguyen H, Pugh MJ. Post-9/11 veterans perceptions of the pandemic: Areas of greatest impact on health and well-being. *Pec Innovation*. 2022;1:100096.

37. Pugh MJ, Swan AA, Carlson KF, et al. Traumatic brain injury severity, comorbidity, social support, family functioning, and community reintegration among veterans of the Afghanistan and Iraq wars. *Archives of physical medicine and rehabilitation*. 2018;99(2):S40-S49.

38. Haun JN, Lind JD, Shimada SL, et al. Evaluating User Experiences of the Secure Messaging Tool on the Veterans Affairs' Patient Portal System. *Journal of Medical Internet Research*. 2014;16(3):e2976. doi:10.2196/jmir.2976

39. Haun JN, Cotner BA, Melillo C, et al. Proactive integrated virtual healthcare resource use in primary care. *BMC health services research*. 2021;21(1):1-14.

40. Haun JN, Hathaway W, Chavez M, et al. Clinical practice informs secure messaging benefits and best practices. *Applied Clinical Informatics*. 2017;8(04):1003-1011.

41. Haun JN, Nazi KM, Chavez M, et al. A Participatory Approach to Designing and Enhancing Integrated Health Information Technology Systems for Veterans: Protocol. *JMIR Research Protocols*. 2015;4(1):e3815. doi:10.2196/resprot.3815

42. Vanneman ME, Snowden LR. Linking the legislative process to the consequences of realigning California's public mental health system. *Administration and Policy in Mental Health and Mental Health Services Research*. 2015;42:593-605.

43. Haun JN, Chavez M, Nazi K, et al. Veterans' Preferences for Exchanging Information Using Veterans Affairs Health Information Technologies: Focus Group Results and Modeling Simulations. *Journal of Medical Internet Research*. 2017;19(10):e8614. doi:10.2196/jmir.8614

44. McAndrew LM, Khatib LA, Sullivan NL, Winograd DM, Kolar SK, Santos SL. Healthcare providers' perceived learning needs and barriers to providing care for chronic multisymptom illness and environmental exposure concerns. *Life Sciences*. 2021;284:119757.

45. Gordon AJ, Drexler K, Hawkins EJ, et al. Article Commentary: Stepped Care for Opioid Use Disorder Train the Trainer (Scoutt) Initiative: Expanding Access to Medication Treatment for Opioid Use Disorder within Veterans Health Administration Facilities. *Substance Abuse*. 2020;41(3):275-282.

46. Hawkins EJ, Danner AN, Malte CA, et al. Clinical leaders and providers' perspectives on delivering medications for the treatment of opioid use disorder in Veteran Affairs' facilities. *Addiction Science & Clinical Practice*. 2021;16(1):1-12.

47. Jones AL, Kelley AT, Suo Y, et al. Trends in health service utilization after enrollment in an interdisciplinary primary care clinic for veterans with addiction, social determinants of health, or other vulnerabilities. *Journal of General Internal Medicine*. 2023;38(1):12-20.

48. Hawkins EJ, Malte CA, Gordon AJ, et al. Accessibility to medication for opioid use disorder after interventions to improve prescribing among nonaddiction clinics in the US veterans health care system. *JAMA Network Open*. 2021;4(12):e2137238-e2137238.

49. Lo-Ciganic W, Gellad WF, Gordon AJ, et al. Association between trajectories of buprenorphine treatment and emergency department and in-patient utilization. *Addiction*. 2016;111(5):892-902.

50. Haun J, Noland-Dodd V, Varnes J, Graham-Pole J, Rienzo B, Donaldson P. Testing the BRIEF health literacy screening tool. *Fed Pract*. 2009;26(12):24-31.

51. Haun JN, Tomoyasu N, Paolicelli C, Melillo C, Atkins D. High-Risk, High-Impact Health Services Research: Developing an Innovation Initiative. *Studies*. 2020;6(1):171-177.

52. Mengeling MA, Mattocks KM, Hynes DM, Vanneman ME, Matthews KL, Rosen AK. Partnership forum: the role of research in the transformation of veterans affairs community care. *Medical care*. 2021;59(6 Suppl 3):S232.

53. Bloeser K, McCarron KK, Merker VL, et al. "Because the country, it seems though, has turned their back on me": experiences of institutional betrayal among veterans living with Gulf War illness. *Social Science & Medicine*. 2021;284:114211.

54. Valenstein-Mah H, Hagedorn H, Kay CL, Christopher ML, Gordon AJ. Underutilization of the current clinical capacity to provide buprenorphine treatment for opioid use disorders within the Veterans Health Administration. *Substance abuse*. 2018;39(3):286-288.

55. Hagedorn HJ, Gustavson AM, Ackland PE, et al. Advancing pharmacological treatments for opioid use disorder (ADaPT-OUD): an implementation trial in eight veterans health administration facilities. *Journal of general internal medicine*. 2022;37(14):3594-3602.

56. Gordon AJ, Kavanagh G, Krumm M, et al. Facilitators and barriers in implementing buprenorphine in the Veterans Health Administration. *Psychology of Addictive Behaviors*. 2011;25(2):215.

57. Schneider AH, Bair MJ, Helmer DA, et al. Protocol for a type 1 hybrid effectiveness/implementation clinical trial of collaborative specialty care for Veterans with Gulf War Illness. *Life Sciences*. 2022;292:120004.
