# Supplementary material for: Developing Evidence to Support Policy: Protocol for the StrAtegic PoLicy EvIdence-Based Evaluation CeNTer (SALIENT)
Source: JMIR Res Protoc. 2024 Sep 19;13:e59830. doi: 10.2196/59830 (PMC11450355; doi:10.2196/59830)

PARTNERED EVALUATION INITIATIVE

Characterization, evaluation & implementation of innovative TBI intensive evaluation and treatment program (IETP)

Playbook Overview

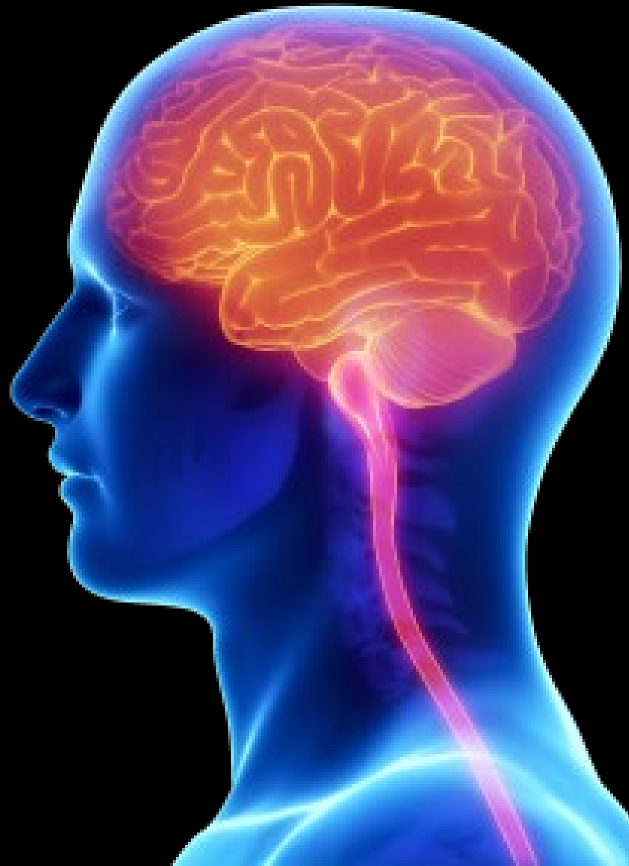

The following pages outline the playbook blueprint for this project.

May 2021

Table of Contents

2
Table of Contents
Presentation Structure

3
Literature Review
Background Information

6
Playbook Components
Development of Sections 1, 2 & 3

16
Project Timeline
Project Deliverable Activities

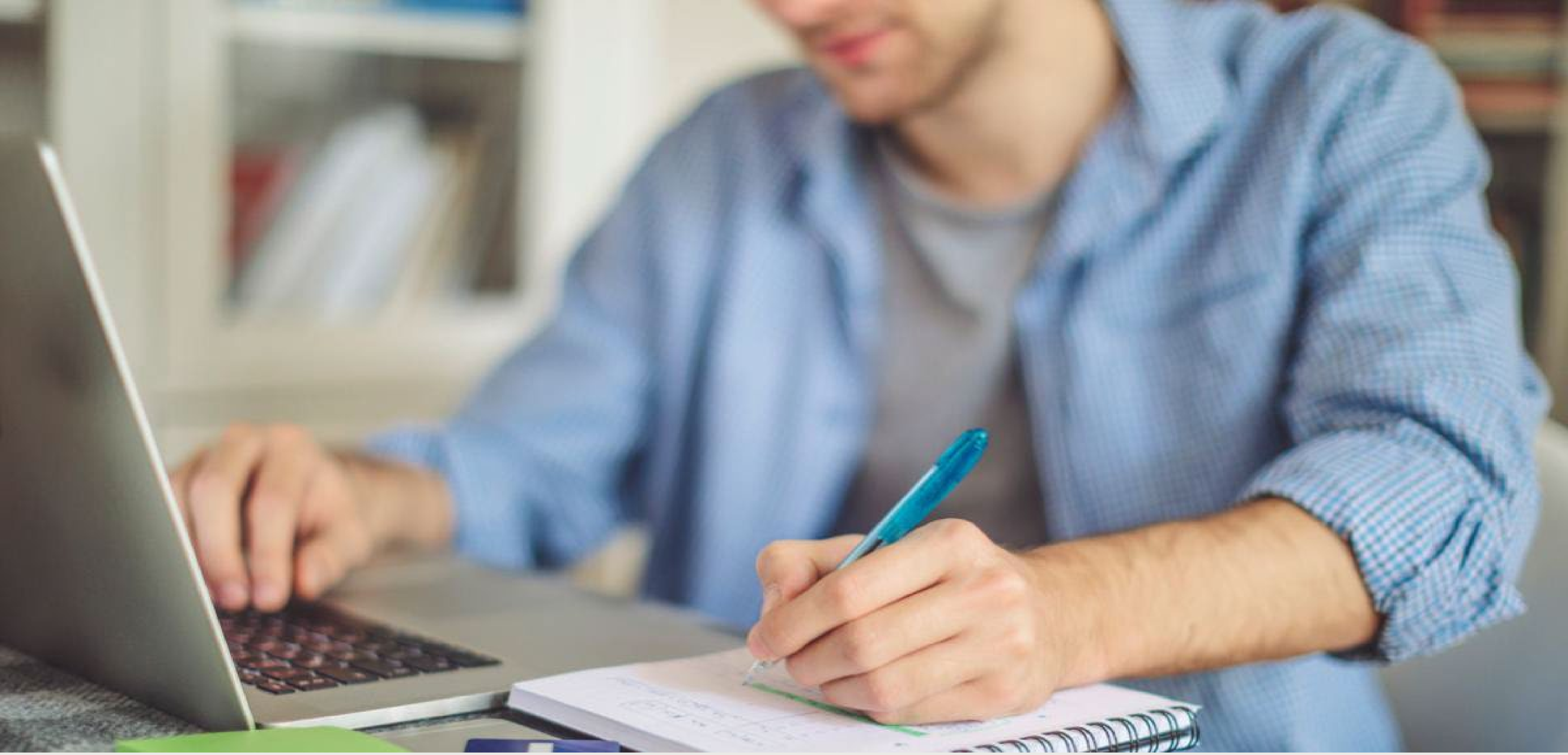

GETTING STARTED
LITERATURE REVIEW

Research & Development: Literature Review

To determine the background components, a literature review was executed and the determinates from research yielded that the who, what, when, where, why, and how should be thoroughly described in the background section of the playbook.

Enhancing the Impact of Implementation Strategies in Healthcare: A Research Agenda

Introduction: Nearly 20 years ago, Grol and Grimshaw asserted that evidence-based practice must be complemented by evidence-based implementation. This article reviewed process in implementation science and suggested five priorities for enhancing the impact of implementation strategies, specifically among healthcare.

Five Priorities for Research on Implementation Strategies:

1. Enhance methods for designing and tailoring implementation strategies
2. Specify and test mechanisms of change
3. Conduct more effectiveness research on discrete, multi-faceted, and tailored implementation strategies
4. Increase economic evaluations of implementation strategies
5. Improve tracking and reporting of implementation strategies

Conclusion: Implementation science has advanced throughout the years with a stronger understanding of implementation strategies. There are several resources that can inform implementation strategies, which include: established taxonomies of implementation strategies and behavior change techniques, repositories of systematic reviews, methods for selecting and tailoring implementation strategies, and reporting guidelines that promote replicability.

Applying this literature to PEI TBI IETP:

Using the five priorities outlined for research implementation strategies, the research team will use intervention mapping to design a strategy that outlines the innovative modality for delivering evidence-based care in a residential, inpatient format (IETP). The research team will assess determining factors that may affect the mechanisms of change at an organizational structure to implement IETP at the five PRCs. The implementation strategy will be developed using feedback gathered from Aims 1&2, which will detail the unique operational process at each PRC site. Sustainability will be based upon ongoing evaluation of the implementation strategies at each site. The implementation tracking procedures will determine the next steps for continued effectiveness of the overall program.

Powell BJ, Fernandez ME, Williams NJ, Aarons GA, Beidas RS, Lewis CC, McHugh SM and Weiner BJ (2019) Enhancing the Impact of Implementation Strategies in Healthcare: A Research Agenda. Front. Public Health 7:3. doi: 10.3389/fpubh.2019.00003

Research & Development: Literature Review

The IRLM uses data on inputs, activities, and outcomes to specify the relationships between determinants of implementation, implementation strategies, and the mechanistic links between strategies and outcomes. The IRLM is contextualized by CFIR constructs.

The Implementation Research Logic Model: a method for planning, executing, reporting, and synthesizing implementation projects

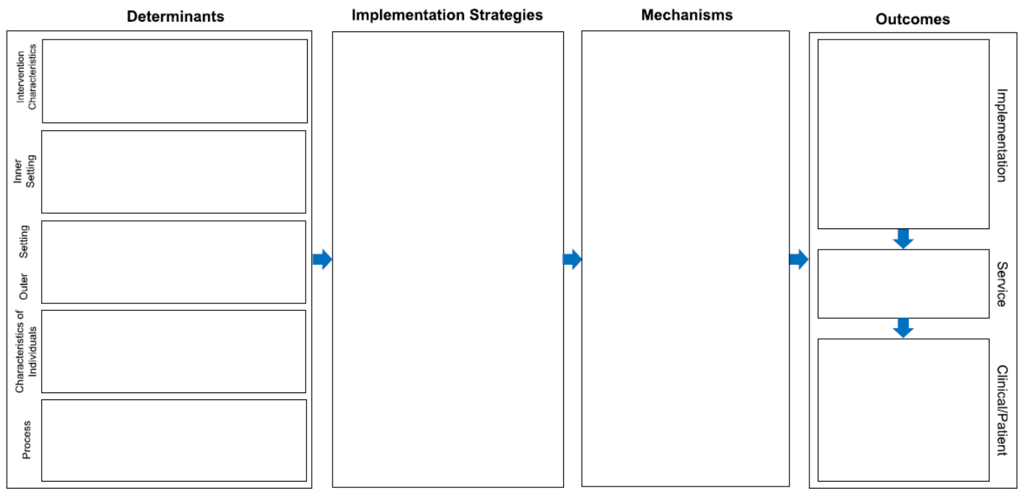

Fig. 1 Implementation Research Logic Model (IRLM) Standard Form. *Notes.* Domain names in the determinants section were drawn from the Consolidated Framework for Implementation Research. The format of the outcomes column is from Proctor et al. 2011

Introduction: The Implementation Research Logic Model (IRLM) provides justification -- drawing from various models, frameworks, and theories -- for the determinants, strategies, and outcomes of proposed change initiatives as they relate to one another for improving the adoption of evidence-based interventions in healthcare delivery systems.

Conclusion: The IRLM is a structure with guiding principals that serves as a roadmap for how a project is to be carried out and clearly specifies how the project is to be conducted using the connections between determinants, strategies, mechanisms, and outcomes for the project.

Applying this literature to PEI TBI IETP:

Using team expertise and literature of evidenced based implementation strategies that are relevant to the IETP clinical components reviewed in IETP Characterization Table, the process of implementation to date at PRCs will be represented as a CFIR based Implementation Research Logic Model (IRLM). The IRLM will use data on inputs, activities, and outcomes to specify the relationships between determinants of implementation, implementation strategies, and the mechanistic links between strategies and outcomes. The IRLM, contextualized by CFIR constructs, will be triangulated, and analyzed as a part of Aim 1 and iteratively revised to reflect clinical relevance for each PRC.

Smith J, Li D, Rafferty M. The Implementation Research Logic Model: a method for planning, executing, reporting, and synthesizing implementation projects. *Implementation Science*. 2020;15(1). doi:10.1186/s13012-020-01041-8

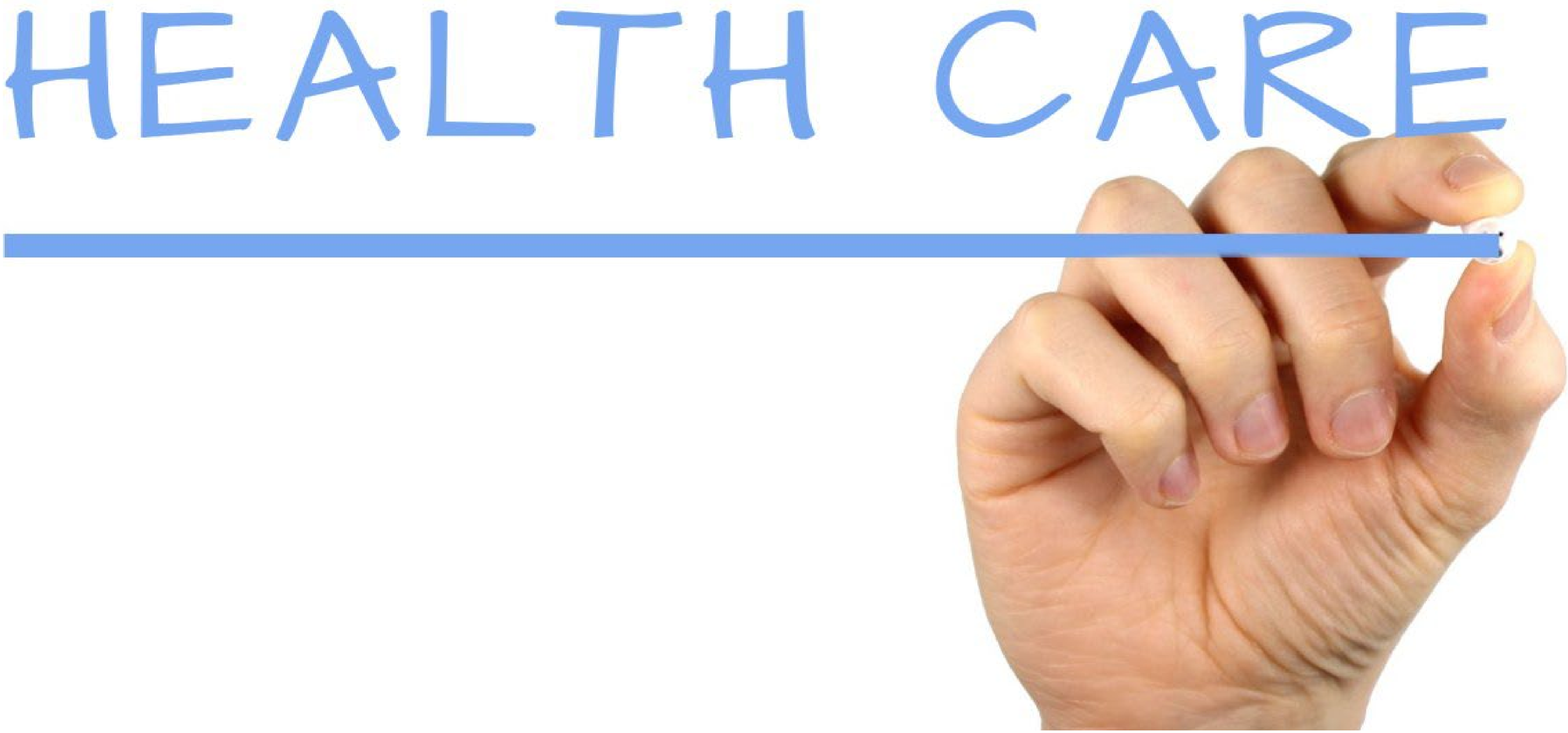

PLAYBOOK COMPONENTS

AIMS 1, 2 & 3

Playbook Components

The TBI Intensive Evaluation and Treatment Program (IETP), is an innovative modality for delivering evidence-based care in a residential, inpatient format. The PM&R National Program Office will facilitate the implementation of the high consumer demand IETP at a single site to all VA PRCs: ***Tampa, Palo Alto, Minneapolis, Richmond), and San Antonio****. Ineffective treatment components will be targeted for de-implementation.*

The playbook will outline the following:

Section 1 – Background

- Stakeholders Organizational Chart
- Definitions
- Project Background, Purpose & Justification

Section 2 – Data Collection & Findings

- Aim 1 Collection, Findings & Deliverables
- Aim 2 Collection, Findings & Deliverables

Section 3 – Implementation Plan

- Tailored implementation/de-implementation strategy for each site
- Informative & Educational Products

Playbook Contents

Section 1	Section 2	Section 3
Background	Data Collection & Findings	Implementation/ De-Implementation Plan

Executive Summary

The executive summary will highlight various aspects of the project at a high level to provide background information and context.

Executive summary components will include:

1. **Synopsis of Project Background**
2. **Description of Project aims, Phases of Implementation & timeline For Each PRC**
3. **Outline of the Data Collection Methods (Aim 1)**
    - Site Visit Observations
    - Key Informant Interviews
    - Focus Groups
    - Follow-up Interviews
4. **Outline of Data Collection Methods (Aim 2)**
    - Obtain Identifiers
    - Obtain Intake/Discharge Measures
    - Chart Abstraction Fidelity Assessment
    - Conduct Survey
5. **Results & Analysis of Data Collection (Aim 1 & Aim 2)**
    - Characterization of IETP Participants
    - Identified Treatment Received During IETP
    - Identified Components of IETP Associated with Sustainment/Improvement Trajectories
6. **Implementation, Feedback & Sustainability Strategy**

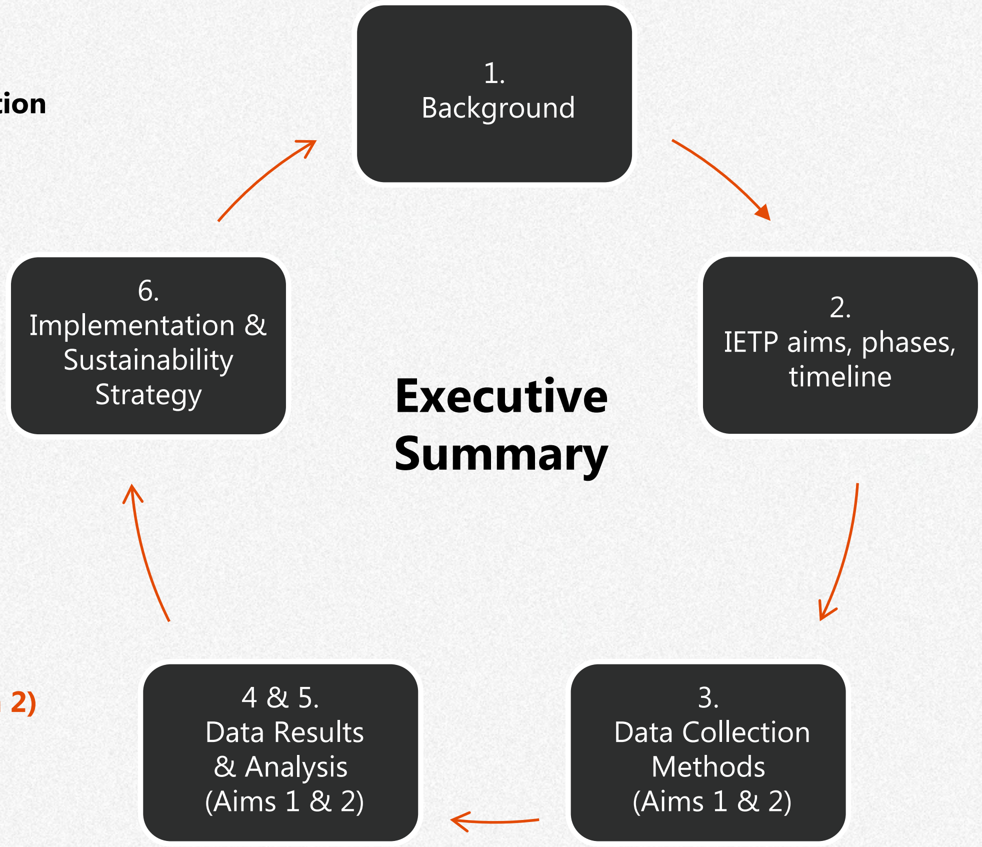

Background Content

Background components will include:

1. **Organizational Chart**
2. **Definitions**
   - Acronyms
   - Phrases
3. **Project Background, Purpose, Justification, and Playbook Use**

Interdisciplinary traumatic brain injury rehabilitation is intended to enhance functioning, reduce disability, and improve quality of life. *Record numbers of military personnel with TBI have flooded the VA and DoD during the past two decades. Since 2000, 430,720 service members have been diagnosed with TBI; the majority (82.4%) have been mild TBI* . To meet the demand for clinical services required by individuals with TBI, the Veterans Health Administration (**VHA**) Physical Medicine and Rehabilitation (**PM&R**) National Program Office developed five specialty Polytrauma Rehabilitation Centers (**PRCs**) to provide a coordinated approach to comprehensive rehabilitation including mild TBI.

What is this playbook?

The playbook is a tool that can be used at sites to strategize the implementation of effective IETP programmatic practices and de-implementation of low-value care and a resource that will include education about research implementation to support knowledge translation.

Who is this playbook for?

The playbook is tailored toward clinical and administrative IETP stakeholders across all five PRC sites: Tampa, Palo Alto, Minneapolis, Richmond), and San Antonio

Why should this playbook be used?

The playbook will provide the template training strategy for implementing IETP evidence-based practices across all five PRC sites while ineffective treatment components will be targeted for de-implementation.

How should this playbook be used?

The playbook should be used by each individual PRC site as a tailored training resource and toolkit for implementing IETP evidence-based practices for the PRC site.

When should this playbook be used?

The playbook should be referenced throughout all phases of the individual PRC site IETP implementation program and as needed for reference.

Where can this playbook be used?

This playbook is intended to be used in a residential, inpatient format (e.g., inpatient, outpatient) by each of the PRC sites for the identified specialty care modalities involved.

Data Collection Process & Timeline

The project officially kicked off with the Operational & clinical partnership to finalize deliverables. Concurrently, the aim 2 data acquisition was executed with: TBI-RCM participants from Sites, TBIMS, and DoD-VA data (DaVINCI)

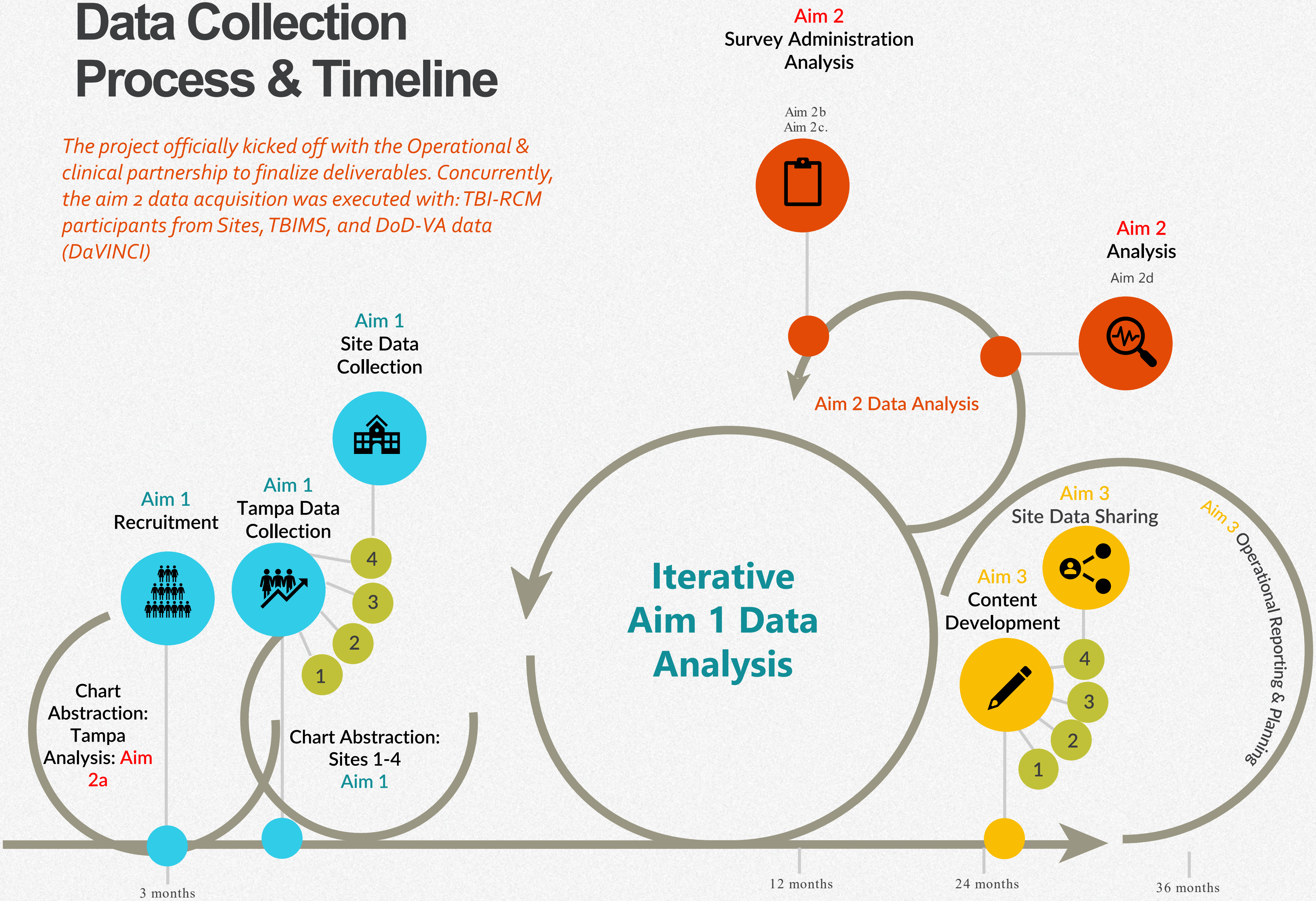

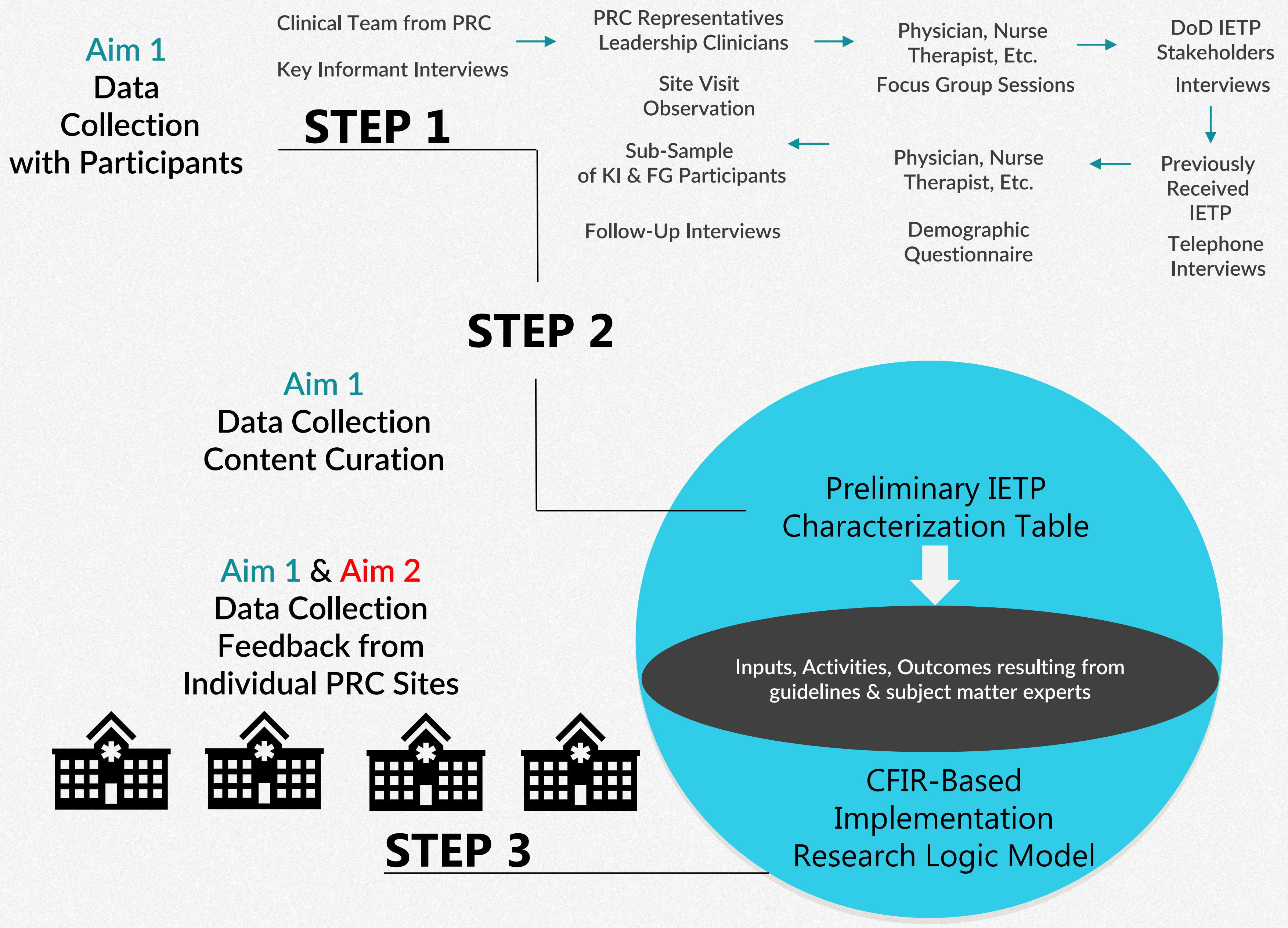
Aim 1
Data Collection with Participants
Clinical Team from PRC
Key Informant Interviews
STEP 1
PRC Representatives Leadership Clinicians
Site Visit Observation
Physician, Nurse Therapist, Etc.
Focus Group Sessions
DoD IETP Stakeholders
Interviews
Previously Received IETP
Telephone Interviews
Physician, Nurse Therapist, Etc.
Demographic Questionnaire
Sub-Sample of KI & FG Participants
Follow-Up Interviews
STEP 2
Aim 1
Data Collection Content Curation
Preliminary IETP Characterization Table
Inputs, Activities, Outcomes resulting from guidelines & subject matter experts
CFIR-Based Implementation Research Logic Model
Aim 1 & Aim 2
Data Collection Feedback from Individual PRC Sites
STEP 3

The Implementation Strategy

Implementation components will include:

1. **IETP Implementation & De-Implementation Process**

2. **Using Materials to Successfully Execute the Workflow**
   - Educational deliverables
   - Worksheets & checklists

3. **Implementation Evaluation Process**

DRAFT IN PROGRESS

Tailor Standard Operational Procedures

Reference & Interactive Materials

Submit Revisions Using Feedback Loop

Establish & Track Performance Metrics

Revise Standard Operational Procedures

Document Outcomes

Playbook Components

Sections 2 & 3

Aim 2 IETP Participant Care Continuum Process

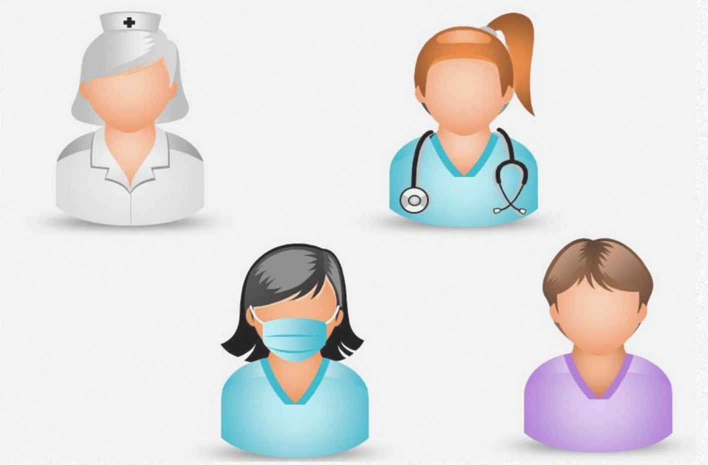

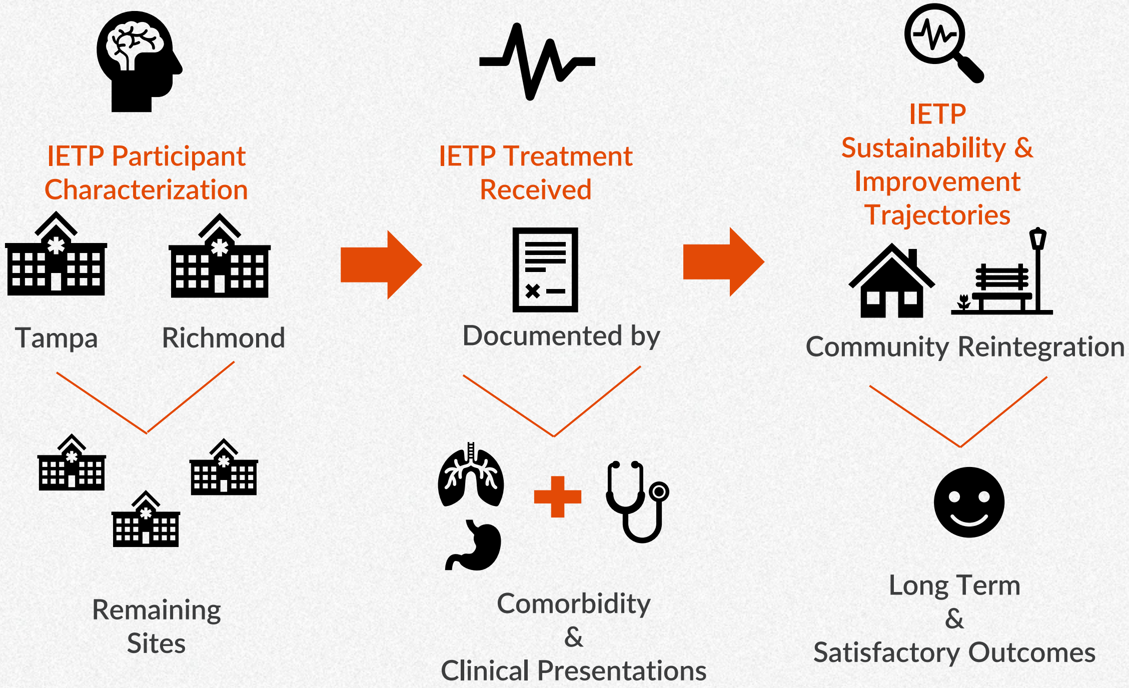

Characterization of the Core IETP Components Packaged for the Aim 3 Deliverable Playbook

Aim 1 Deliverables

- IETP Characterization Table
- Implementation Research Logic Model (IRLM)
- IETP Care Implementation Elements Inventory

Aim 2 Deliverables

- Data Summaries
- IETP Participant Identification Content
- Documented Intake & Discharge Measures
- Documented Sustainment & Improvement Trajectories

Aim 3 Deliverables

- PRC Site-Specific Implementation & De-Implementation Instructional Material
- Educational Content
- Templates & Presentations Tailored for Each PRC Site

Aim 1 Deliverables		
Deliverable Item	***Purpose of Deliverable***	***Primary Audience***
IETP Characterization Table	Background Information Organization	PRC Site Partners' Clinical Team Members
Implementation Research Model	Determine the Process of Implementation	PRC Sites' Team Members (Administrators)
IETP Care Implementation Elements Inventory	Define & Evaluate IETP Program (Plausibility Check)	PRC Sites' IETP Participants (Veterans & Providers)

Aim 2 Deliverables		
Deliverable Item	***Purpose of Deliverable***	***Primary Audience***
IETP Particiant Identification Crosswalk Database Content Extraction to Determine Key Characteristics *(Informs Database Creation)*	Patient Background Information	PRC Sites' IETP Participants (Veterans)
Intake & Discharge Measures Documented to Identify Treatment Received & Determine Trajectories of Recovery *(Informs Survey Creation)*	Patient Experience Feedback	PRC Sites' IETP Participants (Veterans)
Identification of Components of IETP Associated with Sustainment & Improvement Trajectories *(Informs Survey Creation)*	Fidelity Assessment	Operational & Stakeholder Partners

Aim 3 Playbook Deliverable (To Include all components from Aims 1 & 2 Plus):		
Deliverable Item	***Purpose of Deliverable***	***Primary Audience***
PRC Site-Specific Implementation & De-Implementation Instructional Material *(Templates, Checklists, Staffing Plan, Impacts, Sustainability Vision, Etc.)*	Tailored Implementation Strategy	PRC Sites (Entire Organizational Structure)
Educational Content (Brochures, Flyers, Promotional Material, Etc.)	Informative/Educational	PRC Sites' IETP Participants (Veterans & Providers)
PRC Site-Specific Templates & Presentation Materials	Tailored Presentation Material	PRC Sites (Entire Organizational Structure)

Design the Playbook Layout for Aim 3

The playbook design must encompass:

1. **Written Content**
   - Background information
   - Instructions
   - Fillable sheets & checklists
2. **Visuals**
   - Charts
   - Graphs
   - Tables
   - Images
3. **Live Weblinks**
   - Videos
   - Topic-specific guides
   - Handouts
   - FAQ sheets
   - Presentation materials
   - Etc.

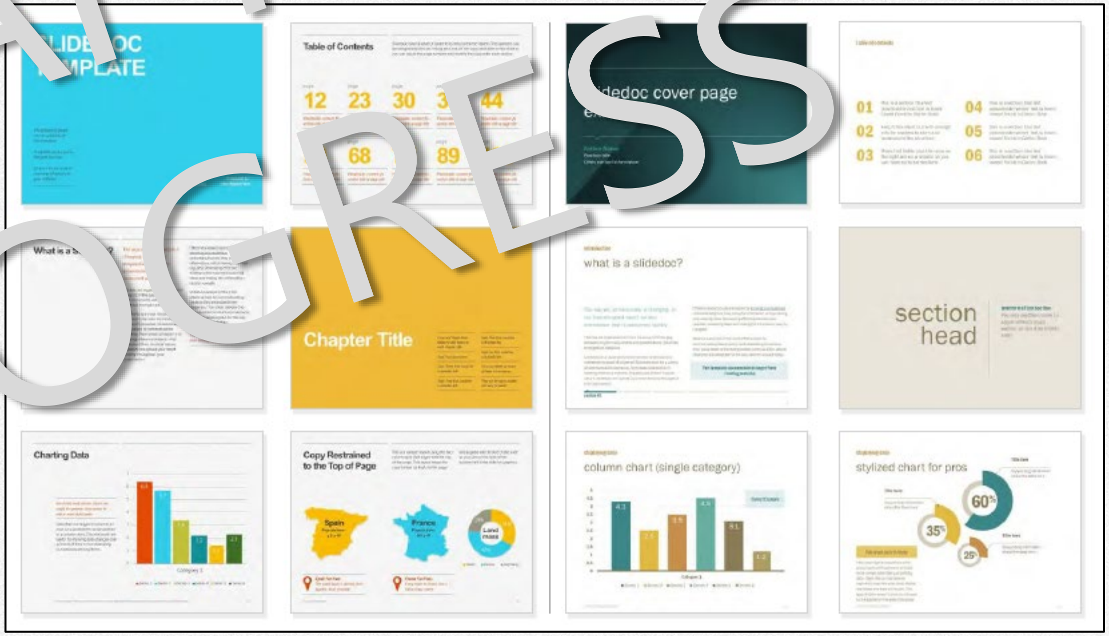

DRAFT IN PROGRESS

Design Possibilities

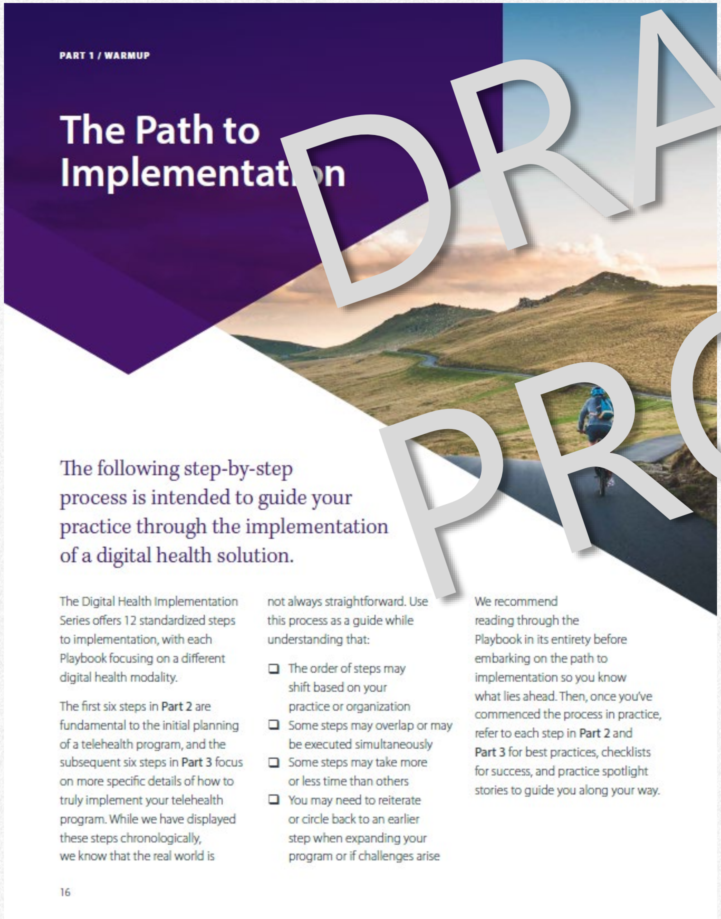

PART 1 / WARMUP

The Path to Implementat[illegible]n

The following step-by-step process is intended to guide your practice through the implementation of a digital health solution.

The Digital Health Implementation Series offers 12 standardized steps to implementation, with each Playbook focusing on a different digital health modality.

The first six steps in **Part 2** are fundamental to the initial planning of a telehealth program, and the subsequent six steps in **Part 3** focus on more specific details of how to truly implement your telehealth program. While we have displayed these steps chronologically, we know that the real world is not always straightforward. Use this process as a guide while understanding that:

- ❑ The order of steps may shift based on your practice or organization
- ❑ Some steps may overlap or may be executed simultaneously
- ❑ Some steps may take more or less time than others
- ❑ You may need to reiterate or circle back to an earlier step when expanding your program or if challenges arise

We recommend reading through the Playbook in its entirety before embarking on the path to implementation so you know what lies ahead. Then, once you've commenced the process in practice, refer to each step in **Part 2** and **Part 3** for best practices, checklists for success, and practice spotlight stories to guide you along your way.

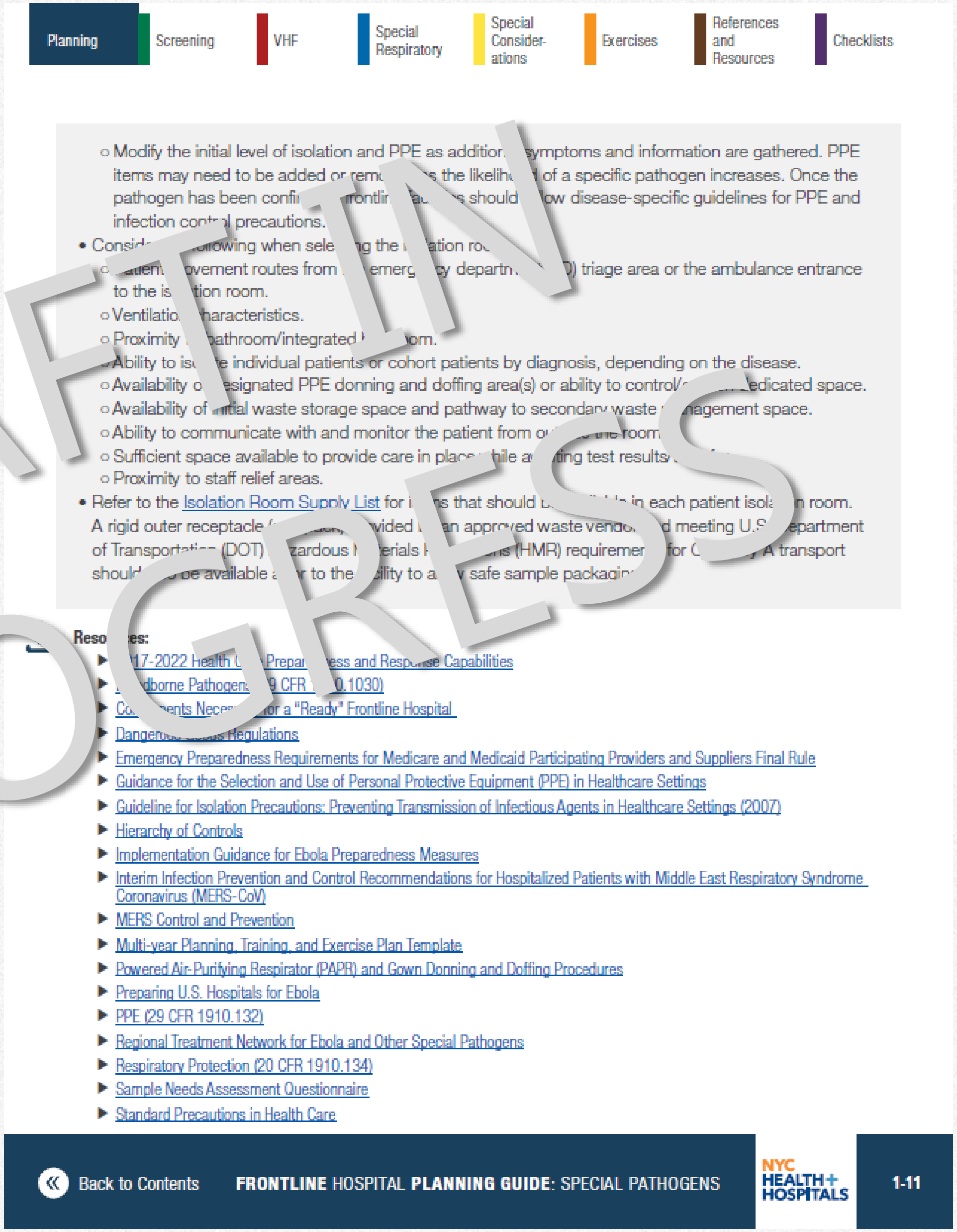

- o Modify the initial level of isolation and PPE as addition[illegible] symptoms and information are gathered. PPE items may need to be added or rem[illegible]s the likelih[illegible] of a specific pathogen increases. Once the pathogen has been confi[illegible] frontlir[illegible] fa[illegible]s should [illegible]ow disease-specific guidelines for PPE and infection cont[illegible]l precautions.
- Consid[illegible]ollowing when sele[illegible]g the i[illegible]ation ro[illegible]
  - o Patien[illegible]ovement routes from [illegible] emerg[illegible]cy departn[illegible]) triage area or the ambulance entrance to the is[illegible]tion room.
  - o Ventilatio[illegible]haracteristics.
  - o Proximity [illegible]bathroom/integrated [illegible]om.
  - o Ability to is[illegible]e individual patients or cohort patients by diagnosis, depending on the disease.
  - o Availability o[illegible]esignated PPE donning and doffing area(s) or ability to control/[illegible]edicated space.
  - o Availability of [illegible]itial waste storage space and pathway to secondary waste [illegible]agement space.
  - o Ability to communicate with and monitor the patient from o[illegible] the room.
  - o Sufficient space available to provide care in place [illegible]hile a[illegible]ting test results [illegible]
  - o Proximity to staff relief areas.
- Refer to the Isolation Room Supply List for i[illegible]s that should [illegible] in each patient isol[illegible]n room. A rigid outer receptacle [illegible]vided [illegible]an approved waste vendo[illegible]d meeting U.S[illegible]epartment of Transportation (DOT) [illegible]azardous [illegible]erials [illegible]s (HMR) requiremen[illegible] for [illegible] A transport should [illegible] be available [illegible]r to the [illegible]ility to a[illegible] safe sample packagin[illegible]

Reso[illegible]es:

- [illegible]17-2022 Health [illegible] Prepar[illegible]ess and Resp[illegible]se Capabilities
- [illegible]dborne Pathogens [illegible]9 CFR [illegible]0.1030)
- Co[illegible]nents Neces[illegible]or a "Ready" Frontline Hospital
- Dangerous [illegible] Regulations
- Emergency Preparedness Requirements for Medicare and Medicaid Participating Providers and Suppliers Final Rule
- Guidance for the Selection and Use of Personal Protective Equipment (PPE) in Healthcare Settings
- Guideline for Isolation Precautions: Preventing Transmission of Infectious Agents in Healthcare Settings (2007)
- Hierarchy of Controls
- Implementation Guidance for Ebola Preparedness Measures
- Interim Infection Prevention and Control Recommendations for Hospitalized Patients with Middle East Respiratory Syndrome Coronavirus (MERS-CoV)
- MERS Control and Prevention
- Multi-year Planning, Training, and Exercise Plan Template
- Powered Air-Purifying Respirator (PAPR) and Gown Donning and Doffing Procedures
- Preparing U.S. Hospitals for Ebola
- PPE (29 CFR 1910.132)
- Regional Treatment Network for Ebola and Other Special Pathogens
- Respiratory Protection (20 CFR 1910.134)
- Sample Needs Assessment Questionnaire
- Standard Precautions in Health Care

DRAFT IN PROGRESS

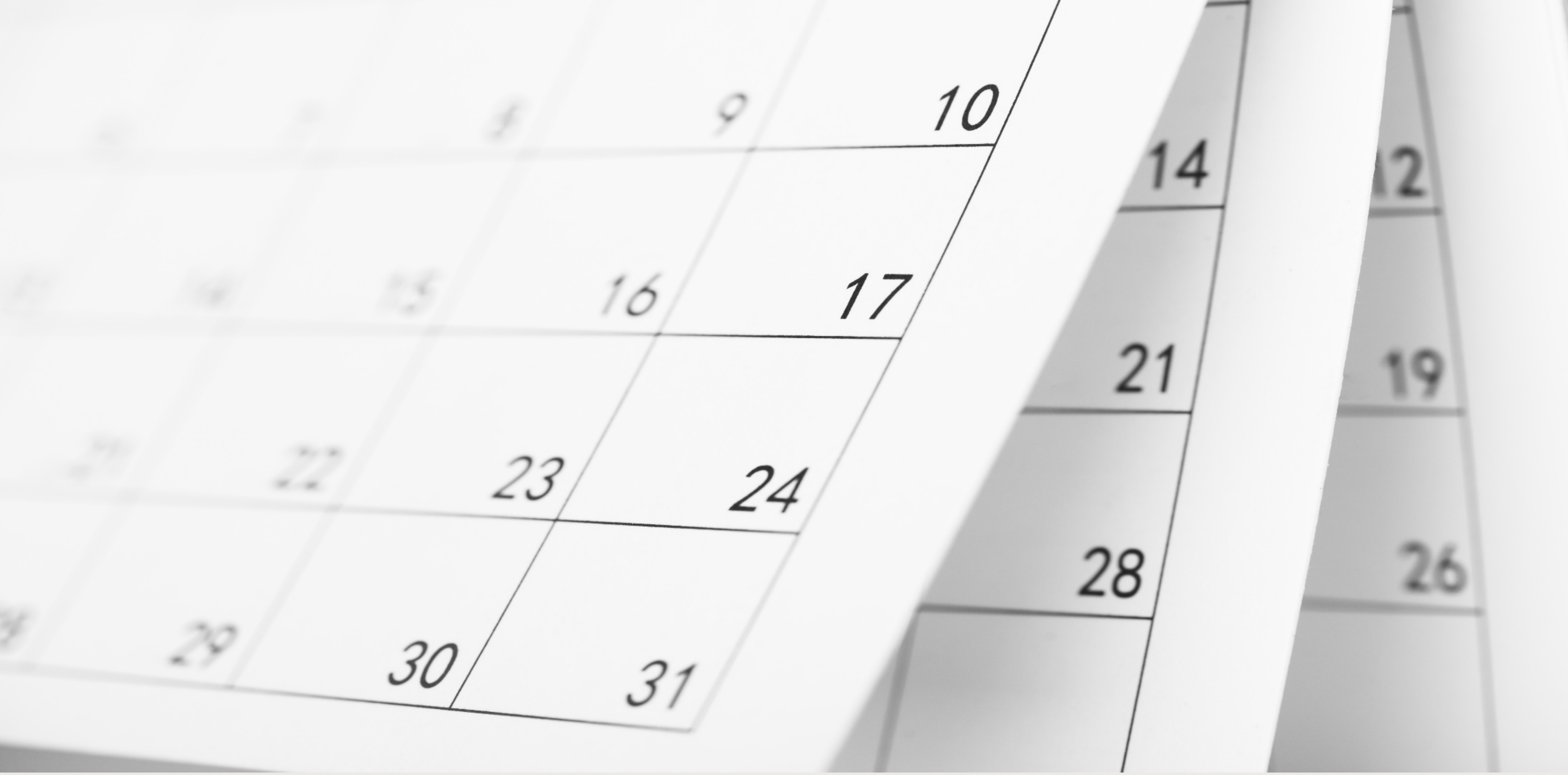

SCHEDULE

PROJECT TIMELINE

Project Timeline

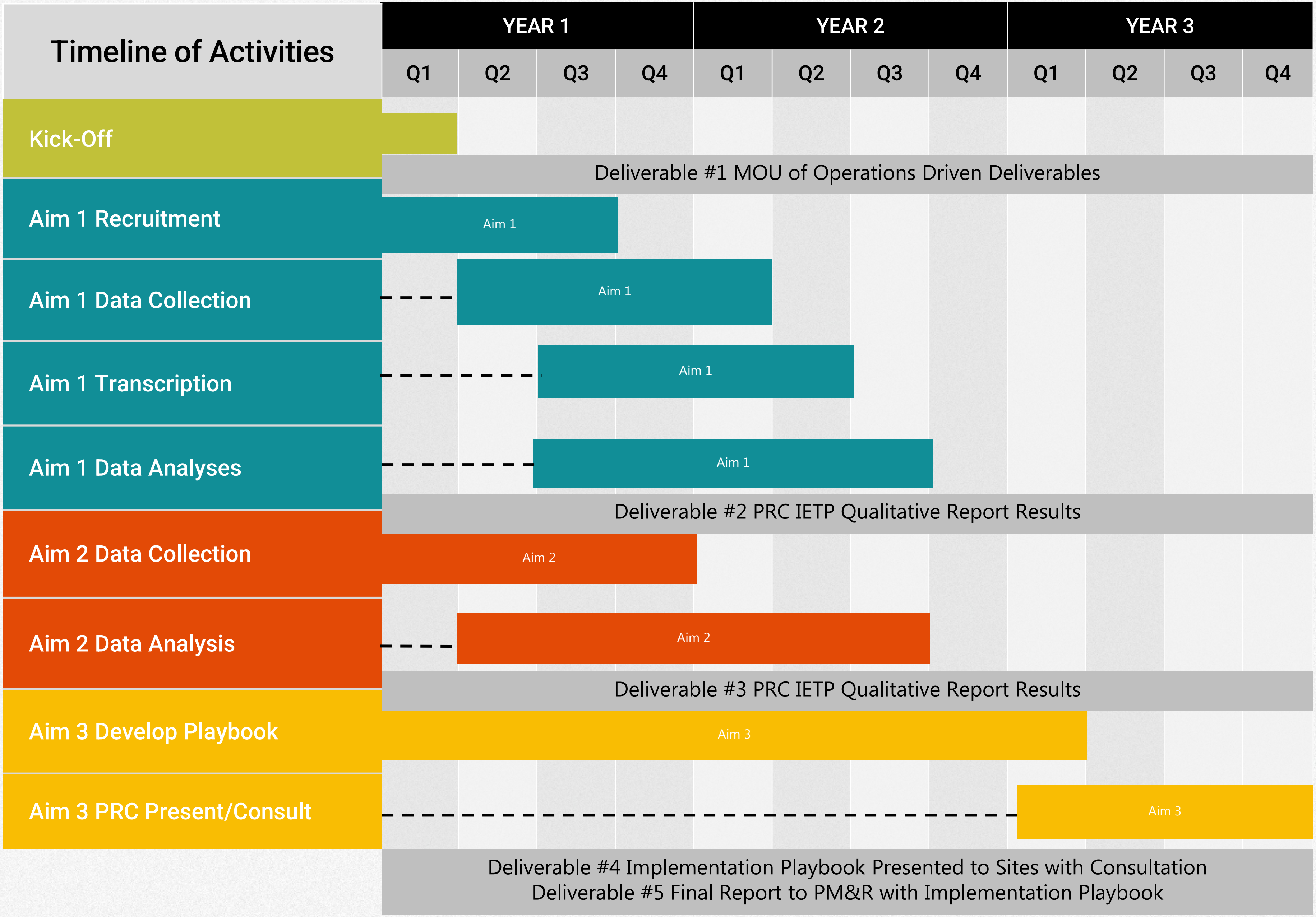

Supplement: Multimedia Appendix 4 [file resprot_v13i1e59830_app4.pdf]
