# Supplementary material for: Developing Evidence to Support Policy: Protocol for the StrAtegic PoLicy EvIdence-Based Evaluation CeNTer (SALIENT)
Source: JMIR Res Protoc. 2024 Sep 19;13:e59830. doi: 10.2196/59830 (PMC11450355; doi:10.2196/59830)
Supplement: Multimedia Appendix 5 [file resprot_v13i1e59830_app5.pdf]

Post-project team evaluation template

Evaluation Standards

**Utility**	To what degree do you feel our team has achieved the following? (Likert scale 1-5)<br>Our team used our expertise to establish and maintain credibility in <insert project>?<br>Our team identified the individuals and group stakeholders invested in <insert project>?<br>Our team effectively designed the evaluation to meet the needs of stakeholders?<br>Our team understood and clarified unique cultural values for this project?<br>Our team delivered results that served the identified and emergent needs of stakeholders?<br>Our team effectively constructed and executed <project activities/processes/products> (revise based on project) that delivered meaningful products to our stakeholders?<br>Open-ended (*OE*): *Are there specific instances or examples of ways our team excelled or need to improve in this area?*
**Feasibility**	To what degree do you feel our team has achieved the following? (Likert scale 1-5)<br>Our team used effective project management strategies to <insert goal> of <project>?<br>Our team adapted project management strategies as necessary to accomplish our goal?<br>Our team used resources effectively and efficiently?<br>*OE: What barriers occurred and what could we do in the future to mitigate these?*<br>*OE: Were there specific project management strategies that either facilitated or deterred our ability to accomplish our goal?*
**Proprietary**	To what degree do you feel our team has achieved the following? (Likert scale 1-5)<br>Our evaluation agreement/project scope of work accounted for the needs, expectation, and cultural contexts of our stakeholders and their communities?<br>Our team was responsive to our stakeholders through our research and dissemination of findings?<br>Our <project> was designed and conducted to protect human and legal rights and maintain the dignity of participants/stakeholders/Veterans?<br>Our <project> was conducted in a way that was transparent and understandable for our stakeholders?<br>Our <project> fairly and adequately addressed stakeholder needs and purposes?<br>*OE: What did our team do to successfully conduct the project in alignment with stakeholder needs? What could we have improved?*
**Accuracy**	To what degree do you feel our team has achieved the following? (Likert scale 1-5)<br>Our team was able to situate and justify <project findings/conclusions/decisions> according to our stakeholder needs and culture?<br>Our <project findings/conclusions/decisions> met <project goals>?<br>Our <project findings/conclusions/decisions> were dependable and supported valid interpretations?<br>Our team documented the process of <project findings/conclusions/decisions> with appropriate detail and scope for this project?<br>Our team used systematic and appropriate data collection, review, verification and storage methods, ensuring data security was met (if appropriate describe MOUs or other Data sharing agreements)?

	Our team employed methods that were appropriate for this project?<br>Our team clearly documented all analyses, findings, interpretations, conclusions, and judgments from our evaluation methods?<br>Our team disseminated research results clearly without biases, distortions, and errors?<br>*OE: What processes facilitated or deterred our efforts for accuracy?*
**Accountability**	To what degree do you feel our team has achieved the following? (Likert scale 1-5)<br>Our team transparently documented evaluation steps internally (with the team)?<br>Our team transparently documented evaluation steps externally (with our stakeholders)?<br>Our team assessed progress and adapted as necessary?<br>*OE: What specific recommendations do you have to improve our internal and external accountability?*
**Collaboration and team commitment**	Please rate the degree to which you agree with the following statements. (Likert scale 1-5)<br>People on our team have collaborated well on this project.<br>People on our team have a history of collaborating well together.<br>The right teams and individuals have been brought together for this collaboration/project.<br>Our team has a history of bringing the right teams and individuals together to accomplish great work.<br>People on our team are willing to compromise on important aspects of our work to achieve our goals.<br>People on our team invest the right amount of time and energy to achieve our goals.<br>People on our team wanted this project to succeed.<br>When making decisions, people on our team are able to provide opinions and consider possibilities for action.<br>People on our team have a clear sense of roles and responsibilities.<br>People on our team are open to different approaches in how we do our work.<br>People on our team communicate openly with each other.<br>I feel I am a valued member of our team.

Notes/Citations:

Evaluation Standards are derived and adapted from MacDonald, G. (2013) *Framework for Program Evaluation in Public Health: A Checklist of Steps and Standards,* Prepared for the Center for Disease Control and Prevention.

Collaboration and team commitment section adapted from *The Wilder Collaboration Factors Inventory.*
