# Supplementary material for: Developing Evidence to Support Policy: Protocol for the StrAtegic PoLicy EvIdence-Based Evaluation CeNTer (SALIENT)
Source: JMIR Res Protoc. 2024 Sep 19;13:e59830. doi: 10.2196/59830 (PMC11450355; doi:10.2196/59830)
Supplement: Multimedia Appendix 6 [file resprot_v13i1e59830_app6.pdf]

SUMMARY STATEMENT

PROGRAM CONTACT: (Privileged Communication) *Release Date:* 03/22/2022

Revised Date:

Application Number: 1 I50 HX003619-01

Principal Investigators (Listed Alphabetically):

PUGH, MARY JO (Contact)
WHITE, PAUL JONATHAN

Applicant Organization: VA SALT LAKE CITY HEALTHCARE SYSTEM

Review Group: HQ8
QUERI

Meeting Date: 03/03/2022 *RFA/PA:* HX22-026
Council: MAY 2022
Requested Start: 04/01/2022

Project Title: SAlt Lake EvIdence-Based EvaluatioN CenTer for Policy (SALIENT Policy)

SRG Action: Impact Score:243

Human Subjects: 10-No human subjects involved
Animal Subjects: 10-No live vertebrate animals involved for competing appl.

Project Year	Direct Costs Requested
1	817,500
2	817,500
3	817,500
4	817,500
5	817,500
TOTAL	4,087,500

ADMINISTRATIVE BUDGET NOTE: The budget shown is the requested budget and has not been adjusted to reflect any recommendations made by reviewers. If an award is planned, the costs will be calculated by VA Office of Research and Development (ORD) staff based on the recommendations outlined in the BUDGET COMMENT section and any relevant ORD service-specific limitations.

KEY SUMMARY POINTS:

1. The proposal describes innovative approaches to evidence-based policymaking, including the use of Lean methodology.
2. There is a nice linkage to the Evidence Act, providing support that the proposed center would meet the needs of this legislation.
3. There is a lack of detail on the cross-core collaborations, implementation methods and outcomes, and how the evaluation results and impacts would be translated into policy.
4. More detail is needed on how SALIENT's specific approaches to policy evaluation would be integrated into the training programs.

DESCRIPTION (provided by applicant):

The overall goal of VA Salt Lake City Health Care System's SAlt Lake EvIdence-Based EvaluatioN CenTer for Policy (SALIENT) is to conduct evaluations that support adoption of programs and policies which are aligned with VA priorities. Our impact will be to accelerate generation, dissemination, and implementation of evidence-based policy recommendations, pursuant to the Evidence Act. To accomplish this goal, we will achieve three objectives for our center: Objective 1. Develop and conduct comprehensive evaluations utilizing a wide range of approaches (e.g., quantitative, qualitative, mixed methods, policy analysis, economic, clinical informatics, and implementation science) to rapidly address VA's strategic missions and priority evaluation areas. Objective 2. Develop and deploy knowledge translation resources to rapidly communicate findings and assist in implementing evidence-based practices. Objective 3. Train the next generation of evaluation and implementation scientists. Methods: We will use Lean Sprint methodology (iterative, incremental, rule-governed approach to clearly defined, time-boxed work) to develop our evaluation plans collaboratively with operational partners, key stakeholders including Veterans, policy experts, and clinicians. Our strong cores and collaborative relationships among those cores will facilitate rapid development of rigorous evaluation plans and high impact evaluations that use state of the science methodology and state of the art dissemination approaches. The Operations Core will use Lean Six Sigma processes to develop timelines with evaluation teams, facilitate work, monitor progress, and guide quality improvement within SALIENT throughout and after evaluations. The Methods Core will work with evaluation teams to identify the most appropriate qualitative, quantitative, and mixed methods approaches to address each evaluation, ensure that the analyses are conducted appropriately, troubleshoot issues with data acquisition/analysis, and ensure appropriate description of results. The Knowledge Translation Core will target key stakeholders and decision makers and parse these groups using a needs-based market segmentation approach to ensure that those needs are incorporated in the evaluation. The KT Core team will create communications briefs, playbooks, and other materials targeted at these market segments to facilitate implementation of evidence-based practices and maximize impact of evaluation results. The SALIENT team proposes two evaluations as examples of the acumen and ability of SALIENT to engage in high priority evaluation efforts (homelessness among women Veterans and behavioral health community care utilization). In addition, we demonstrate how our team has engaged in our Core missions and operations to rapidly evaluate a high priority area of women Veteran un- and underemployment. The SALIENT team has a long history of collaborations with VA partners including those associated with the two proposed evaluations, which address both VA FY23 performance metric priorities and requirements of the Hannon Act. SALIENT has demonstrated the ability to rapidly pivot and assemble research teams to evaluate strategic missions and priority evaluation areas identified by VA Central Office. With an array of resources, faculty experts, and operational partners to support center efforts, SALIENT has an established and evolving infrastructure to rapidly develop and implement high-impact evaluations, pivot to new evaluation priorities as needed, effectively translate knowledge for key stakeholders and policymakers, and train the next generation of evaluation and implementation scientists.

CRITIQUE 1

1. Significance and Impact

These two proposed topics are highly important problems in the VA. The Center plans to inform work policies and programs affecting Veteran care by developing the evaluation in collaboration with its operational partners, including engaging with Veterans, and will target the dissemination of its results to specific stakeholders relevant to the topics and disseminate its playbooks for implementation. The concepts, approaches, and mixed methodologies (Lean Sprint groups, Lean Six Sigma, and Periodic Reflections) outlined in this application are not novel per se; however the applications of the proposed collective methods to evaluate policies are novel and may inform the VA's approach to evidenced-based policies. The proposed development of an Evaluation Scorecard with key goals for future implementation is an innovative approach.

<u>Strengths:</u>
The proposed evaluation topics (Women Veterans homelessness and Behavioral Health Community Care Utilization) are important clinical priorities to address for VA. SALIENT plans to use Lean methodology (Lean Spring groups, Lean Six Sigma) to rapidly engage stakeholders and plan the evaluations. The application of these methodologies to policy evaluation are novel. They plan to use the QUERI ACTION framework to guide their evaluations and embed key stakeholders, clinicians, and policy experts into the SALIENT team along with planned communications and collaboration among the cores.

The SALIENT Scorecard is innovative.

<u>Weaknesses:</u>
The proposed impacts on how implementation/evaluation is done in the VA are not clear.

2. Approach and Feasibility

<u>Strengths:</u>
The overall quality of the proposed cores for each evaluation is excellent both internally and externally to SALIENT. MPIs, Dr. Pugh and Dr. White, have years of experience managing teams successfully. Together they will lead the Operational Core. The Methods Core includes both quantitative and qualitative methods. Dr. Vanneman has expertise in health policy, economics, and organizational behavior. Dr. Nelson will lead discussions on economic evaluations for the Center. Dr. Zickmund holds extensive experience running a qualitative core locally in Salt Lake City and across the VA for qualitative transcription services. Drs. Fagerlin and Kean will lead the Knowledge Translational Core. Dr. Fagerlin has expertise in risk communication and development of plain language patient and clinical educational tools. Dr. Kean holds applied implementation expertise.

The OPS Core proposes the application of the Lean Six Management model as its approach to policy evaluation. The systematic application of this management model is innovative. The SALIENT leadership will convene the LEAN Sprint team to collaboratively define the evaluation plan. The Ops Core will facilitate the Methods and KT cores including well-defined milestones and

timelines and monitor performance of evaluations using those milestones. An applied example of the Lean Sprint group is provided in proposal.

The Salt Lake City VAMC has VA advanced fellowships in health services research, informatics, geriatrics, mental illness research and treatment, health professions education evaluation and research, and the CARRIAGE QUERI mentoring core with additional fellowships at the University of Utah for which four of the SALIENT co-investigators lead (Drs. Knight, Rubin, Fagerlin, and Gordon) and will permit the SALIENT trainees (students, fellows, junior faculty) to join the existing training programs. The vast training opportunities for SALIENT trainees at the SLCVAMC is a strength.

SALIENT Knowledge Translation will have access to the Sorenson Impact Center at the UU. This Center assists evaluations with dissemination through data visualizations, films, social media strategies. These are innovative dissemination strategies and are a strength of the application.

The proposed outcomes include quality of care, health care utilization, clinical outcomes, mortality, costs, community care utilization, comorbidities, and employment data.

Based on the proposed evaluation methods, the SALIENT evaluations appear feasible for completion in the proposed timeline.

Weaknesses:

While the proposal discusses the health, quality, and economic outcomes that will be targeted and how they will inform policies and programs for Veteran populations, it’s unclear how SALIENT will advance how implementation, dissemination and knowledge translation is done in VA.

Additionally, it’s unclear how the planned evaluations with health and economic outcomes will inform implementation playbooks. This is a major weakness. No implementation strategies, processes, or outcomes are listed in the proposed evaluations. The budget justification for co-investigator, P Jon White, MD, states he will collaborate with the KT core to disseminate implementation strategies – this is the only mention of implementation strategies. How will SALIENT articulate implementation strategies and an implementation playbook from its evaluations and associated outcomes?

A weakness is the lack of training plans on the specific approach for SALIENT policy evaluation – Lean Sprint, Lean Six Sigma, Periodic reflections with feedback loops, etc.

Page 42: “The Lean tools will be used to create Value Stream Maps of schematics used to identify activities that have value to stakeholders as well as waste, delays, and inefficiencies” – What activities does this refer to? What types of inefficiencies?

Salient investigators have experience evaluating health disparities and health equity and VA Office of Health Equity is a partner; however it is not specified how equity will be evaluated. This is a weakness. In addition, SALIENT will evaluate stakeholder input, but there is no discussion of end user experience.

3. Integration of Core Activities

Strengths:

The SALIENT proposal clearly demonstrates strong evidence of administrative integration, and the SALIENT team of investigators are well-woven into their existing work, organizations, and partnerships. There is evidence of coordination, interrelationships, and synergies among the individual cores. Although SALIENT will use Lean Six Sigma principles to guide its policy evaluation processes, the scientific integration across SALIENT is less defined in the proposal.

Weaknesses:
No comment.

4. Center Leadership and Environment

Strengths:
The Multiple PD/PI MPI leadership plan is adequate, and the MPIs have complementary and integrated experience. Dr. Pugh is a VA Career Research Scientist with a long history of VA evaluation and QI/QA work and a Professor of Medicine and Epidemiology at the University of Utah. She has over 20 years of VA/DoD QI and evaluation experience. Dr. White's career has been dedicated to advancing the use of evidence to improve health and health care. He is currently the Associate Chief of Staff for Research and Development at VA SLCHCS. Formerly, Dr. White was the Deputy National Coordinator for Health Information Technology in the US Department of Health and Human Services from 2015-2019 and previously he directed the health IT research portfolio at AHRQ. The qualifications, experience, and commitment of the center, core leads, and key personnel are adequate and appropriate to complete the proposed evaluations. The Salt Lake City VAMC, University of Utah, and stakeholder environment are strong providing a high probability of SALIENT success.

Weaknesses:
No comment.

5. Quality Improvement/Protection of Human Subjects

The proposed QUERI SALIENT evaluation center has a primary purpose of evaluation of existing programs and to inform operational partners as directed by VA operations or entities responsible for overseeing VA, such as Congress.

There are no concerns with human subjects.

6. Budget and Period of Support

No budget concerns.

7. Key Strengths

1. The proposed SALIENT evaluation topics (Women Veterans homelessness and Behavioral Health Community Care Utilization) are important clinical priorities to address for VA. SALIENT plans to use Lean methodology (Lean Spring groups, Lean Six Sigma) to rapidly engage stakeholders and plan the evaluations. The applications of these methodologies along with the development of an Evaluation Scorecard to policy evaluation are novel.

2. The overall quality of the proposed cores for each evaluation is excellent. MPIs, Dr. Pugh and Dr. White, have years of complementary experience managing teams successfully and with conducting QI evaluations and translating evidence into practice. The qualifications, experience, and commitment of the center, core leads, and key personnel are adequate and appropriate to complete the proposed evaluations. The Salt Lake City VAMC, University of Utah, and stakeholder environment are strong providing a high probability of SALIENT success.

3. The proposed policy evaluations are original, feasible, and rigorously planned. The OPS Core proposes the application of the Lean Six Management model as its approach to policy evaluation. The systematic application of this management model is innovative. An applied example of the Lean Sprint group is provided in the proposal. The cost evaluations will be planned from the perspective of the VA.

8. Key Weaknesses

1. While the proposal discusses the health, quality, and economic outcomes that will be targeted and how they will inform policies and programs for Veteran populations, it's unclear how SALIENT will advance how implementation, dissemination and knowledge translation is done in VA.

   Additionally, it's unclear how the evaluations with health and economic outcomes will inform implementation playbooks. This is a major weakness of SALIENT's proposal. No implementation strategies, processes or outcomes are listed in the proposed evaluations. How will SALIENT articulate implementation strategies and an implementation playbook from its evaluations and associated outcomes?

2. Some of the planned impacts are less defined. SALIENT investigators have experience evaluating health disparities and health equity and VA Office of Health Equity is a partner; however, it is not specified how equity will be evaluated. In addition, SALIENT will evaluate stakeholder input, but there is no discussion or proposed methods of end user experiences.

3. Although there are many existing fellowships and training opportunities within SLCVAMC and UU with some led by the co-investigators, the weakness of the proposed training plan is the lack of training plans on the specific approach for SALIENT policy evaluation – Lean Sprint, Lean Six Sigma, Periodic reflections with feedback loops, and how to inform VA programs, budgets and policies.

CRITIQUE 2

1. Significance and Impact

<u>Strengths:</u>
The application addresses important areas, to support evidence-based policymaking, specifically related to women Veterans and Veterans who are homeless.

The work has the potential to inform care.

<u>Weaknesses:</u>

It is unclear how the application fits into the overall existing program evaluation structure that already is present within VA Central Office.

Partnerships with existing policy and program evaluation centers need to be better described. How leaders will be provided with the evidence to fully assess the impact of policies should also be clarified.

The overall significance and impact is not clearly described or articulated. Additional operational details would be helpful.

2. Approach and Feasibility

Strengths:
Overall quality and adequacy of services appear adequate.

The design is well-described and adequate.

The use of Lean Six Sigma and Lean Sprint methodology is a strength.

Weaknesses:
The application describes a wide range of approaches for conducting analyses. Although this can be a strength when flexibility of approach is a priority, for the current application, it lacks specificity and does not include an appropriate level of detail to adequately describe what is being proposed.

The application does not provide sufficient evidence to fully determine if the proposed evaluations can be fully completed for the projects noted. More specifically, there is a lack of detail and clarity about how the proposed plan will be operationalized.

3. Integration of Core Activities

Strengths:
There is appropriate synergy within the environment described, and the integration of the cores and core activities appear adequate and sufficient to complete the proposed projects.

The depth and breadth of experiences of the team is a strength.

Weaknesses:
Additional descriptions of how the cores will be integrated and function as a cohesive team would be beneficial. It is unclear how they will function together, which may limit impact.

It is not entirely clear how it will be advantageous to conduct the proposed work as a center rather than as individual projects. It would be helpful to more clearly define how the projects will align together and create synergy across the cores.

4. Center Leadership and Environment

Strengths:
The Center leadership and environment are a strength of the application.

Weaknesses:

None noted.

5. Quality Improvement/Protection of Human Subjects

No concerns and appears to meet standards for non-research quality improvement.

6. Budget and Period of Support

Budge appears appropriate given requested period of support and proposed project.

7. Key Strengths

1. Potential impact on women Veterans is a strength as well as for Veterans who are homeless.

2. Training the next generation of scientists is a strength.

3. The environment is a strength and is likely conducive to supporting this application.

8. Key Weaknesses

1. Overall, the application lacks sufficient detail on specific methods or potential impact.

2. There is a lack of detail and clarity about how the proposed plan will be operationalized.

3. It would be helpful to more clearly define how the projects will align together and create synergy across the cores.

CRITIQUE 3

1. Significance and Impact

Strengths:
Addressing critical problems: The proposed project, SALIENT will focus on two issues: homelessness among women Veterans and expanded access to behavioral health during the COVID-19 pandemic. The background and need for research in this area is well-defined – especially as applied to women and other Veteran populations that suffer health inequities.

Weaknesses:
Information is not provided regarding how the projects map on to specific VA priorities. How the projects will improve how implementation/evaluation/QI is done in the VA is not clearly stated.

2. Approach and Feasibility

Strengths:
The quality of the cores is strong – specifically the methods core, which brings vast experience in using a variety of methods needed to complete the evaluations.

Methodologies are outlined and appropriate for the type of evaluations proposed.

Knowledge translation – dissemination products are outlined for both projects and seem adequate – will be determined as findings emerge.

Problems addressed – challenges are outlined for both evaluations and strategies to overcome challenges are described.

Milestones – milestones for both evaluations and entire Core are outlined and seem realistic.

Potential for completion – utilizing a state of the art management system and vast experience should provide for completion of the milestones as outlined.

Weaknesses:
No conceptual model or implementation frameworks are outlined in guiding the evaluation projects/methods proposed.

3. Integration of Core Activities

Strengths:
Proposal outlines how the three cores will be managed (specifically using Lean Sprint and Six Sigma) and work is integrated. Proposal also outlines how cores will collaboratively mentor diverse students, post-doctoral fellows, and junior faculty for the Advancing Diversity in Implementation Leadership program.

Scientific and administrative integration is well-defined, and use of the management system to inform the need for process improvements is a significant strength.

Weaknesses:
None.

4. Center Leadership and Environment

Strengths:
Probability of success is strong – there is a strong management plan which will be executed by an interdisciplinary leadership and research team with extensive subject matter and technical expertise and research execution and management experience.

SALIENT also has a strong infrastructure of support from a variety of partners to ensure successful completion of projects and core activities and broad use of findings.

Weaknesses:
Lacking dissemination and implementation science expertise.

5. Quality Improvement/Protection of Human Subjects

Does not qualify for human subjects research given the nature of evaluation of VA programs.

6. Budget and Period of Support

No noted issues or suggestions for revision.

7. Key Strengths

1. SALIENT will be executed by an experienced, multidisciplinary team with deep evaluation and methodological expertise. The proposed evaluations address quality of Veteran care, specifically as it relates to homelessness among women Veterans and access to behavioral health care during the COVID-19 pandemic.

2. Use of state of the art management platform for overall management of evaluation core and strong integration across cores defined.

3. Extensive methodology expertise and experience to conduct novel approaches to evaluate programs and to inform policy.

4. Extensive collaboration across internal and external partners.

8. Key Weaknesses

1. Lacking use of implementation science for informing methods/design of evaluations.

2. Unclear how proposal advances how implementation/evaluation is done in VA.

3. Lacking implementation science expertise.
