# Supplementary material for: Developing Evidence to Support Policy: Protocol for the StrAtegic PoLicy EvIdence-Based Evaluation CeNTer (SALIENT)
Source: JMIR Res Protoc. 2024 Sep 19;13:e59830. doi: 10.2196/59830 (PMC11450355; doi:10.2196/59830)
Supplement: Multimedia Appendix 7 [file resprot_v13i1e59830_app7.pdf]

Introduction to Application

Thank you for the opportunity to improve our application. Based on the initial review, we identified the following proposal strengths: **1)** innovative approaches to evidence-based policymaking, including the use of Lean methodology; **2)** innovative use of the SALIENT scorecard; and **3)** the strong linkage between the SALIENT center and the Evidence Act. We respond to Summary Key Points and other reviewer comments below and illustrate the changes the application with italics. Where tables are new, we italicize the title only. For new figures we italicize the Figure number in the text.

1. There is a lack of detail on the a) cross-core collaborations, b) implementation methods and outcomes, and c) how the evaluation results and impacts would be translated into policy (Key Point 3).

a. Detail on cross-core collaboration (also R2, Weakness 3). Detail on cross-core collaboration in our first submission was scattered throughout the proposal, diffusing impactful illustration. To focus and simplify, we concentrate discussion of cross core collaboration in overarching processes (**B.2**), as part of the Lean Six processes in **B.3.1**, project descriptions (**B.4**), and developing best practices in policy evaluation and implementation (**B.6**). *A key feature of our Lean Six Sprint/Sigma approach is significant cross core collaboration.* We also reconfigured the cores and leads to promote engagement and collaboration across cores, projects, and SALIENT sites, ultimately integrating SALIENT into a cohesive effort in policy evaluation and implementation. This integrated approach is also illustrated in the management plan **(C.3)**.

Reviewer 1 noted that the scientific integration across SALIENT is less defined in the proposal. We agree with the reviewer that scientific integration across the projects and cores was less defined. To address this issue, we have established a strong guiding conceptual framework to inform and integrate within and across projects. We also describe how similarities across studies regarding complex patient management and methods and our approach to using Lean Six processes to integrate findings from each study to allow rapid comparison of themes across both evaluation processes and results **(B.4).** As described in **B.4** and **B.6** we will synthesize relevant constructs, implementation strategies, communication strategies, mechanisms, and outcomes to inform best practices and optimize impact over time. Our Lean Six approach also integrates teams using quarterly SALIENT team meetings including a half day annual meeting to share scientific approaches and stimulate cross-team discussion (see **C.3** Center Management Plan).

Reviewer 2 asked that we describe how it will be advantageous to conduct the proposed work as a center rather than individual projects and describe how projects will align together and create synergy across the cores. We will create synergy in the SALIENT Center in the following ways. First, cores and approaches to Lean Six Sprint/Sigma process and the conceptual framework are integrated throughout evaluation projects to maximize cross-core collaboration and synthesis over time. Second, we will use of Six Sigma processes allows more rapid conduct of evaluations to inform our operational partners in real time, allowing responsive adaptation as needed, allowing evaluations to benefit from the emerging learning evaluation system, promoting learning across cores and projects to improve outcomes and maximize evaluation impact (**B.4**; **B.6**; **C.3**). Third, evaluations collectively focus on care for Veterans with complex comorbidity (opioid use disorder, chronic multi-symptom illness) and use quantitative and qualitative approaches. In quarterly and annual SALIENT meetings, we will share findings across studies, allowing for cross fertilization of ideas, methods, and findings across studies over time. Fourth, each project will inform development of best practices in processes, protocols for policy evaluation, and implementation to create a model approach, i.e., score cards, playbooks, mapped implementation science, communication assessment/ procedures, and communicating evidence to stakeholders to maximize impact. Fifth, we will create a SALIENT Evaluation Policy Catalog to document relevant constructs, implementation strategies, communication strategies, mechanisms, and outcomes for each evaluation. Integration and analysis of these findings will be used to develop best practices in policy evaluation and implementation and optimize impact over time.

b. Detail on implementation methods and outcomes. We have integrated a conceptual framework (**Figure 2**) that is grounded in CFIR, RE-AIM and evidence-based implementation strategies that informs development of an implementation research logic model (IRLM) to guide our evaluations and outcomes (e.g., implementation, policy, service, and health outcomes). Our use of Lean Six Sigma will facilitate a systems re-design approach and a continual feedback loop throughout the evaluation process. The proposed projects illustrate the integrated use of the conceptual framework (**4.1.f**; **4.2.f**).

c. Detail on how the evaluation results and impacts would be translated into policy (also R1 Weakness 2). Each evaluation will: first, start with a Lean Six Sprint/Sigma approach to identify targeted audiences and stakeholders and establish a systems redesign approach to center evaluation process; second, include key stakeholder and gatekeepers as collaborators; third develop strong feedback loops to be responsive to stakeholders/leaders (for sensitive groups/topics we will send reports/manuscripts in advance for feedback on

policy implications); and fourth, result in a playbook with recommendations, communication strategies, products, and score card to inform a strategic process to implement policy evaluation findings. In addition to project specific dissemination and knowledge translation efforts, the SALIENT Center will: (1) develop opinion/editorials as we have done in the Elizabeth Dole Center of Excellence for Veteran and Caregiver Research; (2) develop key data presentations to administrative policy makers; (3) conduct quarterly webinars; (4) include key stakeholders and opinion leaders as co-authors to improve operational partner engagement and uptake in the field; (5) establish an ongoing newsletter forum (e.g. HSR&D); and (6) maintain a resource and training Sharepoint site. We will incorporate feedback directly with end users throughout the process as part of Lean Six processes (**B.3.1; B.6**) as we have done with our women Veterans' unemployment study (**WVU**), which has been described as "phenomenal" by our partner, the Center for Women Veterans**.**).

2. More detail is needed on how SALIENT's specific approaches to policy evaluation would be integrated into the training programs (**Key Point 4;** R1 Key Weakness 2)**.** Our Lean Six Sprint/Sigma approach is innovative and will help our evaluation team communicate implementation issues with our operational partners and stakeholders who are more commonly trained in LEAN than implementation science terminology. This is a unique opportunity for trainees. All ADIL fellows, post-doctoral fellows, and graduate students will participate in existing VA Lean Six Sigma training and get exposure to policy evaluation projects through presentations and products; fellows will participate in a quarterly webinar on key practices such as Lean SPRINT methods, periodic reflections with feedback loops, and how to use findings from inform VA programs, budgets, and policies. We will invite all fellows in VA SLC programs and fellows from our spoke sites to participate in these seminars which will include reading, discussion, and practical application.

3. How will SALIENT advance how implementation, dissemination, and knowledge translation is done in VA (Reviewer 1 Key Weakness 1; Reviewer 2 Key Weaknesses 2,3). We will advance the use of implementation science (IS) and knowledge translation (KT) to create best practices in policy evaluation and implementation using five steps. First, we will employ the Lean Six Sprint/Sigma process to launch and conduct each evaluation (**B.3.2; B.3.3**). Second, we are using a cohesive approach that relies on CFIR, RE-AIM and the IRLM process to inform project conceptualization, data collection, analysis, implementation strategy development, reporting, synthesis, and dissemination over time (**B.1**). Third, we will develop a strategic systematic approach to developing playbooks to maximize impact of policy evaluations conducted by SALIENT (**B.5**); Fourth, we will develop investigators with expertise in evaluation & KT/IS (**B.7**). Fifth, we will develop the SALIENT Policy Evaluation Catalog of projects over time to document project factors over time (i.e., policy type, stakeholders, constructs, implementation strategies, communication strategies, mechanisms, outcomes, dissemination strategies) to effectively integrate and develop best practices in policy evaluation **(B.6)**. Moreover, we propose to collaborate with other centers to collect these data on their evaluations to inform large scale synthesis of policy evaluation and implementation best practices over time **(B.6)**. Data from our inventory can be used by SALIENT, other centers, and the Coordinating Center for cross-project observations and learning, which is a broader opportunity for overall QUERI development.

4. Reviewer 3 concerns about implementation science expertise. We added Jolie Haun PhD as an MPI. Dr. Haun brings extensive expertise in implementation science, playbook development, synthesis of best practices, and operational collaborations for policy change to the SALIENT team. Dr. Haun led the reorganization of our proposed conceptual framework and integration of the implementation science research logic modeling and framework to projects from conceptualization to data collection, reporting, and synthesis. Drs. Pugh and Haun are MPIs on a current QUERI partnered evaluation initiative, and have an established process for collaboration, communication, and co-leadership across enterprise-wide level projects. Dr. Haun included several of her team members and collaborators from VA Tampa and Hines sites who have established implementation science expertise on several QUERI and operations-based projects. Dr. Haun's expertise and collaborative support is further enhanced by including Julie Lowery PhD as a consultant, and additional implementation science expertise (Drs. Butler, Cochran, Gordon, Kean, McAndrew, Naranjo, Rubin, Schlechter, Smith, White).

5. Additional Weaknesses Identified by Reviewers

5.a Significance and Impact

Reviewer 1 Key Weakness 2: Some of the planned impacts are less defined…it is not specified how health equity is evaluated. While health equity is no longer a priority area of this RFA, it is cross-cutting and will be included in evaluations where equity is a concern. Our investigators have expertise in health equity (i.e., Mohanty, Zickmund, A. Jones, White) and will partner with the Office of Health Equity to guide evaluations related to equity to ensure the approach and definitions most relevant to VA and Veterans is used.

Reviewer 2 Key Weaknesses 1 and 2: There was a request for more detail on operationalization of the

plan, specific methods, and description of impact. The resubmission has been rewritten to add details to the methods of the SALIENT Center objectives and individual evaluation projects. Additionally, the proposal has been integrated throughout to demonstrate how the Lean Six Sprint/Sigma process, conceptual framework, and cross core integrated approach will maximize impact on implementation science/knowledge translation, and development of best practices in policy evaluation and implementation (**B.6; C.4**). The use of our established rapid processes in conceptualization, redesign, analysis, and synthesis ensures our ability to pivot and assemble strong, effective research teams to evaluate VA priorities. The development and implementation of our WVU project demonstrated our ability to nimbly assemble strong, effective research teams to evaluate VA priorities. As such, we have provided details (**Tables 1 and 2**) to describe our expertise and capacity to conduct rapid evaluations, provided the timeline used for the WVU study (**Figure 3)**.

Due to changes in priority areas, we propose two new evaluations which clearly identify the significance and impact of each evaluation, a clear timeline and feasibility of the project, and a clear description of how the proposed plan will be operationalized including specific methods. We restructured our Methods section to include an overview of our processes **(B.2),** with more detail in the operationalization of evaluation plans **(B.3)** and core interactions.

SALIENT anticipated impact will contribute to 1) optimized policies and programs for Veteran populations; 2) improved outcomes regarding health, equity, cost, and provider well-being; 3) advances in the science of dissemination and knowledge translation by synthesizing learning across SALIENT and other center evaluations; 4) expansion of the implementation and dissemination science workforce.

Reviewer 2: It is unclear how the application fits into the overall existing program evaluation structure that already is present within VA Central Office. To address this concern, we added section **A.2.7** and **Figure 1**, which describes the role of SALIENT in the existing VA evaluation structure. We also emphasize that we have existing collaborations with teams recently funded as QUERI evaluation centers; and will continue to work with all funded evaluation centers to synergize the impact of our and other centers. We will also collaborate with said funded centers to develop best practices in policy evaluation and implementation as part of our SALIENT Policy Evaluation Catalog outcome (**Objective 3; B.6**).

Reviewer 2: How leaders will be provided with the evidence to fully assess the impact of policies should also be clarified. In our description of Objective 2, we provide a comprehensive list of established innovative approaches to communicating evaluation evidence and recommendations to leaders.

5.b Minor Concerns

Reviewer 1 Approach/Feasibility: Page 42: "The Lean tools will be used to create Value Stream Maps of schematics used to identify activities that have value to stakeholders as well as waste, delays, and inefficiencies" – What activities does this refer to? What types of inefficiencies? We thank the reviewer for the opportunity to provide an example. We have added text to **B.6** that illustrates an example of wastes, delays, or inefficiencies (i.e., batching vs. single piece workflow). Our after-action processes which we will use to elucidate such problems will allow us to meet the needs of operational partners more precisely and continuously improve our collaboration methods and provide data for analyses that will be used to develop best practices in policy evaluation and implementation.

Reviewer 2: Approach and feasibility: The application describes a wide range of approaches for conducting analyses. Although this can be a strength when flexibility of approach is a priority, for the current application, it lacks specificity and does not include an appropriate level of detail to adequately describe what is being proposed. We made multiple edits to address this concern. First, we specify the methods used (and justification for their use) for each project. Second, we added **Table 2** which describes the extensive expertise that is available for future assigned projects.

Reviewer 3 Significance/Impact: Information is not provided regarding how the projects map on to specific VA priorities. We identified the specific priority area addressed by each project in **Figure 7** and at the beginning of each project description.
